# The expression signatures in liver and adipose tissue from obese Göttingen Minipigs reveal a predisposition for healthy fat accumulation

**DOI:** 10.1038/s41387-020-0112-y

**Published:** 2020-03-23

**Authors:** Susanna Cirera, Emirhan Taşöz, Mette Juul Jacobsen, Camilla Schumacher-Petersen, Berit Østergaard Christoffersen, Rikke Kaae Kirk, Trine Pagh Ludvigsen, Henning Hvid, Henrik Duelund Pedersen, Lisbeth Høier Olsen, Merete Fredholm

**Affiliations:** 1 Department of Veterinary and Animal Sciences, Faculty of Health and Medical Sciences, University of Copenhagen, 1870 Frederiksberg, Denmark; 2 Global Drug Discovery, Novo Nordisk A/S, Novo Nordisk Park, Måløv, Denmark; 3 Ellegaard Gottingen Minipigs A/S, Sorø Landevej 302, 4261 Dalmose, Denmark

**Keywords:** Obesity, Cardiovascular diseases

## Abstract

**Background:**

Model animals are valuable resources for dissecting basic aspects of the regulation of obesity and metabolism. The translatability of results relies on understanding comparative aspects of molecular pathophysiology. Several studies have shown that despite the presence of overt obesity and dyslipidemia in the pig key human pathological hepatic findings such as hepatocellular ballooning and abundant steatosis are lacking in the model.

**Objectives:**

The aim of this study was to elucidate why these histopathological characteristics did not occur in a high fat, fructose and cholesterol (FFC) diet-induced obese Göttingen Minipig model.

**Methods:**

High-throughput expression profiling of more than 90 metabolically relevant genes was performed in liver, subcutaneous adipose tissue (SAT) and visceral adipose tissue (VAT) of male minipigs diet fed: standard chow (SD, *n* = 7); FFC diet (*n* = 14); FFC diet in streptozotocin-induced diabetic pigs (FFC_DIA_, *n* = 8). Moreover, histopathological assessment of SAT and VAT was performed.

**Results:**

12, 4 and 1 genes were highly significantly differentially expressed in liver, SAT and VAT when comparing the FFC and SD groups whereas the corresponding numbers were 15, 2, and 1 when comparing the FFC_DIA_ and SD groups. Although the minipigs in both FFC groups developed sever obesity and dyslipidemia, the insulin-signaling pathways were not affected. Notably, four genes involved in lipid acquisition and removal, were highly deregulated in the liver: *PPARG, LPL, CD36 and FABP4*. These genes have been reported to play a major role in promoting hepatic steatosis in rodents and humans. Since very little macrophage-associated pro-inflammatory response was detected in the adipose tissues the expansion appears to have no adverse impact on adipose tissue metabolism.

**Conclusion:**

The study shows that morbidly obese Göttingen Minipigs are protected against many of the metabolic and hepatic abnormalities associated with obesity due to a remarkable ability to expand the adipose compartments to accommodate excess calories.

## Introduction

Obesity has been associated with a strong predisposition to metabolic syndrome (MS) and Type 2 diabetes mellitus (T2DM). In turn, these diseases are strongly associated with non-alcoholic fatty liver disease (NAFLD) and non-alcoholic steatohepatitis (NASH). These conditions are characterized by a build-up of fat in the liver (hepatic steatosis), inflammation, fibrosis, and cell damage, and in extreme cases NASH can lead to liver cirrhosis with hepatic failure and/or hepatocellular carcinoma (HCC)^1^. The molecular events underlying the development of NAFLD/NASH are poorly understood, however, it has been shown that there is a greater impact of metabolic health on the development of NAFLD, as compared to obesity per se^2^. One determinant of metabolic health is the mechanism by which adipose tissue depots expand^3,4^. This has led to the adipose tissue expandability hypothesis stating that the capacity of an individual to expand the fat mass to store lipid is a more important determinant of obesity-associated metabolic problems than the absolute amount of adipose tissue^5^. The expandability hypothesis predicts that some individuals tend to have a bigger capacity for adipose tissue storage and adaptation to excess energy while remaining metabolically healthy (metabolically healthy obese (MHO)). This capacity is most likely determined by genetic and epigenetic factors^6^.

Different breeds of minipigs, including Bama, Ossabaw and Göttingen have been used as models for MS and obesity, and some of them specifically as models for liver disease in humans. In these studies, different Western diets and atherogenic diets have been shown to have potential for producing liver fibrosis, systemic inflammation, insulin resistance and steatohepatitis^7–13^. In general, however, these models only show very limited and primarily microvesicular hepatic steatosis as opposed to the more extensive macrovesicular type observed in humans. This indicates that inflammation and fibrosis are driven by other factors than steatosis in these porcine models.

We have previously provided evidence of genetic predisposition for an MHO-like phenotype in Göttingen Minipigs^14^. In this study, we have performed high-throughput qPCR on genes of relevance for metabolism in liver, subcutaneous adipose (SAT) and visceral adipose tissues (VAT) to characterize the transcriptional changes underlying the alterations observed in these tissues in Göttingen Minipigs fed chow, FFC diet, or FFC diet on a background of streptozotocin-induced diabetes for 13 months. The minipigs in the two groups fed the FFC diet were morbidly obese and dyslipidemic, however, key human pathological hepatic findings characterizing NAFLD/NASH were lacking. Our aim was to elucidate the molecular components underlying the histopathological changes observed in the model, and to explain the reason for the limited hepatic steatosis that characterizes the porcine models of metabolic syndrome and NASH.

## Materials and methods

### Animals

Castrated male Göttingen Minipigs (Ellegaard Gottingen Minipigs A/S, Dalmose, Denmark) (*n* = 84 in total) aged 6–7 months were weight stratified and distributed into six treatment groups and fed once daily for thirteen months. Of these, 29 pigs distributed in three groups were studied in this study. The included groups were: a lean control pigs (SD, *N* = 7) fed standard diet (Mini-pig, SDS, UK); a group fed high fat/fructose/cholesterol diet (FFC, *N* = 14) with (2%) cholesterol (5B4L) for the first five months and changed to a similar diet with 1% cholesterol (9G4U) for the next eight months (Test diet®, Missouri, USA); a streptozotocin-induced diabetic group (FFC_DIA_, *N* = 8) fed a high fat/fructose/cholesterol (1%) diet throughout the study (9G4U). Data from the same 29 pigs have been included in parallel studies focusing on histological changes in the liver tissue and myocardial changes^12,15^. Basic phenotypic and metabolic characteristics, measured as described in^12^ are reported in Table 1.

**Table 1:** Phenotypic and metabolic characteristics of the minipigs.

Diet group	SD (*n* = 7)	FFC (*n* = 14)	FFC_DIA_ (*n* = 8)	Over all *p*-value
BW (kg)	39 (38; 41)	78 (69; 81)^a^	60 (54; 64)^ab^	<.0001^˄^
LW (g)	485 (458; 564)	1732 (1067; 2219)^a^	2077 (1478; 2439)^a^	<.0001^˄^
TBF%	28 (24; 31)	64 (61; 68)^a^	55 (53; 56)^ab^	<.0001
VAT (g)	510 (462–543)	2326 (1645–2946)^a^	1335(1032–1922)^a,b^	<0001^^^^
TG in liver (mg/g)	12.6 (8.1–14.4)	15.0 (13.3–17.7)	19.6 (13.6–26.7)^a^	NS^^^
Plasma TC^#^ (mmol/L)	1.70 (1.64; 2.18)	11.94 (11,00; 13.18)^a^	18.91 (16.91; 27.00)^ab^	<0.0001^˄^
Plasma TG^#^ (mmol/L)	0.34 (0.29; 0.35)	0.63 (0.54; 0,88)^a^	1.45 (0.57; 1.72)^ab^	0.0002^˄^
Plasma GLU^#^ (mmol/L)	3.48 (3.32; 3.67)	3.72 (3.60; 3.83)	15.1 (14.67; 15.45)^ab^	<.0001^˄^

Results are presented as median and 25% and 75% quartiles; ^#^*n* = 6 for SD, *n* = 13 for FFC, *n* = 6 for FFC_DIA_ due to catheter failure;

*BW* body weight, *LW* liver weight, *TBF* Total body fat, *VAT* Visceral adipose tissue, *TC* total cholesterol, *TG* triglycerides, *GLU* glucose.

^˄^Transformed;

^^^^Non-parametric test;

^a^Significantly different from SD;

^b^Significantly different from FFC. Data except VAT have been presented previously (Andreasen et al., 2018; Schumacher-Petersen et al., 2019).

In the diabetic group (FFC_DIA_) type 1-like diabetes was induced with streptozotocin (as described in ref. ^12^). Diabetic pigs were treated subcutaneously once daily with long acting insulin (Lantus®, Sanofi A/S, Denmark) in order to maintain morning fasting blood glucose around 15 mM.

All animals were fasted overnight before euthanasia by exsanguination in deep general anesthesia (mixture of zolazepam, tiletamine, ketamine, xylazin and butorphanol). Samples from liver (left medial lobe), subcutaneous adipose (SAT) and visceral adipose (VAT) tissues were collected, snap frozen in liquid nitrogen and kept at −80 °C for expression studies.

### RNA isolation

Fifty miligram of frozen tissue were used for RNA isolation using the Tri® Reagent protocol (MRC Gene, Cincinnati, OH 45212 USA). Briefly, the tissue was homogenized in 2 ml Tri® Reagent using M-tubes in a gentleMACS™ Octo Dissociator machine (Miltenyi Biotec, Bergisch Gladbach, Germany) and processed according to the manufacturer’s instructions. RNA samples were subsequently DNAse treated using RNeasy MinElute Cleanup kit (Qiagen, GmbH, Germany). RNA from adipose tissues were isolated from 20–180 mg tissue using the method described by ref. ^16^ including DNAse I treatment.

Concentration and purity of the RNA samples were measured on a Nanodrop ND-1000 spectrophotometer (NanoDrop technologies, Wilmington, USA). RNA integrity was assessed on an Experion machine using the RNA stdSens kit. All liver samples had an RNA-quality index (RQI) between 8.10 and 9.70 (average = 9.25 ± 0.36). Adipose tissue samples with an RQI between 6.5 and 10 were included for further processing (average = 8.4 ± 0.68 for SAT and average = 8.2 ± 0.92 for VAT). One SAT sample from the FFC_DIA_ group and three VAT samples (2 from the SD and 1 the FCC_DIA_ group) were excluded from the study due to low RQI.

### cDNA synthesis

Two replicates of cDNA were prepared from each RNA sample using 500 ng of DNAse treated RNA from liver tissue samples and 100 ng of DNAse treated RNA from adipose tissue samples. Briefly 0.5 μl Improm-II^TM^ reverse transcriptase (Promega, Madison, USA), 0.25 μg 1:3 OligodT/Random primers, 2 μl 5× ImProm-II buffer, 10 units RNasin Ribonuclease inhibitor (Promega, Madison, USA), 2.5 mM MgCl2 and 2 mM dNTP were mixed with RNA in a final reaction volume of 10 μl. Reactions were incubated for 5 min at room temperature, 1 h at 42 °C and 15 min inactivation at 70 °C. Two negative controls were made for each tissue with no reverse transcriptase added (-RT control). The liver cDNA samples were diluted 1:16 and the adipose cDNA samples were diluted 1:8 prior to qPCR and stored at −80 **°**C until use.

### Primer design

The genes included in this study were selected from an *in house* obesity panel used in other projects (e.g., ref. ^17^) supplemented by additional genes of importance to liver and adipose tissue metabolism (see Table S1). Four reference genes selected according to ref. ^18^ were included for normalization for each tissue. Primers were designed using the tool “Pick Primers” in PubMed to amplify a product in the range of 100 nucleotides, and if possible, were designed to span a large intron. Primer sequences, gene names and the respective tissues profiled are available in Table S1.

### qPCR

High-throughput qPCR was performed on the Biomark HD system (Fluidigm Corporation, California, USA) using four 96.96 IFC chips. The diluted cDNA was used for pre-amplification (15 cycles for liver and SAT samples and 14 cycles for VAT samples) using TaqMan PreAmp Master Mix (Life Technologies, Nærum, Denmark). Subsequent cleanup with Exonuclease I (New England BioLabs, Herlev, Denmark) was performed according to the Fluidigm protocol (Fluidigm PN 100–5875 C1). A single modification to the standard protocol was made: we used 250 nM primer concentrations in the primer pool. Exonuclease cleaned liver and SAT cDNA were diluted 5x and VAT cDNA was diluted 10× before running the qPCR reactions using SsoFast^TM^ EvaGreen® Supermix with Low ROX (Bio-Rad Laboratories, Copenhagen, Denmark) according to the manufacturer’s instructions (PN 100–9792 B1) with the modification of using primer concentrations of 5 μM. A standard curve was established for each tissue using a dilution serial of a pool of preamplified cDNA samples. Data was obtained using the associated software. A few genes (*TGFB1*, *LDAH,* and *BCL2* for liver and *PPARG* and *CD36* for VAT) had several missing values in the Fluidigme qPCR study and were therfore re-run on the MxPro (Stratagene) platform. In addition, four of the deregulated genes (*CD36*, *FABP4*, *LPL,* and *PPARG*) were analyzed on the Mx3005P platform in all three tissues in order to make a direct comparison of expression level between tissues. In this study two additional primer sets were included for *PPARG* in order to amplify isoform 1 and isoform 2 separately (see Table S1). For all genes run on the Mx3005P platform, QuantiFast SYBRGreen master mix (Qiagen, GmbH, Germany) was used according to the manufacturer’s protocol.

### qPCR data processing and statistical analysis

qPCR raw data was pre-processed using Genex 6 Pro software (MultiD Analyses AB, Götteborg, Sweden). Briefly, data was corrected according to PCR efficiency (PCR efficiencies between 80–110% were accepted), data was normalized to the two most stable reference genes (*TBP* and *ACTB* in liver and SAT; *TBP* and *YWHAZ* in VAT). Subsequently, technical cDNA replicates were averaged and relative expression was calculated by scaling data with the lowest expressed sample for each assay. Next, data was log2 transformed to achieve normal distribution before statistical analysis. Multiple test correction was applied to the analysis and statistical significance threshold was set at *P* < 0.0006 (according to the Dunn-Bonferroni correction for multiple testing). Comparisons between the different experimental groups were performed using *t*-test and fold-changes (FC) and *P* values under < 0.05 were reported (Tables 1, 2, 3).

**Table 2:** Differential expression in the liver.

FFC vs. SD			FFC_DIA_ vs. SD		
Genes	FC	*P* value	Genes	FC	*P* value
**LPL**	172.23	1.00E−08	**LPL**	308.25	1.00E−08
**CD68**	5.17	1.00E−08	**CD68**	6.30	1.00E−08
**PPARG**	19.08	1.60E−08	**PPARG**	35.56	1.70E−07
**KLB**	−22.24	7.69E−06	**FDFT1**	−16.30	5.04E−07
**HMGCR**	−13.36	2.28E−05	**KLB**	−36.89	3.74E−06
**FDFT1**	−6.31	3.76E−05	**CD36**	6.30	3.94E−06
**LDLR**	−5.92	3.82E−05	**SOD1**	−2.02	1.49E−05
**SOD1**	−1.76	5.02E−05	**AGT**	−2.80	2.45E−05
**CD36**	3.36	8.79E−05	**TLR4**	2.89	3.06E−05
**STAT3**	−1.92	0.000371384	**HMGCR**	−14.26	4.28E−05
**PPARGC1A**	−2.38	0.000488187	**FABP4**	11.81	9.91E−05
**MTTP**	−1.73	0.00052084	**MMP9**	7.80	0.000166417
FABP4	2.56	0.001362823	**GHR**	−2.65	0.000416258
APOB	−1.57	0.001397134	**LDLR**	−4.38	0.000488511
TLR4	1.89	0.001562476	**GCKR**	−2.15	0.00056221
MMP9	6.02	0.001698359	TM6SF2	−1.53	0.00061856
GCKR	−1.66	0.00214768	SREBF1	3.65	0.001299769
TM6SF2	−1.31	0.002397241	PNPLA3	−5.26	0.001575959
AGT	−1.69	0.002518911	SCARB1	−1.59	0.001871992
BCL2	2.14	0.002743402	LCAT	−3.00	0.00205255
CCL5	1.58	0.003410192	LPIN1	−2.84	0.002087286
RBP4	−1.70	0.003613226	IL1B	−3.60	0.002132827
IRS2	−1.73	0.003906641	RORA	−2.01	0.002903257
SREBF1	2.60	0.004448657	MBOAT7	−1.44	0.002998874
PCSK9	−2.57	0.004967516	IRS2	−1.94	0.003022747
NR1I2	−1.48	0.004979217	STAT3	−2.01	0.003147541
GLUT2	−1.81	0.005005041	RBP4	−1.79	0.004035618
GNMT	−2.19	0.005392574	DGAT2	−1.73	0.004160409
APOC3	−1.69	0.006004771	BCL2	2.63	0.004241219
ACACA	−1.61	0.006809141	TNF	−2.96	0.005578895
MBOAT7	−1.30	0.007224475	PPARGC1A	−2.63	0.005607015
IGFBP2	−1.86	0.008639175	NR1I2	−1.49	0.006846227
LPIN1	−2.02	0.010294258	APOA4	6.29	0.007329076
INSIG1	−2.80	0.010494044	LEPR_01	2.55	0.008111383
MCM5	1.67	0.018981469	APOA1	−1.65	0.008870278
RORA	−1.63	0.021173678	FGFR4	−1.70	0.009941781
SCARB1	−1.38	0.021396819	APOC3	−1.66	0.014547442
DGAT2	−1.70	0.024872243	PCSK9	−2.67	0.015028567
SCD	1.80	0.025667474	TGFB1	1.72	0.016112059
COL1A1	2.49	0.028417769	MTTP	−1.47	0.017813835
SCAP	−1.28	0.032446191	APOB	−1.40	0.022577003
TIMP1	1.87	0.032793714	GLUT2	−1.84	0.024123063
GHR	−1.82	0.034630017	CTGF	2.16	0.035424956
INSR	−1.32	0.035708148	FOXO1	−1.49	0.043783298
LDAH	−1.37	0.04920525	SCD	1.96	0.043939123
			COL1A1	2.56	0.045340498

*FC* fold change; *P*-values: all genes with *p* < 0.05 are listed (ranked according to *P*-values): genes highlighted in bold have FC > 2 or < −1.5 and multiple testing-corrected *P* values <0.0006; A positive FC indicates that the gene is upregulated in the FFC and FFC_DIA_ groups, respectively, and a negative FC indicates that the gene is downregulated in the FFC and FFC_DIA_ groups, respectively.

**Table 3:** Differential expression in SAT and VAT.

(a) SAT					
FFC vs. SD			FFC_DIA_ vs. SD		
Genes	FC	*P* value	Genes	FC	*P* value
**PN-1**	2.05	3.20E−05	**ABCG1**	3.89	0.00011538
**EBF2**	−1.69	0.00010886	**IL6**	2.67	0.00035439
**ABCG1**	2.64	0.00022215	ABCA1	2.63	0.00067173
**IL6**	2.87	0.00043198	PN-1	2.77	0.00137394
GNAS	−1.57	0.00070595	MYC	1.52	0.0089967
SMPDL3A	3.03	0.00136871	SMPDL3A	2.98	0.01752013
FADS1	−2.18	0.00517238	ISLR	−1.82	0.02213799
ISLR	−2.23	0.00629454	ADCY5	1.74	0.02372845
ELOVL4	−2.00	0.01003605	GLUT4	−2.27	0.02893844
LEP	2.27	0.01139943	PON1	3.42	0.03258829
SP1	−1.59	0.0206303	SMAD6	1.48	0.03903247
DGAT2	−1.94	0.0210936	CD36	1.49	0.04384441
GLUT4	−2.57	0.02112195			
CXCR4	2.06	0.02381762			
MYC	1.62	0.02602224			
PELI2	−1.42	0.02939702			
HPRT1	6.24	0.03237427			
CD36	1.37	0.03572498			
DICER1	−1.78	0.03598225			
LDLR	−1.76	0.03737362			
LCN2 (NGAL)	−1.89	0.03992909			

(b) VAT					
FFC vs. SD			FFC_DIA_ vs. SD		
Genes	FC	*P* value	Genes	FC	*P* value
**LDLR**	−2.17	0.00053665	**RPS29**	−2.28	5.88E−07
RPS29	−1.42	0.00282892	ABCA1	2.27	0.00151131
ABCA1	2.09	0.00375619	LDLR	−1.96	0.01065563
TLR4	1.62	0.00451224	ABCG1	2.44	0.01258091
ACTB	1.54	0.01270316	IRS1	1.52	0.02980169
LITAF	1.25	0.01489724	LEPR	−4.24	0.03295196
LSS	−1.81	0.02886919			
FAS	−1.53	0.03170324			
TGFB1	1.50	0.03959778			

For the metabolic and physical measurements, group differences were evaluated using ANOVA and post hoc *t*-test. Parameters were transformed if they did not meet model requirements. Kruskal-Wallis test with Wilcoxon Rank-sum post hoc test (non parametric) was used if transformed data did not meet model requirement even after transformation. Data and results are presented as median and 25% and 75% quartiles. Values of *P* < 0.05 were considered statistically significant.

Pearson correlation analyses were performed on gene expression data for *CD36*, *FABP4*, *LPL,* and *PPARG* in the liver tissue and relevant metabolic/physical measurements using Rstudio^19^. For the Pearson correlation analysis, log2 transformed expression values were further processed using R scripts, together with the Göttingen Minipig physiological measurements

### Histology

VAT and SAT tissue samples were fixed in formalin and subsequently embedded in paraffin following standard procedures. Sections of 3 µm thickness were cut from SAT and VAT and routinely stained with hematoxylin and eosin (HE) as described previously^12^.

## Results

The expression profiles of 96 genes in liver, and 98 genes in SAT and VAT samples were assayed on the Fluidigm high-throughput qPCR platform and/or on the Mx3005P 96-format platform (see Table S1 for details). Since the *PPARG* transcript generates two isoforms (*PPARG1* and *PPARG2*) three primer pairs were used for the amplification of the transcripts. The *PPARG* primers amplify both isoforms whereas *PPARG1* and *PPARG2* amplify the respective isoforms. The total number of successful assays (including reference genes) was: 82 in liver, 86 in SAT and 90 in VAT. Fourteen assays in liver, 12 in SAT and eight in VAT failed due to one of the following reasons: unspecific amplification as evidenced by more than one peak in the melting curve; expression levels under the limit of detection; qPCR efficiencies out of range (<80% or >110%) or no amplification. Raw Cq values for all successfully profiled genes in liver, SAT and VAT are presented in Tables S2, S3, and S4 respectively. All differentially expressed genes for which *P* < 0.05 are listed in Tables 2, 3, 4. In the following only differentially expressed genes with fold changes (FC) > 2 or <−1.5 and multiple testing-corrected *P* values < 0.0006 will be discussed together with a few genes of interest having differential expression with *P* < 0.05.

**Table 4:** Correlation between adipose tissue expansion and expression of selected genes in the liver.

**TBF%**	**TBF%**						
VAT (g)	0.81***	**VAT (g)**					
TG in liver			**TG in liver**				
*CD36*	0.67**			***CD36***			
*FABP4*				0.69**	***FABP4***		
*LPL*	0.81***	0,6*		0.95***	0.65*	***LPL***	
*PPARG*	0.76***			0.95***	0.64*	0.94***	***PPARG***

*TBF* Total body fat, *VAT* Visceral adipose tissue, *TG* triglycerides.

*P < 0.001;

**P < 0.0001;

***P < 0.00001.

### Differential expression in liver

As shown in Table 2, when looking at gene expression in the liver of animals subjected to the FFC diet vs. the SD diet 12 genes showed significant *P* values after correction for multiple testing with FC of >2 or < −1.5. Four of these genes were upregulated (*LPL* (FC = 172.23), *CD68* (FC = 5.17), *PPARG* (FC = 19.08) and *CD36* (FC = 3.36), and eight were downregulated (*KLB* (FC = −22.24), *HMGCR* (FC = −13.36), *FDFT1* (FC = −6.31), *LDLR* (FC = −5.92), *SOD1* (FC = −1.76), *STAT3* (FC = −1.92), *PPARGC1A* (FC = −2.38) and *MTTP* (FC = −1.73)). In addition, 33 genes were deregulated with *P* < 0.05 and of those, 21 genes had FC > 2 or > −1.5; among these *FABP4* with FC = 2.56 (see Table 2). A total of 9 out of the 12 genes deregulated between the FFC and the SD groups were also deregulated between the FFC_DIA_ and the SD groups. The differential expression between these two groups was, however, much higher for some of the genes, i.e., FC = 308.25 for *LPL*; FC = 35.56 for *PPARG*; FC = −16.30 for *FDT1*; FC = −36.89 for *KLB*; FC = −6.30 for *CD36;* and FC = 11.8 for *FABP4* indicating that induction of diabetes had additional impact on perturbation of liver metabolism compared to the FFC diet alone. This is further supported by the fact that additional genes obtained multiple testing corrected *P* values < 0.0006 (*AGT*, *TLR4*, *FABP4*, *MMP9*, *GHR*, and *GCKR*) (see Table 2). Generally, the insulin-signaling pathways were not affected neither in the FFC diet group nor in the FFC_DIA_ diet group as documented by an unchanged level of transcription of e.g., *IDE*, *INSIG1*, *INSIG2*, *IGF1*, *IGF2*, *IGFBP2,* and *IRS1*. Only *IRS2* was slightly downregulated in both the FFC and the FFC_DIA_ groups (FC = −1.73 and −1.94, respectively) and *INSR* was slightly downregulated in the FFC group. (FC = −1.32).

### Differential expression in adipose tissues

As shown in Table 3a, b the fold changes of the differentially expressed genes were much lower in the adipose tissues relative to the differential expression observed in liver. There was only four (*PN-1*, *EBF2*, *ABCG1*, *IL6*) and two (*ABCG1*, *IL6*) genes with FC > 2 or < −1.5 in SAT when comparing FFC vs. SD and FFD_DiA_ vs. SD, respectively with *P* values passing multiple testing. In VAT only one gene with FC > 2 or < −1.5 and *P* values passing multiple testing was seen in the FFC vs. SD and FFD_DIA_ vs. SD comparisons (*LDLR* and *RPS29* respectively). SAT presented a higher number of differentially expressed genes compared to VAT in response to both the FFC and FFC_DIA_ diet. A common trend in the two adipose tissues is that genes involved in lipid homeostasis are upregulated. I.e., *ABCG1* in SAT (FC = 2.64 in FFC vs. SD; FC = 3.89 in FFC_DIA_ vs. SD); *ABCA1* in SAT (FC = 2.63 in FFC_DIA_ vs. SD), and in VAT (FC = 2.09 in FFC vs. SD; FC = 2.27 in FFC_DIA_ vs. SD). Furthermore, *IL6* is upregulated in SAT (FC = 2.87 and FC = 2.67 in FFC vs. SD and FFC_DIA_ vs. SD, respectively).

### Relative expression of selected genes in liver, SAT, and VAT

**Fig. 1:**
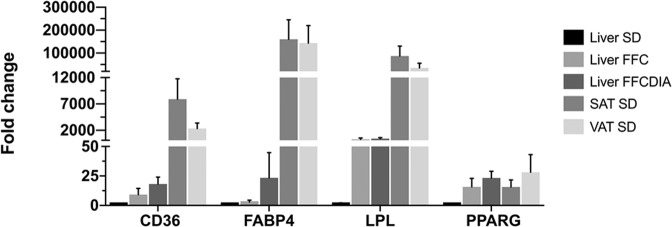
Comparison of the relative expression of *CD36*, *FABP4*, *LPL* and *PPARG* in the liver from SD, FFC, and FFC_DIA_ fed pigs, and in SAT and VAT from SD fed pigs. The relative expression is provided in fold change and compared for each of the mentioned genes using the colour scheme shown in the right hand top corner of the figure.

The expression of *PPARG* and its target genes (*FABP4, LPL*, and *CD36*) were unchanged in SAT and VAT, but highly differentially expressed in the liver (see Tables 2, 3, 4 and Tables S2, S3, S4). To compare the relative expression levels between liver, SAT, and VAT the four genes were assayed using the Mx3005P 96-format platform. The expression levels of the genes in the liver from the SD group of pigs were used as baseline. All three primer pairs amplifying the isoforms of *PPARG* were included. Both *PPARG* isoforms (*PPARG1* and *PPARG2)* were expressed in the three tissues. *PPARG1* was slightly, but not significantly, higher expressed in the liver compared to *PPARG2*, whereas *PPARG2* was slightly, but not significantly, higher expressed compared to *PPARG1* in SAT and VAT (see Table S5). As illustrated in Fig. 1, *CD36*, *FABP4,* and *LPL* were, as expected, expressed at a much higher level in the adipose tissues whereas *PPARG* expression in the liver of the FFC and FFC_DIA_ groups were comparable to the expression in the adipose tissues.

### Correlation between adipose tissue expansion and expression of selected genes in liver

A Pearson correlation analysis was performed to investigate the possible association between expression of *PPARG* and its target genes in the liver and increasing fat deposition.

As seen in Table 4, the expansion of body fat and visceral fat were significantly correlated (*r* = 0.81, *p* < 0.00001). Furthermore, expression levels of *PPARG*, *LPL,* and *CD36* were significantly correlated (*p* < 0.00001, *p* < 0.00001, and *p* < 0.0001, respectively) with total body fat (*r* = 0.76, 0.81, and 0.67, respectively), whereas, only *LPL* expression was moderately correlated with VAT (*r* = 0.6, *P* < 0.001). Expression of the four genes was correlated with highly significant correlation between *PPARG, LPL* and *CD36* (*r* > 0.9; *P* < 0.00001), and moderately significantly correlation with *FABP4* (*r* > 0.6, *P* < 0.001). As expected no correlation was found between any of the parameters and TG in liver.

### Histopatholology and expression of pro-inflammatory cytokines

**Fig. 2:**
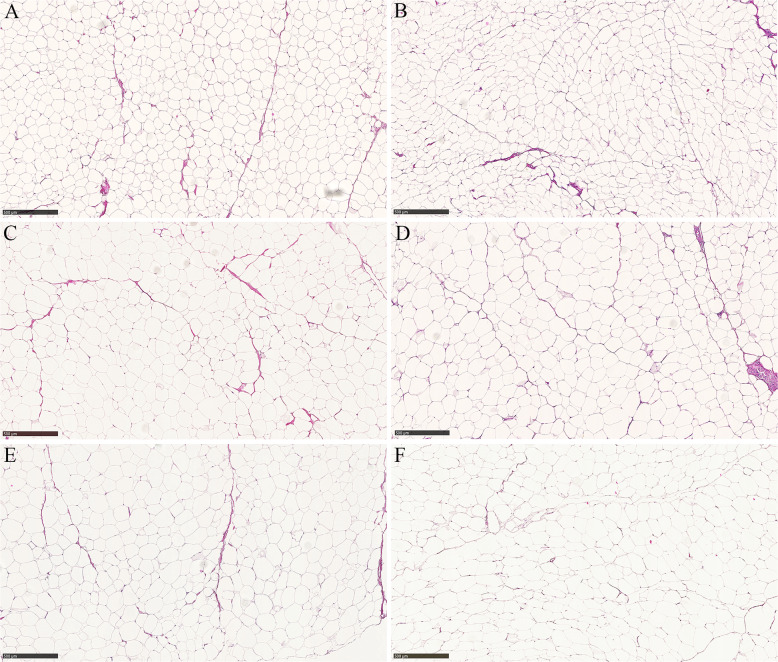
Examples of adipocytes in SAT and VAT from representative pigs. **a** (SAT) and **b** (VAT) from a pig subjected to the SD diet; **c** (SAT) and **d** (VAT) from a pig subjected to the FFC_Dia_ diet; **e** (SAT) and **f** (VAT) from a pig subjected to the FFC diet. Scale bar 500 µm. Hematoxylin and eosin staining.

Histopathological examination of sections from SAT and VAT revealed no increase in macrophage infiltration in the adipose tissues from the pigs subjected to the FFC diet relative to the pigs on the SD diet and Crown-like structures were not observed (see Fig. 2). These findings are supported by the expression profiles of the tissues, that is, of the pro-inflammatory transcripts examined (*IL18, IL1B, IL6, TLR4*, and *TNF*) only *IL6*, was upregulated in SAT (FC = 2.86 and 2.67 in FFC vs. SD and FFC_Dia_ vs. SD, respectively) whereas none of them were upregulated in VAT.

## Discussion

In this study we have profiled expression of genes of relevance for metabolism in liver, subcutaneous adipose (SAT) and visceral adipose tissues (VAT) to characterize a Göttingen Minipig model of metabolic syndrome and NASH.

As shown in Table 1, the FFC and FFC_DIA_ groups developed obesity with high body weight, high total body fat % and dyslipidemia (i.e., increased triglyceride (TG) and total cholesterol (TC) levels in plasma)^12^. In the study by ref. ^12^, it was also shown that the FFC diet resulted in development of hepatomegaly with hepatic fibrosis, inflammation, cytoplasmic alterations, and increased content of cholesterol, whereas no difference in triglyceride content in the liver was found. Thus, hallmarks of human NAFLD/NASH like severe steatosis and hepatocellular ballooning were lacking. Inducing diabetes on top of the FFC diet did not exacerbate the histopathological findings compared to the FFC diet^12^. Both the histopathological findings and the results of the expression studies clearly document that the FFC diet challenged the metabolism in the liver. That diet rather than obesity per se is the driving factor is supported by a previous study in which hepatic differential expression between Göttingen Minipigs fed standard minipig chow restrictively (lean controls) and Göttingen Minipigs fed the same diet ad libitum (obese) was studied^20^. Although, these treatments resulted in an obese group obtaining roughly the same body weight as the FFC diet groups included in this study, liver metabolism was far from affected at the level documented here, indicating that the diet components play a more important role than obesity per se.

The increased inflammation observed in the liver in the FFC and FFC_DIA_ groups^12^ was reflected in the expression study, i.e., both the *CD68* and *TLR4* transcripts were upregulated (the latter only in the FFC_DIA_ group). It is however noteworthy that staining with allograft inflammatory factor-1 (IBA1) only revealed moderate infiltration of macrophages in the liver of the animals subjected to the FFC diet^12^ perhaps explaining why in this study none of the transcripts representing pro-inflammatory cytokines (e.g., *TNF, IL1B, IL18, IL6*) were upregulated in the liver of these animals. The increased content of collagen in liver detected in the precious study^12^ is in concordance with the increased expression of the *MMP9* transcript which encodes a protein that can cleave different types of collagen.

Abundant hepatic steatosis, which is lacking in our model, arises from an imbalance between triglyceride acquisition and removal. In particular, four of the genes that were upregulated in the liver have been reported to play an important role in this context: *PPARG*, and three of its target genes, i.e., *LPL* and *CD36*, and *FABP4*. Under normal physiological conditions these genes are expressed almost exclusively in adipose tissues in human and mouse. In human studies the two isoforms of *PPARG* have been shown to have different tissue distribution, i.e., *PPARG1* is expressed in a wide variety of tissues, while *PPARG2* is mainly expressed in adipose tissues (reviewed by ref. ^21^). In this study, we have shown that the two isoforms are expressed at almost the same level in the FFC and FFC_DIA_ groups of pigs in both liver, SAT and VAT. Both isoforms are lipogenic transcription factors that function as inducers of adipocyte differentiation and several lines of evidence suggest that *PPARG* activation causes insulin sensitization in adipocytes (e.g., ref. ^22,23^). The implications of an increased expression level of *PPARG* in liver are less well documented. It has, however, been shown that *PPARG* expression is elevated in the liver of mice that develop fatty liver^24^, and *PPARG* has been reported to play a major role in promoting hepatic steatosis in mice^25^. Both *PPARG1* and *PPARG2* also appear to be upregulated in liver during the pathogenesis of NAFLD in humans^26^. The increased expression of *FABP4*, *CD36*, and *LPL* suggests increased fatty acid uptake, transport, and metabolism in the livers of the FFC diet fed pigs. *FABP4* expression in liver has been shown to be significantly elevated in mouse models of obesity-promoted hepatocellular carcinoma and in patients with underlying hepatic steatosis resulting from NAFLD^27^. Also, increased expression of *CD36* in the liver has been shown to occur in response to diets rich in fatty acids, and this appears to increase hepatic fatty acid uptake and exacerbates both hepatic storage and secretion of triglyceride^28^. LPL plays a critical role in regulating lipid metabolism and tissue-specific effects are still being explored. Tissue specific overexpression of LPL in skeletal muscle and liver in mice has been reported to increased cellular stores of triglycerides leading to insulin resistance^29^. Contrasting, a more recent study in mice has shown that hepatic LPL is involved in the regulation of plasma LPL activity and lipid homeostasis^30^. Our results show that Göttingen Minipigs do not develop abundant hepatic steatosis in spite of the significantly increased expression of *PPARG, FABP4*, *CD36*, and *LPL* in the liver. Thus, it might be speculated that the ectopic expression of these genes are consequences of high fat diet/obesity rather than the cause of development of steatosis in the liver. We cannot rule out that longer term FFC diet treatment/obesity might lead to adverse metabolic responses. Still, abundant steatosis is not an immediate outcome of the highly increased expression level of *PPARG, FABP4*, *CD36*, and *LPL* in the liver of the severly obese minipigs. Conversely, since *PPARG* and *LPL* expression in liver is significantly correlated with the amount of body fat (see Table 4) the ectopic expression of the genes might have an influence on the repartitioning of lipid from liver to adipose tissues. This is also in keeping with the fact that the liver in pigs, in contrast to humans and rodents, is not the primary site of de novo lipogenesis^31^.

The ability of the Göttingen Minipigs to sustain the diet challenges is also reflected in differential regulation of genes involved in cholesterol biosynthesis in the liver. I.e. *HMGCR* and *FDFT1* are deregulated with a highly negative FC in the liver. Both genes are key regulators of cholesterol biosynthesis and the observed downregulation can most likely be explained by the abundance of cholesterol in the diet. *KLB* is also deregulated with a highly negative FC. KLB contributes to repression of CYP7A1—a rate limiting enzyme in the bile acid biosynthesis pathway that converts cholesterol into bile acids^32^. Thus, the deregulation of the genes involved in cholesterol biosynthesis appears to assist in rectifying the diet-induced increase in cholesterol through conversion of cholesterol into bile acids.

As seen in Table 1, both body fat and visceral fat content were highly significantly increased in the FFC diet fed groups, nevertheless, metabolism in the adipose tissues was not affected to a great extent, although more so in SAT compared to VAT. It is generally accepted that metabolism differs between SAT and VAT, and that excess VAT is unhealthier than excess SAT (e.g., ref. ^33^). The increased expression of *IL6* in the fat tissues is an indication of low-grade inflammation; however, adipogenesis does not seem to be severely adversely affected by the FFC diet neither in SAT nor in VAT. I.e., none of the insulin sensitizing (e.g., *ADIPOQ*) or resistance genes (e.g., *TNF*) were perturbed. Rather, mainly genes involved in lipid and cholesterol homeostasis (*ABCA1, ABCG1*) were upregulated in these tissues. Lipid metabolism in the fat tissue is supported by the high unchanged expression level of *PPARG, FABP4, CD36*, and *LPL* in these tissues (se Tables S2, S3, S4 and Fig. 1). Although both adipose tissue compartments were substantially expanded in the groups on the FFC diet, the highly significant correlation between body fat and expression of *PPARG, LPL*, and *CD36* in the liver indicates, as previously mentioned, that ectopic expression of these genes supports adipogenesis/lipogenesis in body fat compartments rather than in the liver. In contrast, obese human subjects with a high degree of metabolic endotoxemia have been shown to have lower expression of key genes for adipose tissue function and lipogenesis (*SREBP1*, *FABP4*, *FASN*, and *LEP*), but higher expression of inflammatory genes in VAT and SAT^34^. In contrast to this, the only pro-inflammatory cytokine upregulated in the obese Göttingen Minipigs was *IL6*, which was upregulated in SAT but not in VAT. This is supported by the histopathological examinations which did not reveal an increase in macrophage infiltration in SAT and VAT in the obese pigs (see Fig. 2). Also, in contrast to our findings, the expression of *PPARG* has been shown to be significantly downregulated in SAT in severly obese women^35^. Thus, our study shows that Göttingen Minipigs are able to maintain fatty acid synthesis, and expand the fat compartments without compromising adipose tissue metabolism. Our results support the notion that the capacity to expand fat mass to store lipids is a more important determinant of obesity-associated metabolic problems than the absolute amount of adipose tissue, as has also been shown in humans (reviewed by ref. ^36^). The importance of the adipose tissue expandability is further supported by studies that have used thiazolidinediones (TZDs) to treat NAFLD and NASH and reverse insulin resistance in target tissues. These studies have demonstrated good efficacy of TZDs to reduce lipid content in the liver concordant with adipose tissue expansion^37,38^. TZDs are potent PPARG agonists^39^ and thus, the proposed role for PPARG as an inducer of steatosis in hepatocytes appears conflicting with the efficacy of TDZs in terms of reducing hepatic lipid content. On the other hand, it is important to note that the main target tissues for TDZs are adipose tissues and, as also suggested by ref. ^5^, the expandability of the adipose tissue explains how TDZs can be beneficiary for NASH since the increasing capacity of adipose tissue to store fat allows repartitioning of lipid from liver to adipose tissue. Since Göttingen Minipigs are able to expand the fat compartments without compromising adipose tissue metabolism it might be hypothesized that naturally occurring fatty acids activate PPARG in the liver of the FFC diet treated pigs, mimicking treatments with TDZs, resulting in repartitioning of lipid from liver to adipose tissues. Our findings are in agreement with a previous study showing that haplotypes segregating from Göttingen Minipigs can uphold a healthy lipid profile despite development of obesity indicating they have a phenotype comparable to the MHO phenotype in humans^14^.

In conclusion, our study shows that severly obese Göttingen Minipigs have a large capacity for adipose tissue expansion and are protected against many of the metabolic and hepatic abnormalities associated with obesity. The study lends support to the hypothesis that adipose tissue expandability and adaptation plays a crucial role in the maintenance of metabolic homeostasis and elucidates some of the molecular components underlying the MHO-like phenotype in Göttingen Minipigs. In contrast to what has been reported in human and mouse studies, the highly significant upregulation of *PPARG, CD36, LPL,* and *FABP4* in the liver of the minipigs do not result in development of abundant hepatic steatosis. The coordinated activation of lipid uptake and lipid biosynthesis by *PPARG* in the liver appears to be balanced by the ability of the adipose tissues to expand and store excessive calories. Although our study shed light on some of the mechanisms that disassociate obesity from metabolic complications a large number of questions, for instance, regarding how adipose tissue plasticity is regulated still remain. Identification of additional underlying factors associated with the metabolic healthy obese phenotype in Göttingen Minipigs can contribute to a better understanding of the factors that predispose, delay or protect obese individuals from metabolic disturbances.

## Supplementary information

Supplementary Information

S1 Table

S2 Table

S3 Table

S4 Table

S5 Table
