# Supplementary material for: The expression signatures in liver and adipose tissue from obese Göttingen Minipigs reveal a predisposition for healthy fat accumulation
Source: Nutr Diabetes. 2020 Mar 23;10:9. doi: 10.1038/s41387-020-0112-y (PMC7090036; doi:10.1038/s41387-020-0112-y)
Supplement: Supplementary file 1 — Supplementary Information [file 41387_2020_112_MOESM1_ESM.docx]

**Supplementary information**

**S1 Table. Gene abbreviations, gene names, primer sequences, and tissue**

**S2 Table. Raw qPCR data from liver**

**S3 Table. Raw qPCR data from SAT**

**S4 Table. Raw qPCR data from VAT**

**S5 Table. Raw qPCR data from four selected genes (Mx3005P 96-format platform)**
