# Supplementary material for: The expression signatures in liver and adipose tissue from obese Göttingen Minipigs reveal a predisposition for healthy fat accumulation
Source: Nutr Diabetes. 2020 Mar 23;10:9. doi: 10.1038/s41387-020-0112-y (PMC7090036; doi:10.1038/s41387-020-0112-y)
Supplement: Supplementary file 2 — S1 Table [file 41387_2020_112_MOESM2_ESM.pdf]

Gene abbreviation	Gene Name	Forward primer	Reverse Primer	Tissue
ABCA1	ATP Binding Cassette Subfamily A Member 1	AGCCCTGGATGTACAACGAA	TGGGTTCCCTTCCATACACC	VAT and SAT
ABCC2	ATP Binding Cassette Subfamily C Member 2	GATGCTCACGTGGGAAGACA	GTGCCATTTCCCACAACCAC	Liver
ABCG1	ATP Binding Cassette Subfamily G Member 1	TCAGGAGGGAGGACAACAAC	ACCCTTTCTTCCTCCACCAG	VAT and SAT
ACACA	Acetyl-CoA Carboxylase Alpha	GGCCATCAAGGACTTCAACC	ACGATGTAAGCGCCGAACTT	Liver, VAT and SAT
ACTA2	Alpha Actin-2, Alpha Smooth Muscle Actin	GTTTTCCCGTCCATTGTGGG	TTTGCTTTGGGCTTCGTCAC	Liver
ACTB	Beta Actin, Reference Gene	TCTGGCACCACACCTTCT	TGATCTGGGTCATCTTCTCAC	Liver, VAT and SAT
ADCY5	Adenylate Cyclase 5	TCTGGGGCAATACGGTGAAC	CACTCCAGCTGGTACGTGTT	VAT and SAT
ADIPOQ	Adiponectin, C1Q and Collagen Domain Containing	CGAGAAGGGTGAGAAAGGAG	TAGGCGCTTTCTCCAGGTTC	Liver, VAT and SAT
ADIPOR1	Adiponectin Receptor 1	ATTCCTGAGCGCTTCTTCCC	CGTAGCGGAACTCCTGAAGG	Liver
ADM	Adrenomedullin	CCGAAAGAAATGGAATAAGTGG	GAGAGGAGCCCTTCACATCC	VAT and SAT
ADRBK2	Beta-Adrenergic Receptor Kinase 2	CTCCTGGAAAGCTTGCTCCA	AGCAGCATTGACTTCTCCCC	VAT and SAT
AGT	Angiotensinogen	CCAGACAGCACCCTACTCCT	GTGTTGTCCACCCGGAACTC	Liver
AKT2	AKT Serine/Threonine Kinase 2	AGTACCTGCACTCGAGGGAC	TCCGTGATCTTGATGTGGCC	VAT and SAT
ANGPTL4	Angiopoietin Like 4	CTTGGGATCAGGACCACGAC	ACTGGCCATTGAGGTTGGAG	VAT and SAT
APOA1	Apolipoprotein A-I	GTTCTGGGACAACCTGGAAA	GCTGCACCTTCTTCTTCACC	Liver
APOA4	Apolipoprotein A-IV	CAGGATGTTCCTGAAGGCTGT	TAGCCACTTGGTCGGCATTG	Liver
APOB	Apolipoprotein B-100	TGTCCAAGTACGAGCTCAGG	GCAGGAGGGCAGAAATGATG	Liver
APOC3	Apolipoprotein C-III	CGCTAACCAGCGTGAAGGAG	TGAACGTGCTCCAGTAGTCC	Liver
AQP7	Aquaporin 7	CCGCTTCTTCACCTTCATTG	TCCAGTATCTGGGGTTCCTG	VAT and SAT
ATGL	Patatin Like Phospholipase Domain Containing 2	CCAACGCCAAGCACATCTAC	TTCACCAGGTTGAAGGAGGG	Liver
BCL2	Apoptosis Regulator Bcl-2	GGATCCAGGATAACGGAGGC	TGATGCAAGCTCCCACCAG	Liver (MxPro)
CCL2	C-C motif Chemokine 2	GCAAGTGTCCTAAAGAAGCAGTG	TCCAGGTGGCTTATGGAGTC	Liver
CCL5	C-C motif Chemokine 5	GGCACAGACTCCACCACTTA	CCCTTCTCCCTCTCCTCTTC	Liver
CD36	CD36 Antigen (Collagen Type I Receptor)	TGAGTGTTGGTGTGGGGATG	GTCCTGGGGTCTGTGAGTTG	Liver, VAT and SAT
CD40	CD40 Molecule, TNF Receptor Superfamily Member 5	TGAGAGCCCTGGTGGTTATC	CCTCCAGATCAATCGTCTCC	VAT and SAT
CD68	CD68 Molecule, Macrophage Antigen	CGAGATCTCCAAACGCCTGT	AGAGGCAGCAGGATGGTTTG	Liver
CLU	Clusterin	GCAGAATGACGACCGCTACT	GACTTCCTGCGGGATAATCA	VAT and SAT
COL1A1	Collagen Alpha-1(I) Chain	CAACGAGATCGAGATCCGGG	TTCGATCACTGTCTTGCCCC	Liver
COL6A1	Collagen Type VI Alpha 1 Chain	TGATAGACACAAGGGCCGTC	AGGACTTGACCTTGTCCACC	VAT and SAT
CTGF	Connective Tissue Growth Factor	TCAGGCCTTGTGAAGCTGAC	AGCTCAAACTTGACGGGCTT	Liver
CTP1A	Carnitine Palmitoyltransferase 1A	AGCAGGTGGAGCTGTTTGAT	GACACCCCATAACCATCGTC	Liver
CXCL14	C-X-C Motif Chemokine Ligand 14	AAGCCAAAGTACCCGCACTG	TTCCAGGCGTTGTACCACTT	VAT and SAT
CXCR4	C-X-C Motif Chemokine Receptor 4	GTGAGCAGTTACCATGGACG	GTCATAGTCACCTGAGCCCA	VAT and SAT
DGAT2	Diacylglycerol O-acyltransferase 2	TACTTCACTTGGCTGGCGTTT	CACCAGCTGGATGGGAAAGTAG	Liver, VAT and SAT
DICER1	Dicer 1, ribonuclease III	TGGCTCAGGGAAGACGTTTA	AAGAACACCGTCCTTTTGCC	VAT and SAT
DIO1	Deiodinase, Iodothyronine Type I	GTGGTGGTGGACACAATGAA	CTTTTCCAGAACAGCACGAA	VAT and SAT
DUSP10	Dual Specificity Phosphatase 10	AAGCCAAGTGAATGAGGGCT	GAGGCATGAGGAGGCTGAAA	VAT and SAT
EBF2	Early B Cell Factor 2	TCCACCCCTCAGCAGTCTAA	GATGGAGGACGTGCTGGAAG	VAT and SAT
ELOVL4	ELOVL Fatty Acid Elongase 4	TATGGGCTAACTGCCTTTGG	CGATGGTCACATGGAACTGA	VAT and SAT
ELOVL6	ELOVL Fatty Acid Elongase 6	AGAGAACACGTAGCGACTCC	CCGACCGCCAAAGATAAAGG	VAT and SAT
FABP4	Fatty Acid Binding Protein 4	GGGCCAGGAATTTGATGAAG	CTTTCCATCCCACTTCTGCAC	Liver, VAT and SAT
FADS1	Fatty Acid Desaturase 1	CACTGGTTTGTGTGGGTCAC	CGCTGAACCAGTCATTGAAA	VAT and SAT
FAS	Fas (TNF Receptor Superfamily Member 6)	CCACGTGTGAACATGGAGTC	TCTTCTGCACCGTCTTTTCA	VAT and SAT
FASN	Fatty Acid Synthase	CTGGAGGAGTTCTGGGCCAAC	TTCGCCTGCTTGGAGTGGAC	Liver, VAT and SAT
FDFT1	Farnesyl-diphosphate Farnesyltransferase 1	GGCGGAAGGTGATACCCAAG	CGAAACTGCGACTGGTCTGA	Liver
FGF21	Fibroblast Growth Factor 21	AGACGGGAGACTGTACGGAT	CCAAGGGCCTCAGACTGGTA	Liver, VAT and SAT

FGFR4	Fibroblast Growth Factor Receptor 4	CGGCCTTTCCTACCAGTCAG	AGCAGAAGCACAAGCAAAGC	Liver
FOXA1	Hepatocyte Nuclear Factor 3 Alpha	ATGGAAGGGCATGAGAGCAG	GCTCAGACCCGAGTTCATGT	Liver
FOXO1	Forkhead Box Protein O1	ACCAAAGCTTCCCACACAGT	TGCTTCTCTCAGTTCCTGCTG	Liver
GAPDH	Glyceraldehyde 3-phosphate Dehydrogenase, Reference gene	ACACTCACTCTTCTACCTTTG	CAAATTCATTGTCGTACCAG	Liver
GCG	Glucagon	ATCTTGCCACCCGAGACTTT	TGAACACGATCTTGATTAGTGACAT	Liver
GCK	Glucokinase (Hexokinase 4)	TCCAGGCTGCAGACAAGAAG	CCTGATGGCCTCACAGACAG	Liver
GCKR	Glucokinase Regulator	TCCAGGCTGCAGACAAGAAG	CCTGATGGCCTCACAGACAG	Liver
GCLM	Glutamate-Cysteine Ligase Modifier Subunit	TCTTGCCTCTTGCTGTGTGA	CAGGGATGCTTTCCTGAAGA	VAT and SAT
GHR	Growth Hormone Receptor	TTTCTGGGAGTGAAGCCACA	TCTGTAAACCGTGACGGACC	Liver
GLP1R	Glucagon-like Peptide 1 Receptor	ATGTGCAAGACGGACACCAA	AGCAGTACAAGATCGCCACC	Liver
GLUT2	Solute Carrier Family 2 Member 2 (SLC2A2)	TTGGTGTGACCAATGCACCT	TTTTCGGTCATCCAGCGGAA	Liver
GLUT4	Glucose Transporter Type 4	TAAGACAAGATGCCGTCGGG	GAGAAGACGGCGAGGACAAG	VAT and SAT
GNAS	GNAS Complex Locus	TTTGCTCGCTACACTACTCCT	ACGGCGGATGTTCTCAGTGT	VAT and SAT
GNMT	Glycine N-methyltransferase	GCTTCGATGCTGTCATCTGC	GTGCTGAGGATGTGGTCGTA	Liver
GPC4	Glypican 4	GGACGACTGCTGGAATGGAA	TGGACCTCTGGATTGTTGCC	VAT and SAT
GPX1	Glutathione Peroxidase 1	CACCCAGATGAATGAGCTGC	GTCGGACGTACTTGAGGCAA	Liver
GRB10	Growth Factor Receptor Bound Protein 10	GGGCTTTTTCTCCTCCGTGA	AAGAAGGTCTGCCCGTCATC	Liver, VAT and SAT
HLCS	Holocarboxylase Synthetase	TCCTTAGGGAGATCCTGACCA	TTTCCCCAGCCACTGCATAA	VAT and SAT
HMGCR	3-hydroxy-3-methylglutaryl-CoA Reductase	GACTCCGTTGACTGGAGACG	AAAGAGGCCATGCATTCGGA	Liver
HPRT1	Hypoxanthine Phosphoribosyltransferase 1, Reference Gene	GGACTTGAATCATGTTTGTG	CAGATGTTTCCAAACTCAAC	VAT and SAT
ICAM-1	Intercellular Adhesion Molecule 1	CCACGGTGCCAATTTCTCTT	TTCCACAAGCTCAGGGGTAG	VAT and SAT
IDE	Insulin Degrading Enzyme	AGCTATGACCTCCAAAATACCATCT	AGCAAAATTGGCTGCTTGTCA	Liver
IDS	Iduronate 2-Sulfatase	CGTCCAAGCCCTAAACCTCA	CTGCGTGTCCACATACGAGA	VAT and SAT
IGF1	Insulin-like Growth Factor I	GACATGCCCAAGGCTCAGAA	GAGCAAAGGATCCTGCCAGT	Liver
IGF2	Insulin-like Growth Factor II	CTGTGTTCCGGGAGAAAGTC	ACCTGGTCCAACTCCTGCT	Liver
IGFBP2	Insulin-like Growth Factor-binding Protein 2	CTGTGTTCCGGGAGAAAGTC	ACCTGGTCCAACTCCTGCT	Liver
IL18	Interleukin-18	CTGCTGAACCGGAAGACAAT	TCCGATTCCAGGTCTTCATC	Liver
IL1B	Interleukin 1 Beta	GGGTGAGTGTGCCACCATTA	TCATTGAACAATCTGATCATCCTTC	Liver
IL6	Interleukin 6	TGGGTTCAATCAGGAGACCT	CAGCCTCGACATTTCCCTTA	Liver, VAT and SAT
INSIG1	Insulin-induced Gene 1 Protein	GCCTTGTTGCGGTTTGGTTT	AGAAGGGGTCTCTGCAAAGTG	Liver
INSIG2	Insulin-induced Gene 2 Protein	CAGTGTAATGCGGTGCGTAG	GCAGCCAGTGTGAGAGACAA	Liver
INSR	Insulin Receptor	GAAAGGGGGCAAGGGTCTAC	GGCCTTGGTAAGGCTGTTCT	Liver
IRS1	Insulin Receptor Substrate 1	CGAGACCTTCTCCTCGACAC	GTTCTCGAAGGAGGCAGAGC	Liver, VAT and SAT
IRS2	Insulin Receptor Substrate 2	GGTCTTTGTACATTTTGCCTGA	ACTTCGCATCTCCATCTCCA	Liver
IRX3	Iroquois Homeobox 3	AGTGCCTTGGAAGTGGAGAA	AGCCGATAAGACCAGAGCAG	VAT and SAT
ISLR	Immunoglobulin Superfamily Containing Leucine Rich Repeat	CCTCATCTTCCCTTGCCCTG	GCAAACACAGCTCCTGCATC	VAT and SAT
JAG1	Jagged 1	TGTTAGCAAACGTGACGGGA	GGGGCACCAGGAAATCTGTT	Liver, VAT and SAT
KLB	Beta-Klotho	TGGTTCACAGACAGTCACGT	TGCCATTCAAAGCCATCCAG	Liver, VAT and SAT
LCAT	Lecithin-cholesterol Acyltransferase	TTGTCTACAACCGCACCTCT	CAGTACACACACCTGCCAAC	Liver
LCN2 (NGAL)	Lipocalin 2 (Neutrophil Gelatinase-Associated Lipocalin)	ATCATCTTCCCCGTCGCAATC	GGGTGGCATGATGGGCAAAG	VAT and SAT
LDAH	L-lactate Dehydrogenase A Chain	ATGGACTCCGTGGTCAAGTC	GCCTATAGAATGGCCGATGA	Liver (MxPro)
LDLR	Low Density Lipoprotein Receptor	GCAGTGCGACAGGGAATATG	ACACTTGAACTTGTTGGGCC	Liver, VAT and SAT
LEP	Leptin	TGACACCAAAACCCTCATCA	ATGAAGTCCAAACCGGTGAC	Liver, VAT and SAT
LEPR	Leptin Receptor	GTATCATAGGAGTAGCCTCT	ATGCATTCATAAAAACCATC	VAT and SAT
LEPR_01	Leptin Receptor 01	GATGTTCCAAACCCCAAGAA	GAAATGGTTTCAGGCTCCAA	Liver
LEPR_02	Leptin Receptor 02	GATGTTCCAAACCCCAAGAA	TTGTGTCCCTGGGTACTTCA	Liver
LEPR_03	Leptin Receptor 03	GATGTTCCAAACCCCAAGAA	TGGCAAGTTGGTAGATTGGA	Liver

LITAF	Lipopolysaccharide Induced TNF Factor	GACGGAAAGGGCATGAATCC	ATCTTGTTGCAGGAGGGACA	VAT and SAT
LPIN1	Lipin 1	AACTCTTTCTAGTGAGATCCCTCC	GCCGGGAAACTGAAGGACTG	Liver
LPL	Lipoprotein Lipase	CCCTGGCTTTGCTATTGAGA	ACTTGTCGTGGCATTTCACA	Liver, VAT and SAT
LSS	Lanosterol Synthase (2,3-Oxidosqualene-Lanosterol Cyclase)	TTGGCCGTCCTGAATGTGTA	CACCAGAGCTTGGAAGGGTG	VAT and SAT
MBOAT7	Membrane Bound O-acyltransferase Domain Containing 7	GGTGGCTGGCACAGTACAT	TCAGGTAGTAGCCAGGGTGC	Liver
MC4R	Melanocortin 4 Receptor	GGGTGTCATAAGCCTGTTGG	CACAGCCAGGCTACAGATGA	VAT and SAT
MCM5	Minichromosome Maintenance Complex Component 5	GAGGCTCTTCCAAGTGTCCA	CTCAGCAACTCCTGGTCCTC	Liver
MGMT	O-6-Methylguanine-DNA Methyltransferase	TTACCAGCAGTTAGCAGCCC	CTGCTGAGGACCACTCTGTG	VAT and SAT
MKL1	Megakaryoblastic Leukemia (Translocation) 1	CTATGAGGAAGCCGTGAGCC	GGGACTTCTCCTTCCCTGGT	VAT and SAT
MMP2	Matrix Metallopeptidase 2	TCCCCAAGCTCATTGCAGAC	CTTCAGGTAATACGCGCCCT	Liver
MMP9	Matrix Metalloproteinase-9	CGGGAGACCTACGAACCAAT	TCCAGGGACTGCTTTCTGTC	Liver
MOCOS	Molybdenum Cofactor Sulfurase	TCATCTCACGTTTCCGTGCC	ATCTGTGACAAGGCCCCAAA	VAT and SAT
MTOR	Mechanistic Target of Rapamycin Kinase	CAGCACATGCAGCACTTTGT	TTCAGCTGCCACTCTCCAAG	VAT and SAT
MTTP	Microsomal Triglyceride Transfer Protein Large Subunit	TCCCATTGCTCCTGAAGTACG	ACGCACAGTCTTTTCGTGAAC	Liver
MYC	Myc Proto-Oncogene, bHLH Transcription Factor	CGAACCCTTGGCTCTCCAC	CCGATTCCGACCTTTTGGCA	VAT and SAT
NCOR2	Nuclear Receptor Corepressor 2	CTCAGAGGGAGACTGCAACC	GGGTTGTAGGGGAAAGGTGT	VAT and SAT
NEGR1	Neuronal Growth Regulator 1	CGGCGGTGCTTAGGTGTTAT	CTCGAGGATCCACTGACCAC	VAT and SAT
NFKBIA	NFKB Inhibitor Alpha	GAGGATGAGCTGCCCTATGAC	CCATGGTCTTTTAGACACTTTCC	VAT and SAT
NPEPL1	Aminopeptidase-like 1	GTTCTTCCTGGTGGGACAGG	TTCATCTCATTGCAGGGCGT	VAT and SAT
NR1D1	Nuclear Receptor Subfamily 1 Group D Member 1	CCAGTTTGTGTCAAGGTCCA	GCCAATGTAGGTGATGACGC	Liver
NR1I2 (PXR)	Nuclear Receptor Subfamily 1 Group I Member 2	GAGAACAGATCGGGGCTCAG	TGTGAAGGTGGTGTCAAAGG	Liver
NR3C1	Nuclear Receptor Subfamily 3 Group C Member 1 (Glucocorticoid Receptor)	AGTCAGAACTGGCAACGCT	CAGCTAACATCTCTGGGAATTCAA	Liver, VAT and SAT
OSBPL10	Oxysterol Binding Protein Like 10	CCCGAAGAGCCAAGAGTCAG	CAATGGCATGCACGAGGTTC	VAT and SAT
PCSK9	Proprotein Convertase Subtilisin/Kexin Type 9	TGCTTCACGTCACAGAGTGG	GCAGAGAAACGGATCAGCCT	Liver
PEG10	Paternally Expressed 10	AGTCCTCGCGTGGTGAGTAT	TTCACTCCTGTGGGGATGGA	VAT and SAT
PELI2	Pellino E3 Ubiquitin Protein Ligase Family Member 2	GTGAAATACGGGGAGCTGGT	CTGGGTTTGACTCCATTGGC	VAT and SAT
PEMT	Phosphatidylethanolamine N-methyltransferase	GGACACCAGGCAGACCTC	TGGCGACCACATTCCAGAAG	Liver
PN-1 (SERPINE2)	Proteinase Nexin 1 (Serpin Peptidase Inhibitor, Clade E, Member 2)	GAAGTCAGCGAAGACGGAAC	GCCCCATGAATAAAACAGCA	VAT and SAT
PNPLA2	Patatin like Phospholipase Domain Containing 2	CAACGCCAAGCACATCTACG	GCCTCCTTCGACACCTCGAT	VAT and SAT
PNPLA3	Patatin-like Phospholipase Domain Containing 3	CAGACATGCCTGACGACATC	CCCCTTGTGTTCTGGTTTGT	Liver
PON1	Paraoxonase 1	GATGGCCAGTGGATTTGATT	TTATGAGCCATCGATTCAGC	VAT and SAT
PPARA	Peroxisome Proliferator Activated Receptor Alpha	TTTCCCTCTTTGTGGCTGCT	GGGGTGGTTGGTCTGCAAG	Liver
PPARD	Peroxisome Proliferator Activated Receptor Delta	TCGCGTGGTGTTTTGGGTAT	CTGGGTCTGAGTGCAGATGG	VAT and SAT
PPARG	Peroxisome Proliferator Activated Receptor Gamma isoforms 1 & 2	GCCGTGTCTGTGGGGATAAA	CCGACAGTTAAGATCGGACCT	Liver , VAT and SAT
PPARG1	Peroxisome Proliferator Activated Receptor Gamma isoform 1	GGCCACCACCGCAGATTT	CATGGTCACCTCGCTAAAAGA	Liver , VAT and SAT
PPARG2	Peroxisome Proliferator Activated Receptor Gamma isoform 2	ATGGGTGAAACTCTGGGAGA	TTTGCAGACAGCGTGTCGAA	Liver , VAT and SAT
PPARGC1A	Peroxisome Proliferator Activated Receptor Gamma Coactivator 1-alpha	TGTGGACGCAAGCAATTTTTCA	ATGTTACCTGCGCAAGCTTCT	Liver
PRKG1	Protein Kinase cGMP-Dependent 1	GAGGGCTTTAATTGGGAAGG	TGTCCCATCCTGAGTTGTCA	VAT and SAT
RBP4	Retinol-binding Protein 4	CAGTACCGGCTGATCACTCA	TGACACCCTCCATGTTGCTA	Liver
RDH5	Retinol Dehydrogenase 5	GAGGCTACTGCGTCTCCAAG	CCAGCGGGTCTTTATGATGT	VAT and SAT
RHOQ	Ras Homolog Family Member Q	CAAGCAGTACCTCCTGGGAC	GGCACCCACTCCTCTTTCAC	VAT and SAT
RNF10	Ring Finger Protein 10	ACACGCCAGCTACTTCAGAC	TCACTTGGTGTGGACGACTG	VAT and SAT
RORA	RAR Related Orphan Receptor A	CACCGCGGCTTAAATGATGT	AGGAGTAGGTGGCATTGCTT	Liver, VAT and SAT
RPL4	60S Ribosomal Protein L4, Reference gene	CAAGAGTAACTACAACCTTC	GAACTCTACGATGAATCTTC	Liver
RPLP2	Ribosomal Protein Lateral Stalk Subunit P2	GACGACGATCGGCTCAACAA	CCCCGCCAGAAGGTACACTG	VAT and SAT
RPS29	Ribosomal Protein S29	AGTCTGTCCTTTTGCCTCGT	CACTGGCGGCACATATTGAG	VAT and SAT
SAA	Serum Amyloid A1	TGGAGAGCCTACTCGGACAT	CCTTTGGGCAGCATCATAGT	VAT and SAT

SCAP	SREBF chaperone	GCCTGACACCTACCCTCAAC	GGTCGAGACCACTGACTTGG	Liver
SCARB1.v1	Scavenger Receptor Class B Member 1, Splice Variant 1	TCAAAGGATAAGGAGGCCATT	TTGCTTCTTGCAGTACAGTT	Liver
SCD	Stearoyl-CoA Desaturase	TTGATCCCCACCTGCAAGAT	CGTGTTGGCAATGATCAGGA	Liver
SEPT9	Septin 9	GGTGCCCCAGGAGAAGAGA	GATCCCCACGTAGCCGAAG	VAT and SAT
SIM1	Single-minded Family bHLH Transcription Factor 1	CTCGGCTATCACCTCGCAG	CAGGGGACTGGTCCTACTCG	VAT and SAT
SMAD6	SMAD Family Member 6	TGCAACCCCTACCACTTCAG	TGGCCTCCGTTTCAGTGTAA	VAT and SAT
SMPDL3A	Sphingomyelin Phosphodiesterase Acid-Like 3A	ACCAACAATCCTGGCATCAG	TGTCATAGGTCTGGGTCAGGT	VAT and SAT
SOD1	Superoxide dismutase 1, Cytosolic	GCAGGGCACCATCTACTTCGAG	GGAATCCATGATCACCTTCAGCC	Liver
SOD2	Superoxide Fismutase 2, Mitochondrial	GGCCTACGTGAACAACCTGA	TGATTGATGTGGCCTCCACC	Liver
SP1	Specificity Protein 1 Transcription Factor	AGTTGGTGGCAATAATGGGG	GGGTGACTCAATTCTGCTGC	VAT and SAT
SPP1	Secreted Phosphoprotein 1	GCGGGGATAGTGTGGTCTAT	TCCGTCTCCTCACTTTCCAC	Liver, VAT and SAT
SREBF2	Sterol Regulatory Element Binding Transcription Factor 2	CACCGCCCTCGCGTCT	CCCACTTGATTGCTGACAAACT	Liver
SREBP1	Sterol Regulatory Element Binding Protein 1	GGCCGAGCCATGCGAGCT	TTGTTGATGAGCTGAAGCATGT	Liver, VAT and SAT
STAT3	Signal Transducer and Activator of Transcription 3	ACCAACGACCTGCAGCAATA	CCATGTCGAAGGTGAGGGAC	Liver
STIM1	Stromal Interaction Molecule 1	TACCACGACCCAACAGTGAA	TCATCCACGGTCCAGTTGTA	VAT and SAT
SYNE1	Spectrin Repeat Containing Nuclear Envelope Protein 1	AACTGGACACCTCGGGATCT	GAGGAATCGGAGCCACCTTT	VAT and SAT
TBC1D16	TBC1 Domain Family Member 16	CACCTTCTGGTGCTTTGTGG	TCCTCACCTAGCGAGACCAG	VAT and SAT
TBCD	Tubulin Folding Cofactor D	GTGTACGAGATGGTGCTGACC	GGCTTGGAGACCAGCTGAGG	VAT and SAT
TBP	TATA-Box Binding Protein, Reference Gene	AACAGTTCAGTAGTTATGAGCCAGA	AGATGTTCTCAAACGCTTCG	Liver, VAT and SAT
TCF7L2	Transcription Factor 7-like 2	CCAAGAGGCAAGATGGAGGG	TAATGTGTGCTCCCGGACTG	Liver
TECPR2	Tectonin Beta-Propeller Repeat Containing 2	GGCTGCTCAAGTCAGATCAGT	CCGCCTTTGTAGTCCAGACA	VAT and SAT
TGFB1	Transforming Growth Factor Beta 1	GCAAGGTCCTGGCTCTGTA	TAGTACACGATGGGCAGTGG	VAT and SAT
TGFB1	Transforming Growth Factor Beta 1	GAGCCAGAGGCGGACTACTA	TCCGACGTGTTGAACAGCATA	Liver (MxPro)
TGFB3	Transforming Growth Factor Beta 3	AGTGCAGACACAACCCCACAG	GGTCCTCCCGACGTAGTACA	VAT and SAT
TIMP1	Metalloproteinase Inhibitor 1	GAGATCTATGCTGCTGGCTGT	GAGCTGGTCTGTCCACAAGC	Liver
TLR4	Toll Like Receptor 4	AGAACTGCAGGTGCTGGATT	TGGATAGGATTTCCCGTCAG	Liver, VAT and SAT
TM6SF2	Transmembrane 6 Superfamily Member 2	GCCTCATCTTTCCTGGCTGT	GAGGCACCCATGTGTGAGAA	Liver
TNF	Tumor Necrosis Factor	CCCCCAGAAGGAAGAGTTTC	CGGGCTTATCTGAGGTTTGA	Liver, VAT and SAT
TNFRSF1A	TNF Receptor Superfamily Member 1A	GAGAGTTTGTGTCCCCAAGGAA	AGTGAAGGTGCCATTGTCGC	Liver
TNS3	Tensin 3	CCCGTGAGCAGTGTCATCTT	CGGGCCACAAATCCAAAGAC	VAT and SAT
USF1	Upstream Transcription Factor 1	AGAGAGGGAGAACTCGGTCC	TGTTTTCTGCTGCCCCTTCA	VAT and SAT
YWHAZ	Tyrosine 3-Monooxygenase/Tryptophan 5-Monooxygenase Activation Protein Zeta, Reference Gene	TGATGATAAGAAAGGGATTGTG	GTTCAGCAATGGCTTCATCA	VAT and SAT

reference genes in Fluidigm, TBP used in MxPro
