# Supplementary material for: The expression signatures in liver and adipose tissue from obese Göttingen Minipigs reveal a predisposition for healthy fat accumulation
Source: Nutr Diabetes. 2020 Mar 23;10:9. doi: 10.1038/s41387-020-0112-y (PMC7090036; doi:10.1038/s41387-020-0112-y)
Supplement: Supplementary file 3 — S2 Table [file 41387_2020_112_MOESM3_ESM.pdf]

PCR data from liver.

Samples	ABCC2	ACACA	ACTA2	ACTB	ADIPOQ	ADIPOR1	AGT	APOA1	APOA4	APOB	APOC3	CCL2	CCL5	CD36	CD68	COL1A1	CTGF	CTP1A	DGAT2	FABP4	FDFT1	FGF21	FGFR4	FOXA1	FOXO1	GAPDH	GCKR
319427.1	8.56	10.70	10.34	7.25	10.66	10.58	6.97	5.74	13.78	3.93	3.38	12.14	11.99	12.46	11.07	10.32	13.07	9.76	8.12	15.99	6.36	17.70	9.67	11.49	9.30	7.32	9.12
319427.2	9.33	11.64	11.40	7.75	11.36	11.24	7.75	6.15	14.28	4.54	3.95	12.81	12.75	12.65	11.50	11.34	13.86	10.52	8.80	17.42	7.00	18.70	10.24	12.29	10.13	8.03	9.81
319778.1	9.80	12.28	9.83	8.43	11.42	11.23	7.70	5.73	10.72	5.12	3.99	13.39	13.41	13.21	12.47	12.00	12.18	10.81	9.38	14.79	8.18	12.18	11.10	13.23	10.15	9.41	9.92
319778.2	11.22	13.61	10.94	9.41	12.77	12.65	8.89	6.84	12.15	6.16	4.97	14.57	14.43	13.56	13.05	13.10	13.09	12.03	10.73	16.10	9.46	13.50	12.34	14.49	11.49	10.60	11.02
319860.1	9.76	12.48	11.91	9.25	11.66	11.62	7.86	5.75	14.91	5.22	3.77	14.97	13.01	13.80	12.07	13.07	13.49	12.20	9.03	16.63	10.94	17.94	11.57	13.28	9.93	9.95	9.62
319860.2	9.76	12.48	11.91	9.25	11.66	11.62	7.86	5.75	14.91	5.22	3.77	14.97	13.01	13.80	12.07	13.07	13.49	12.20	9.03	16.63	10.94	17.94	11.57	13.28	9.93	9.95	9.62
319922.1	9.47	12.22	10.99	7.84	10.74	10.68	6.95	5.08	11.47	4.52	3.50	12.21	12.18	12.98	11.33	12.87	12.72	10.79	8.55	15.59	8.39	12.32	10.57	13.01	9.50	9.22	9.20
319922.2	8.81	11.75	10.80	7.85	10.48	10.32	6.72	4.87	11.02	4.41	3.45	11.97	12.11	12.95	11.47	12.82	12.62	10.39	8.26	15.47	8.20	11.90	10.13	12.76	9.16	9.33	8.73
320207.1	8.93	10.61	10.43	7.59	9.95	9.87	6.22	4.58	11.99	4.34	3.31	12.07	12.09	11.75	11.31	11.21	10.19	9.89	7.81	17.27	7.80	16.10	9.48	11.77	9.04	8.18	8.18
320207.2	9.87	11.58	10.40	7.78	10.72	10.54	6.27	4.90	12.80	4.84	3.19	12.01	12.26	12.46	11.55	11.42	10.61	10.66	8.22	16.73	8.37	16.49	10.65	12.15	9.00	8.42	8.89
320340.1	9.69	12.56	12.01	7.71	11.48	11.32	7.44	5.47	9.22	5.00	4.22	11.75	13.76	10.95	11.29	11.41	12.52	9.95	9.10	16.07	8.47	14.20	11.72	13.12	10.95	8.29	9.62
320340.2	8.65	11.69	11.07	6.95	10.64	10.45	6.62	4.83	8.25	4.26	3.54	10.86	12.80	10.78	10.41	10.49	11.65	9.05	8.36	15.00	7.51	13.30	10.79	12.34	10.00	7.48	8.84
320411.1	10.70	13.50	11.29	9.08	12.08	12.10	8.70	6.36	10.61	5.76	4.21	13.70	13.03	12.26	12.47	12.76	13.49	11.02	9.69	16.92	9.59	14.27	11.77	14.08	10.63	9.54	10.04
320411.2	11.60	14.74	11.79	9.48	12.90	12.91	9.32	6.96	11.43	6.71	4.44	14.13	13.50	12.71	12.74	13.22	14.40	11.79	10.41	17.08	10.29	14.97	13.09	14.78	11.26	10.19	11.07
221497.1	8.34	10.88	8.84	6.34	9.82	9.73	6.87	4.89	10.12	4.57	3.62	10.42	10.58	9.47	7.79	7.71	8.85	10.17	8.54	13.24	9.66	14.98	9.71	10.33	9.04	7.86	8.85
221497.2	8.34	10.88	8.84	6.34	9.82	9.73	6.87	4.89	10.12	4.57	3.62	10.42	10.58	9.47	7.79	7.71	8.85	10.17	8.54	13.24	9.66	14.98	9.71	10.33	9.04	7.86	8.85
221510.1	8.18	11.72	9.45	6.58	9.95	9.90	7.25	4.41	9.57	4.22	3.43	11.80	10.01	9.53	7.32	8.97	12.05	9.17	8.65	13.68	9.30	14.75	9.72	11.52	9.20	8.47	8.81
221510.2	9.03	12.93	10.74	7.82	11.34	11.28	8.68	5.49	11.04	5.20	4.47	13.31	11.70	10.70	8.95	10.48	13.53	10.35	9.88	14.78	10.62	15.94	10.93	12.51	10.66	9.63	10.22
221538.1	8.63	12.24	8.95	7.08	10.62	10.54	7.58	5.11	7.81	4.70	3.83	10.88	10.77	10.99	8.86	9.52	11.65	8.43	9.45	14.37	10.79	11.11	10.89	12.67	9.77	8.08	9.58
221538.2	9.83	13.34	9.12	7.73	10.98	10.92	8.29	5.80	8.63	5.72	3.90	11.18	11.22	10.33	8.35	9.63	12.06	9.68	10.08	13.88	11.24	11.46	12.42	13.88	9.89	8.43	10.15
319035.1	8.68	12.72	11.76	7.94	11.13	10.94	7.38	4.99	11.96	4.93	3.95	13.48	12.30	10.28	8.83	12.25	12.56	11.40	9.12	15.00	10.43	19.24	10.32	12.64	10.11	8.66	9.29
319035.2	8.94	12.70	10.96	8.22	10.74	10.57	7.01	4.99	11.33	4.91	3.32	12.49	11.73	9.06	7.47	11.27	11.74	11.75	8.59	14.15	10.13	18.43	11.00	12.86	9.41	8.03	9.03
319239.1	9.14	12.35	9.52	7.31	10.56	10.51	8.20	5.19	9.89	4.93	4.16	11.55	11.13	9.11	8.29	9.63	12.74	10.31	10.03	13.85	10.66	12.26	10.68	12.06	9.78	9.18	9.46
319239.2	8.54	11.80	9.12	6.82	9.76	9.94	7.46	4.82	9.74	4.19	3.56	10.64	10.37	8.11	6.99	8.98	11.85	9.72	9.18	13.15	10.36	11.52	10.19	11.37	9.07	8.60	9.05
319389.1	8.86	12.14	10.63	7.70	11.15	11.15	8.14	5.30	9.10	4.94	3.90	13.17	12.25	11.75	9.82	9.62	13.69	9.59	8.53	14.74	8.37	13.40	11.14	13.36	10.25	7.92	9.49
319389.2	9.86	13.28	11.48	8.80	12.18	11.95	9.15	6.17	10.06	6.21	4.28	13.69	13.04	11.43	10.09	10.02	14.30	10.57	9.38	15.12	9.21	14.15	13.10	14.86	10.77	8.61	10.37
319798.1	8.60	11.78	7.55	6.45	10.19	10.10	6.95	5.20	9.08	4.58	3.76	11.08	10.68	10.75	8.70	8.20	12.17	10.00	8.77	13.08	11.06	13.95	9.96	11.31	9.11	8.04	9.27
319798.2	8.60	11.78	7.55	6.45	10.19	10.10	6.95	5.20	9.08	4.58	3.76	11.08	10.68	10.75	8.70	8.20	12.17	10.00	8.77	13.08	11.06	13.95	9.96	11.31	9.11	8.04	9.27
319799.1	8.99	11.28	10.27	6.60	10.08	9.95	7.27	5.13	9.47	4.65	3.99	10.96	10.69	9.25	8.14	8.02	10.67	9.93	9.50	13.70	11.15	11.21	9.72	10.98	9.05	7.91	9.14
319799.2	10.12	12.39	11.01	7.40	10.87	10.71	8.08	5.81	10.71	5.38	4.62	11.79	11.55	9.72	8.43	8.62	11.51	11.13	10.27	14.43	12.02	12.01	10.83	11.91	9.70	8.44	10.24
319875.1	9.50	12.41	10.67	7.94	11.35	11.26	7.90	5.59	9.76	4.90	3.67	14.83	12.48	11.74	9.98	11.73	12.84	10.72	8.70	14.48	11.54	14.43	10.99	13.39	9.96	8.85	9.93
319875.2	11.02	13.94	12.11	9.45	12.86	12.73	9.37	6.90	11.33	6.34	4.90	16.42	13.93	13.15	11.40	13.02	14.47	12.17	10.14	15.92	13.03	15.73	12.57	14.81	11.12	10.22	11.44
320241.1	8.55	12.43	9.47	7.27	10.89	10.82	7.29	5.30	12.04	4.70	3.78	13.22	11.70	9.66	8.60	10.78	12.20	10.83	8.56	13.31	9.50	13.20	10.51	12.60	9.72	9.17	9.32
320241.2	8.54	12.59	9.12	7.41	10.83	10.77	7.21	5.18	12.19	4.82	3.75	13.18	11.78	10.20	8.59	10.79	12.46	11.03	8.02	13.38	9.70	13.36	10.71	12.78	9.76	9.15	9.39
320316.1	8.79	11.69	8.69	6.69	10.30	10.22	6.98	4.95	9.60	4.74	3.55	10.72	11.19	9.62	8.81	7.13	10.10	9.66	8.34	12.34	11.09	12.61	9.92	11.61	8.76	8.62	8.85
320316.2	8.76	11.85	8.52	6.64	10.35	10.26	7.00	5.02	9.62	4.83	3.51	10.83	11.15	9.76	8.76	7.21	10.09	9.72	8.37	12.49	11.17	12.66	10.18	11.58	8.82	8.56	8.86
320317.1	8.21	11.64	9.95	6.95	10.30	10.16	6.35	4.73	7.91	4.02	3.24	8.93	10.80	9.57	8.65	11.09	11.31	8.67	7.49	14.72	9.23	14.30	10.30	11.98	9.27	8.55	9.19
320317.2	8.93	12.54	10.50	7.70	11.00	10.91	7.12	5.27	8.51	4.62	3.74	9.66	11.51	9.98	9.37	11.72	12.24	9.32	8.47	15.53	10.10	14.89	11.13	12.78	9.94	9.33	9.81
320351.1	8.66	11.18	10.89	7.43	10.36	10.22	6.66	4.86	9.85	4.62	3.31	14.41	11.56	9.56	8.24	12.57	10.38	10.02	8.22	15.67	11.30	16.01	10.06	12.40	8.31	8.00	8.74
320351.2	8.51	11.07	10.67	7.12	10.31	10.12	6.46	4.69	9.51	4.43	3.18	14.18	11.39	10.04	8.24	12.47	10.35	9.74	8.01	15.75	11.18	15.87	10.02	12.13	8.26	7.91	8.68
320440.1	10.20	13.46	11.60	8.43	11.91	11.76	8.63	7.04	10.42	5.90	5.02	13.47	12.88	9.94	9.15	12.00	11.83	11.02	10.31	15.28	11.98	14.51	11.92	13.15	10.70	9.53	11.04
320440.2	11.20	14.62	11.89	8.96	12.88	12.63	9.22	7.80	11.30	6.74	5.43	13.92	13.27	10.31	9.56	12.55	12.73	12.14	11.02	15.70	12.51	15.31	12.89	13.75	11.12	10.07	12.07
221516.1	7.87	11.62	8.75	6.73	10.29	10.30	8.05	5.10	8.28	4.97	3.57	10.36	10.89	8.82	7.96	6.98	8.31	9.39	8.46	10.37	10.95	11.58	10.67	11.50	9.24	7.15	9.40
221516.2	7.68	11.79	8.85	7.01	10.90	10.86	8.23	5.49	8.46	4.44	3.98	11.04	11.56	9.48	8.99	7.44	8.92	9.28	9.18	10.94	10.90	12.01	10.51	11.71	9.63	7.90	9.82
319021.1	8.12	11.56	9.52	7.37	10.74	10.58	8.54	5.22	7.93	4.73	4.15	12.13	11.85	9.91	8.91	8.38	10.31	9.70	9.06	10.49	11.36	15.58	10.58	11.77	10.07	9.98	9.91
319021.2	9.27	12.98	10.21	8.83	11.71	11.45	9.58	6.36	8.77	5.97	4.13	12.69	12.22	10.45	9.05	8.68	11.02	11.14	9.93	10.91	12.52	16.52	13.06	13.79	10.29	9.50	10.96
319537.1	10.95	13.43	14.19	9.87	12.85	12.73	9.94	7.19	10.01	6.31	5.25	16.17	14.20	11.52	10.66	13.56	13.93	11.75	11.18	16.20	14.42	15.75	12.84	13.80	11.35	10.19	11.46
319537.2	9.56	12.33	12.76	8.81	11.72	11.64	8.99	5.98	8.67	5.30	4.39	14.91	12.58	10.26	9.33	12.64	12.49	10.65	10.07	14.62	13.24	14.51	11.57	13.05	10.32	9.12	10.18
319677.1	8.29	11.01	10.16	6.97	10.33	10.29	7.71	5.36	10.72	4.56	3.95	12.41	10.88	9.33	7.75	9.25	11.10	10.48	8.92	13.22	10.71	16.43	10.50	11.47	9.53	8.56	9.99
319677.2	8.29	11.01	10.16	6.97	10.33	10.29	7.71	5.36	10.72	4.56	3.95	12.41	10.88	9.33	7.75	9.25	11.10	10.48	8.92	13.22	10.71	16.43	10.50	11.47	9.53	8.56	9.99
319703.1	9.54	11.92	12.65	8.55	11.13	11.02	8.80	6.18	8.71	5.14	3.99	14.19	12.35	10.03	8.66	11.50	11.20	9.87	9.16	12.38	12.96	17.64	11.50	13.16	10.55	9.47	10.16
319703.2	9.28	11.73	12.19	8.27	10.86	10.71	8.24	5.93	8.67	4.92	3.81	13.92	11.98	9.95	8.41	11.22	11.07	9.63	8.88	12.19	12.80	17.54	11.22	12.81	10.24	8.64	9.95
319876.1	8.87	11.45	11.04	8.01	10.91	10.71	8.35	5.45	7.91	4.59	3.62	14.38	12.15	9.17	8.90	11.86	11.52	9.70	8.51	11.69	11.38	15.52	10.90	12.09	9.92	8.93	9.72
319876.2	8.87	11.45	11.04	8.01	10.91	10.71	8.35	5.45	7.91	4.59	3.62	14.38	12.15	9.17	8.90	11.86	11.52	9.70	8.51	11.69	11.38	15.52	10.90	12.09	9.92	8.93	9.72
320335.1	8.90	11.96	10.05	7.08	10.08	9.97	7.11	5.34	8.05	4.41	3.44	11.70	10.99	7.88	7.79	8.42	9.85	9.37	8.77	9.54	12.12	14.52	10.63	11.32	9.42	8.51	9.26
320335.2	9.07	12.40	10.41	7.41	10.63	10.48	7.53	5.69	8.55	4.69	3.85	12.11	11.44	8.09	8.03	8.76	10.24	9.71	9.22	9.90	12.47	14.80	10.97	11.65	9.78	9.10	9.61
320462.1	10.54	13.24	11.54	8.68	12.56	12.44	10.09	7.49	9.40	6.04	5.27	14.44	12.86	9.36	8.97	12.43	12.88	10.66	10.44	14.54	14.20	17.42	13.04	13.71	11.56	10.04	11.77
320462.2	11.72	14.48	13.15	9.87	13.96	13.86	11.39	8.77	10.83	7.21	6.60	16.59	14.52	10.83	10.36	13.85	14.04	11.81	11.43	15.88	15.74	18.76	14.21	14.83	12.96	11.44	13.13

raw qPCR data efficiency corrected

ref genes

GHR	GLUT2	GNMT	GRB10	HMGCR	IDE	IGF1	IGFBP2	IL18	IL1B	IL6	INSIG1	INSIG2	INSR	IRS2	JAG1	KLB	LCAT	LDLR	LEPR_01	LEPR_03	LPIN1	LPL	MBOAT7	MCM5	MMP2	MMP9	MTTP
8.50	8.68	8.22	11.47	11.00	10.91	11.72	7.28	9.83	13.80	18.38	6.48	10.79	11.11	6.63	11.80	6.22	15.90	10.15	17.07	11.01	10.35	20.56	11.73	16.41	13.71	16.66	7.40
9.22	9.33	9.00	12.03	11.85	11.74	12.35	7.97	10.69	14.52	18.73	7.26	11.50	11.69	7.28	12.69	6.81	16.82	10.69	17.71	11.66	10.72	19.92	12.19	17.11	14.34	17.01	8.35
10.24	9.94	7.79	12.06	14.19	12.14	13.40	7.62	11.80	13.93	18.90	8.15	9.58	11.85	8.56	12.69	9.73	15.60	11.33	16.59	10.05	11.92	19.79	12.93	17.92	14.26	17.28	8.65
11.49	11.36	8.82	13.44	15.34	13.24	14.42	8.74	12.95	14.82	20.21	9.29	10.71	13.37	9.56	13.67	10.84	16.64	12.67	17.32	11.12	13.04	20.44	14.07	19.64	15.59	17.80	9.84
9.97	10.42	8.32	12.77	18.32	12.16	16.32	8.45	11.99	15.73	20.03	11.68	11.27	12.70	8.49	12.87	12.03	17.05	14.67	16.92	10.71	12.34	20.44	13.26	18.17	15.68	18.62	8.95
9.97	10.42	8.32	12.77	18.32	12.16	16.32	8.45	11.99	15.73	20.03	11.68	11.27	12.70	8.49	12.87	12.03	17.05	14.67	16.92	10.71	12.34	20.44	13.26	18.17	15.68	18.62	8.95
9.24	9.90	7.58	11.87	15.39	11.35	12.32	6.56	11.37	14.83	18.54	7.73	9.34	11.34	7.97	11.56	10.05	14.52	11.37	16.49	10.36	10.49	19.46	12.22	16.40	14.49	17.67	8.16
8.90	9.60	7.23	11.56	15.01	10.89	12.31	6.30	11.28	14.40	18.74	7.92	8.73	10.82	7.83	11.33	9.83	13.93	10.52	16.54	10.04	10.35	20.17	11.90	16.14	13.96	17.80	7.75
8.15	9.13	6.84	10.85	14.56	10.94	15.01	6.16	11.32	12.35	17.57	6.43	8.58	9.91	7.58	11.25	8.63	13.64	10.75	16.04	10.19	9.80	19.52	11.05	15.97	13.08	17.18	7.48
9.30	9.96	7.48	11.35	15.47	11.55	15.57	6.82	11.51	12.50	17.79	6.84	9.46	11.00	7.71	11.72	9.47	14.90	11.88	17.37	10.63	10.44	20.44	11.80	16.57	13.79	16.91	8.49
10.04	10.61	8.22	11.75	14.52	11.74	14.96	9.35	11.31	11.68	15.11	7.68	9.78	11.83	8.99	13.28	9.11	16.67	11.75	16.51	11.32	12.53	17.87	12.24	15.37	13.98	14.62	8.44
9.10	9.71	7.42	10.89	13.61	10.87	14.13	8.65	10.38	10.73	14.06	7.08	8.90	10.90	7.98	12.63	8.31	15.74	10.91	15.49	10.51	11.78	16.79	11.37	14.23	13.04	13.81	7.60
10.09	9.63	7.43	12.54	15.66	12.67	12.91	8.44	12.21	15.84	19.38	8.46	10.10	12.13	8.51	12.59	10.75	15.49	13.13	17.56	11.04	11.32	19.59	13.34	18.62	15.03	16.66	9.31
10.83	10.27	8.15	13.41	16.10	13.29	13.60	9.32	12.30	16.25	19.51	9.09	10.65	12.98	8.84	13.46	11.77	16.99	14.46	18.62	11.52	12.14	19.59	14.18	18.55	15.66	17.18	10.16
9.67	10.45	7.89	10.07	16.61	10.49	11.45	7.06	10.17	14.39	14.62	8.98	8.98	11.06	8.09	10.41	12.84	15.61	12.17	16.55	10.83	12.35	9.63	11.11	14.28	11.14	12.19	8.41
9.67	10.45	7.89	10.07	16.61	10.49	11.45	7.06	10.17	14.39	14.62	8.98	8.98	11.06	8.09	10.41	12.84	15.61	12.17	16.55	10.83	12.35	9.63	11.11	14.28	11.14	12.19	8.41
8.68	8.82	8.20	10.55	17.09	10.44	11.61	7.81	9.67	15.29	16.90	7.36	8.65	10.49	6.96	11.42	11.84	15.46	12.84	15.36	10.03	10.17	9.53	11.50	14.68	12.58	14.70	8.09
9.93	9.83	9.52	11.70	18.43	11.63	12.92	8.96	11.02	16.34	18.67	8.34	9.65	11.79	8.13	12.86	12.97	16.68	13.87	16.75	10.91	11.49	10.75	12.61	16.29	13.80	16.08	9.14
10.08	10.08	8.03	11.02	17.49	11.22	11.42	7.89	10.32	14.54	16.08	9.63	8.94	11.21	8.70	11.55	14.54	15.16	11.64	16.60	10.10	11.86	11.73	12.38	15.41	12.90	15.01	8.18
10.50	10.86	8.37	11.33	18.43	11.60	11.77	9.02	10.59	14.90	16.48	9.68	9.11	12.17	8.74	12.24	15.15	15.72	13.01	16.59	10.68	12.58	12.48	13.07	15.89	13.51	15.72	8.83
9.26	10.00	8.57	11.65	18.65	11.47	12.33	8.22	11.44	16.58	19.43	8.26	10.78	11.58	8.33	12.31	13.74	15.99	14.18	17.65	11.15	12.52	10.32	12.51	16.38	14.46	9.74	8.96
9.06	9.84	7.97	11.23	18.64	11.07	11.64	8.96	10.69	15.86	18.25	8.14	10.48	11.67	7.64	11.86	13.49	16.19	14.67	17.00	10.92	12.62	10.29	12.27	15.63	14.03	9.48	8.57
10.33	9.80	8.91	10.77	17.85	11.18	15.12	7.94	9.98	16.00	16.79	9.21	9.11	11.28	8.43	11.53	12.44	15.68	13.26	15.79	10.64	12.13	9.77	12.12	15.47	13.09	12.81	8.69
10.02	9.51	8.02	10.05	17.52	10.55	14.58	6.99	9.42	15.35	16.63	8.72	8.71	10.69	7.77	11.10	12.17	14.85	12.80	15.58	10.06	11.41	9.35	10.98	14.75	12.50	12.07	8.03
8.98	9.98	9.02	11.61	14.06	11.24	11.57	9.18	11.05	15.27	18.08	8.11	10.17	12.03	8.20	12.67	9.20	16.15	11.68	15.94	10.76	11.74	12.83	12.52	15.13	13.41	13.64	8.63
9.89	11.30	9.23	12.40	15.46	12.20	12.45	10.65	11.91	15.70	18.84	7.76	10.95	13.26	8.79	13.69	10.42	17.06	13.59	16.75	11.38	12.79	13.69	13.98	16.19	14.30	14.76	9.24
9.81	9.64	8.35	10.95	17.40	10.88	10.74	6.76	10.65	14.06	16.44	9.31	8.71	11.15	7.86	11.14	12.97	14.83	13.68	15.81	10.28	11.19	10.52	11.64	15.16	12.35	11.95	8.20
9.81	9.64	8.35	10.95	17.40	10.88	10.74	6.76	10.65	14.06	16.44	9.31	8.71	11.15	7.86	11.14	12.97	14.83	13.68	15.81	10.28	11.19	10.52	11.64	15.16	12.35	11.95	8.20
10.10	9.00	8.16	10.20	17.39	10.81	10.04	6.84	9.75	14.47	16.64	8.15	8.80	10.35	7.65	10.79	13.30	14.73	13.97	16.39	10.67	10.71	9.16	11.16	14.34	11.51	11.79	8.34
11.18	9.77	9.25	11.06	18.51	11.58	10.85	7.80	10.53	14.98	17.35	8.75	9.71	11.28	8.23	11.60	14.31	16.05	15.00	17.61	11.44	11.48	10.21	12.17	15.22	12.41	12.46	9.44
9.82	10.37	7.26	12.06	19.95	11.93	12.79	7.88	11.14	15.75	19.28	10.89	9.44	11.70	8.35	12.47	15.10	15.32	15.20	16.32	10.45	11.66	13.72	12.99	16.62	14.52	16.26	8.64
11.34	12.00	8.78	13.62	21.44	13.51	14.53	9.39	12.69	17.95	20.86	12.56	11.09	13.12	9.75	13.74	16.62	16.77	16.83	17.75	12.08	12.98	15.23	14.42	18.35	16.04	17.55	10.03
8.79	10.15	7.45	11.60	18.04	10.95	14.68	8.30	10.83	12.35	17.84	8.87	8.65	10.91	7.99	11.85	12.03	14.73	14.16	16.32	10.24	10.94	11.97	12.10	16.30	13.74	15.15	8.31
9.03	10.26	7.58	11.69	18.63	10.85	15.21	8.17	11.09	12.83	18.46	8.81	9.02	11.14	8.15	11.80	12.52	14.97	14.20	16.35	10.46	11.07	12.18	12.11	16.40	13.78	15.22	8.57
9.78	9.85	7.67	10.26	17.93	11.13	11.87	6.70	10.49	13.62	14.81	8.41	9.66	11.08	7.94	10.86	13.62	14.08	13.92	16.29	10.12	11.41	11.72	11.58	14.48	11.24	12.99	8.37
9.96	9.92	7.92	10.48	17.81	11.40	11.89	6.72	10.78	13.67	14.92	8.39	10.00	11.12	7.95	10.78	14.24	14.27	13.95	17.07	10.54	11.34	12.02	11.72	14.55	11.42	12.95	8.48
8.55	9.91	6.78	11.19	17.83	10.46	11.47	7.56	9.16	8.13	15.77	9.76	7.95	10.60	7.63	11.45	11.92	15.27	13.66	14.30	8.99	11.75	13.84	11.56	15.17	13.77	14.22	7.28
9.52	10.68	7.43	11.82	18.89	11.50	12.41	8.19	10.07	8.92	16.61	10.54	8.96	11.21	8.30	12.08	12.80	15.85	14.56	15.34	10.03	12.59	14.31	12.31	16.27	14.35	14.68	8.17
8.35	9.13	7.80	11.11	18.91	10.82	9.64	7.78	10.53	13.24	18.71	7.96	9.19	10.80	8.62	11.46	14.79	15.01	15.16	16.39	10.02	10.49	12.01	11.72	15.19	14.42	14.30	8.19
8.29	9.16	7.78	11.10	18.16	10.82	9.56	7.74	10.70	13.21	18.99	8.10	9.19	10.68	8.54	11.27	14.86	14.85	14.91	16.84	10.03	10.38	11.99	11.54	15.04	14.19	13.99	8.09
11.79	10.80	10.41	12.17	19.33	12.40	16.55	9.34	11.97	16.12	17.10	10.03	10.48	12.50	9.35	13.47	15.97	16.98	15.29	17.51	11.73	12.10	11.36	13.34	17.59	15.21	14.83	9.76
12.58	11.35	11.33	13.10	19.58	13.09	16.78	10.26	12.30	16.62	18.67	10.77	11.13	13.38	9.65	14.25	17.43	18.18	16.28	17.60	12.24	12.84	11.93	14.24	18.15	16.08	15.10	10.54
10.65	10.49	7.72	10.05	17.35	11.20	15.30	6.64	9.97	14.09	15.08	8.82	8.27	10.87	8.16	10.82	12.91	15.38	12.79	15.49	10.20	12.20	9.83	11.67	15.16	10.97	12.49	8.33
10.99	10.54	8.30	10.47	17.19	11.40	15.39	6.90	10.43	14.64	15.44	10.00	8.60	10.88	8.55	11.15	12.65	15.80	12.53	14.96	10.08	12.29	9.75	12.07	15.36	11.02	12.65	8.49
10.10	10.98	7.68	10.68	17.09	11.31	16.83	7.36	10.45	15.15	16.64	8.47	9.09	10.80	8.19	11.34	13.81	17.17	12.91	15.37	9.95	12.44	10.26	11.96	15.89	12.15	12.88	8.31
11.14	11.11	7.99	11.40	18.80	12.33	17.19	8.87	10.93	15.83	17.21	8.24	9.79	12.63	8.17	12.24	15.32	18.56	15.12	16.14	10.71	13.77	11.24	13.60	16.96	13.36	14.20	8.96
13.11	11.10	9.10	13.30	19.52	13.29	14.87	9.42	12.62	17.62	20.67	11.32	11.94	13.22	10.29	14.02	15.29	18.85	15.23	16.22	11.43	14.56	12.91	14.55	17.95	16.16	15.38	10.21
11.95	9.98	7.82	11.95	19.31	12.13	13.99	8.37	11.27	16.00	19.78	10.06	10.54	12.10	9.09	12.87	14.31	17.18	14.02	14.76	9.85	13.27	11.45	13.33	16.16	15.17	14.26	8.88
9.91	9.85	8.02	10.73	17.57	10.92	14.94	7.12	9.95	14.96	18.10	10.09	8.84	11.23	8.47	11.35	12.79	16.77	13.24	15.90	10.95	12.53	10.20	11.96	15.63	13.35	11.83	8.56
9.91	9.85	8.02	10.73	17.57	10.92	14.94	7.12	9.95	14.96	18.10	10.09	8.84	11.23	8.47	11.35	12.79	16.77	13.24	15.90	10.95	12.53	10.20	11.96	15.63	13.35	11.83	8.56
9.91	10.43	7.37	11.97	18.80	11.70	12.57	8.03	11.52	16.09	19.29	8.12	10.69	11.41	8.84	12.87	16.12	16.88	13.92	15.42	9.98	11.65	11.58	12.67	16.13	14.41	15.05	8.58
9.79	9.83	7.12	11.55	18.88	11.28	12.24	8.06	11.36	15.23	19.42	7.71	10.44	10.99	8.69	12.59	16.17	16.92	13.64	15.02	9.74	11.44	11.31	12.34	15.95	14.18	15.05	8.49
10.03	9.85	6.79	11.41	18.64	11.38	12.65	7.97	10.55	15.08	18.97	8.28	9.50	11.00	8.44	12.21	12.87	16.02	12.69	13.82	9.47	11.85	11.69	12.47	16.35	14.43	13.76	7.98
10.03	9.85	6.79	11.41	18.64	11.38	12.65	7.97	10.55	15.08	18.97	8.28	9.50	11.00	8.44	12.21	12.87	16.02	12.69	13.82	9.47	11.85	11.69	12.47	16.35	14.43	13.76	7.98
9.27	9.19	6.07	10.48	17.18	10.45	11.61	6.90	9.60	13.98	16.19	7.68	8.56	10.55	7.65	11.55	15.38	16.22	13.99	14.43	9.12	11.38	10.04	11.45	14.49	12.46	10.96	7.90
9.61	9.76	6.45	10.95	17.32	10.87	11.98	7.20	10.02	14.33	16.83	7.83	8.97	10.97	7.90	11.91	15.92	16.36	14.26	14.61	9.51	11.66	10.30	11.81	14.92	12.95	11.33	8.20
11.80	11.77	10.14	13.01	19.74	12.83	14.42	8.90	11.94	17.43	20.21	9.40	11.09	12.99	9.88	13.87	16.06	18.03	15.27	15.49	10.57	13.13	11.45	13.93	17.62	15.90	16.16	9.81
13.17	12.88	11.47	14.18	21.57	14.02	15.93	10.26	13.56	18.89	21.76	10.59	12.46	14.08	11.42	14.81	17.57	19.53	16.17	16.63	11.46	14.42	12.69	15.31	18.65	17.27	17.37	10.85

NR1D1	NR1I2	NR3C1	PCSK9	PEMT	PNPLA2	PNPLA3	PPARA	PPARG	PPARGC1A	RBP4	RORA	RPL4	SCAP	SCARB1	SCD	SOD1	SOD2	SREBF1	STAT3	TBP	TCF7L2	TIMP1	TLR4	TM6SF2	TNF	TNFRSF1A
13.39	10.57	9.44	8.95	10.83	11.03	12.00	8.61	17.47	10.15	3.62	10.04	7.18	11.00	9.28	6.77	4.66	8.24	15.79	8.00	11.82	13.08	14.12	12.52	11.45	15.81	10.62
14.18	11.21	10.18	9.51	11.44	11.66	12.80	9.24	19.32	11.04	3.97	10.76	7.88	11.70	9.94	7.25	5.66	9.01	16.76	8.70	12.50	13.73	14.80	13.18	12.31	16.47	11.17
14.54	11.41	10.25	10.90	12.43	11.51	16.11	10.16	16.94	10.46	3.98	11.07	7.53	12.20	10.71	9.45	6.39	10.41	17.08	9.73	12.69	14.01	14.04	13.51	12.65	15.07	11.81
15.53	12.65	11.28	12.28	13.37	12.45	17.26	11.24	18.91	11.74	4.49	12.23	8.41	13.46	11.85	10.57	7.48	11.71	18.93	10.88	13.92	14.79	14.85	14.81	13.72	16.06	13.04
15.08	11.34	10.47	13.42	12.25	11.99	15.96	10.30	21.67	11.03	3.91	10.72	7.37	12.59	10.33	7.54	6.62	10.87	14.91	10.64	12.61	14.46	14.60	14.42	12.67	17.30	12.21
15.08	11.34	10.47	13.42	12.25	11.99	15.96	10.30	21.67	11.03	3.91	10.72	7.37	12.59	10.33	7.54	6.62	10.87	14.91	10.64	12.61	14.46	14.60	14.42	12.67	17.30	12.21
13.62	10.73	9.58	10.86	11.61	10.83	15.54	9.57	18.77	10.11	3.57	9.58	6.70	11.35	10.05	8.50	5.87	9.90	16.20	9.37	12.04	13.45	14.56	13.78	11.72	16.19	11.14
13.40	10.32	9.11	9.90	11.45	10.48	15.32	9.11	17.90	9.62	3.61	9.38	6.62	11.00	9.92	8.05	5.76	9.44	16.00	8.96	11.79	13.18	14.26	13.54	11.29	16.34	10.79
14.07	9.38	8.74	8.94	10.33	10.17	14.22	8.75	16.83	9.05	3.24	9.11	6.97	11.17	9.15	6.70	5.53	9.14	14.79	8.28	11.59	13.26	13.71	13.87	11.08	14.05	9.99
14.22	10.27	9.74	10.21	10.93	10.90	15.06	9.07	17.20	9.68	3.23	9.43	7.55	11.63	9.53	6.98	5.85	9.95	14.11	9.12	12.29	13.63	13.79	14.24	11.34	13.77	11.03
13.76	11.35	10.49	11.97	10.60	11.83	13.95	9.00	16.63	11.54	4.14	11.49	8.20	11.77	10.30	9.74	6.21	9.05	17.36	8.77	12.21	13.60	12.44	13.27	11.83	17.28	11.17
12.91	10.45	9.52	11.06	9.66	11.09	13.12	8.11	15.97	10.52	3.47	10.63	7.34	10.90	9.51	8.81	5.24	8.04	16.23	8.03	11.34	12.85	11.46	12.37	10.92	16.28	10.33
14.93	11.36	10.24	11.82	12.61	12.58	17.52	10.32	18.38	10.62	4.23	10.77	8.23	13.24	11.12	8.68	6.87	10.99	17.38	10.49	12.94	14.00	15.09	14.22	12.72	18.06	12.59
15.29	12.41	10.97	13.29	13.14	13.20	18.59	11.11	19.01	11.41	4.64	11.30	8.91	14.04	11.80	9.42	7.19	11.74	17.53	11.35	13.47	14.51	15.34	14.83	13.50	18.51	13.35
13.87	9.73	8.45	11.15	10.06	9.85	14.00	8.51	11.66	10.88	3.77	9.80	6.40	10.68	9.16	6.02	5.55	8.77	12.21	9.45	10.94	12.60	11.04	11.11	10.83	16.84	9.63
13.87	9.73	8.45	11.15	10.06	9.85	14.00	8.51	11.66	10.88	3.77	9.80	6.40	10.68	9.16	6.02	5.55	8.77	12.21	9.45	10.94	12.60	11.04	11.11	10.83	16.84	9.63
13.59	10.38	8.60	10.18	10.19	10.40	12.92	8.01	11.36	10.44	3.45	9.70	6.04	10.79	9.17	5.86	5.99	8.90	13.71	8.42	11.29	12.17	10.75	10.60	11.20	15.11	9.76
15.04	11.74	9.59	10.96	11.62	11.71	14.49	9.38	12.90	11.76	4.62	10.93	7.15	11.89	10.56	6.93	7.20	10.12	14.90	9.62	12.70	13.63	12.24	11.79	12.58	16.74	10.92
12.79	11.18	9.17	11.23	11.15	10.73	14.68	8.99	13.46	11.74	3.77	10.55	6.89	11.56	9.71	6.20	6.32	8.79	13.77	9.36	11.60	13.43	11.77	11.98	11.48	14.97	10.29
13.17	11.88	9.22	14.37	11.52	11.91	14.80	9.89	13.66	11.86	3.81	11.49	6.89	12.76	10.67	7.11	6.72	9.73	16.14	10.08	11.93	12.43	11.95	12.42	11.96	15.47	11.32
15.48	10.83	9.61	10.91	11.71	11.33	14.16	9.53	13.24	11.79	4.06	10.81	6.90	11.84	10.23	7.12	6.76	10.00	14.79	10.39	12.37	14.02	14.63	12.30	12.50	17.54	10.98
14.63	10.62	9.11	13.49	11.14	12.04	13.54	9.56	12.26	11.04	3.38	10.80	6.39	12.37	9.92	7.30	5.81	10.18	15.97	10.04	11.50	12.57	13.71	12.06	11.93	17.24	11.34
13.62	10.99	8.96	12.27	10.88	10.49	16.12	9.09	12.89	11.99	4.01	10.27	6.65	11.04	10.03	6.98	6.27	8.28	13.70	9.87	11.85	13.53	11.86	11.00	11.52	16.29	10.45
12.67	10.28	8.28	11.96	9.91	10.20	15.70	8.69	12.38	11.46	3.61	9.84	6.09	10.61	9.78	6.33	5.70	7.74	13.35	9.13	11.27	12.10	11.10	10.82	10.71	15.54	9.83
15.39	11.74	10.13	11.38	11.12	11.49	11.84	8.75	14.34	11.25	4.02	10.79	7.20	11.66	9.94	6.39	6.09	9.82	15.94	9.55	12.68	13.53	12.69	12.59	12.07	16.41	11.15
15.86	12.38	10.36	11.38	12.06	12.97	12.61	10.36	15.26	11.59	4.19	12.52	7.69	13.42	10.91	7.53	6.81	10.83	17.99	10.35	13.17	13.49	13.15	13.94	12.88	17.32	12.56
14.15	10.30	9.24	11.15	10.47	10.14	15.50	8.63	13.01	10.71	3.79	10.07	6.46	10.88	9.68	6.40	5.67	8.55	13.17	9.41	11.20	12.79	11.30	12.20	11.20	15.48	10.33
14.15	10.30	9.24	11.15	10.47	10.14	15.50	8.63	13.01	10.71	3.79	10.07	6.46	10.88	9.68	6.40	5.67	8.55	13.17	9.41	11.20	12.79	11.30	12.20	11.20	15.48	10.33
13.27	10.53	8.44	11.49	10.38	10.09	16.38	9.06	12.23	10.77	3.98	9.25	7.02	10.94	10.04	6.43	5.97	7.69	12.83	8.57	10.94	12.56	11.60	11.10	11.38	16.62	9.64
13.90	11.46	9.31	12.53	11.23	11.41	17.41	9.76	13.14	11.56	4.40	9.89	7.52	11.71	10.71	7.25	6.68	8.57	13.66	9.39	11.88	13.25	12.21	11.88	12.18	17.20	10.56
14.77	11.11	9.97	12.79	11.78	11.47	15.86	9.49	14.98	10.70	4.02	11.34	8.17	12.27	10.36	7.18	6.46	10.47	15.06	10.49	12.24	13.54	13.75	13.29	12.03	18.00	11.58
16.00	12.64	11.65	14.55	13.36	12.96	17.66	11.07	16.62	12.33	5.16	12.73	9.73	13.50	11.82	8.62	8.08	12.11	16.44	12.02	13.93	15.04	15.10	14.77	13.61	19.48	13.01
13.21	10.48	9.45	10.70	10.85	11.30	13.07	8.93	13.65	10.99	3.54	10.30	7.54	11.44	9.50	5.76	6.10	10.11	13.43	9.41	11.85	13.33	13.05	12.37	11.57	12.92	10.88
13.16	10.54	9.58	10.62	11.01	11.50	13.35	9.13	13.91	11.17	3.59	10.42	7.60	11.30	9.53	5.77	6.31	10.16	13.06	9.50	12.00	13.72	13.06	12.53	11.69	12.70	11.03
13.75	10.11	9.13	11.63	10.45	10.83	13.89	8.80	13.31	10.35	3.58	10.43	6.89	11.54	9.70	6.01	5.65	9.00	13.34	9.47	11.04	12.78	10.40	12.28	11.30	16.19	10.42
13.84	10.22	9.38	11.73	10.57	10.88	13.94	8.99	13.54	10.55	3.68	10.55	6.86	11.47	9.83	6.09	5.71	9.06	12.97	9.57	11.31	12.82	10.36	12.61	11.45	16.32	10.50
11.35	10.22	8.98	10.86	9.87	10.59	14.06	8.20	13.09	10.45	3.28	10.07	7.49	11.29	8.95	7.19	5.15	8.77	14.23	8.64	11.09	13.13	12.69	11.65	11.32	13.65	10.35
12.21	11.02	9.90	11.68	10.63	11.15	14.60	8.95	13.89	11.19	3.82	10.97	8.01	11.94	9.69	7.85	5.81	9.42	14.53	9.46	12.01	13.98	13.07	12.48	12.13	14.27	11.14
15.24	10.42	8.39	11.37	10.65	11.27	16.10	8.75	13.71	9.76	3.47	8.92	7.08	11.35	9.77	6.54	6.05	9.32	14.06	9.30	11.66	12.73	13.93	11.95	11.48	14.68	10.55
15.05	10.28	8.43	11.02	10.62	10.96	15.93	8.62	13.64	9.64	3.45	8.81	7.06	11.09	9.61	6.38	5.88	8.98	13.36	9.24	11.68	12.50	13.63	11.87	11.39	14.17	10.40
15.14	12.00	10.39	13.39	12.55	12.22	17.34	10.31	13.64	11.52	4.91	11.49	8.36	12.91	11.26	7.33	7.44	9.97	14.83	11.38	12.78	14.25	14.52	12.86	13.10	19.11	11.82
15.69	12.93	11.22	14.69	13.22	12.82	17.79	11.09	14.43	12.56	5.26	12.09	9.13	13.91	11.84	7.93	7.88	10.62	15.13	12.00	13.51	14.68	14.84	13.12	13.68	19.42	12.76
13.19	10.38	8.88	11.98	10.45	10.22	15.52	8.75	12.16	11.61	3.87	10.21	6.80	11.05	9.73	5.82	5.54	8.59	13.20	9.76	10.95	12.34	10.29	11.37	11.67	16.44	10.24
13.90	10.76	9.15	11.59	11.09	10.17	16.11	8.61	12.19	11.72	4.07	10.56	7.23	10.95	9.92	6.40	6.12	8.54	13.81	10.20	11.37	13.39	11.03	11.46	12.28	16.74	10.24
13.47	10.73	9.32	11.59	11.36	10.74	16.12	8.60	12.48	11.79	4.06	10.79	7.51	11.24	10.18	6.43	6.32	9.30	13.22	10.04	11.62	13.39	10.87	11.91	11.56	16.27	10.49
14.34	11.85	9.78	11.59	12.19	10.74	17.17	10.01	13.12	12.02	4.34	11.99	7.45	11.24	10.92	7.68	6.70	10.55	13.22	11.09	11.90	13.34	11.11	12.94	12.58	17.57	12.08
17.54	12.91	11.50	14.50	13.02	12.59	19.60	10.73	14.05	13.73	5.26	12.96	8.49	12.91	12.16	8.39	8.58	10.72	16.61	11.56	13.83	15.53	15.32	12.54	13.89	19.18	12.60
16.52	11.68	10.27	13.00	11.76	11.73	19.18	9.65	13.15	12.41	4.52	11.66	7.23	11.98	11.25	6.98	7.05	9.52	15.58	10.23	12.42	13.63	14.37	11.74	12.81	18.18	11.35
11.94	10.52	8.87	11.99	11.10	11.08	15.60	8.70	12.06	11.83	3.87	10.31	6.33	11.13	9.58	6.22	6.55	8.47	13.27	9.65	11.20	12.95	11.91	10.80	11.80	16.13	10.34
11.94	10.52	8.87	11.99	11.10	11.08	15.60	8.70	12.06	11.83	3.87	10.31	6.33	11.13	9.58	6.22	6.55	8.47	13.27	9.65	11.20	12.95	11.91	10.80	11.80	16.13	10.34
14.34	11.63	9.75	11.69	11.39	11.37	18.92	9.19	12.99	10.63	4.24	11.42	7.69	12.16	10.55	6.80	6.82	10.69	14.06	10.16	12.08	13.82	13.75	12.14	12.75	18.29	11.30
14.03	11.42	9.57	11.46	11.09	11.22	17.48	8.75	12.68	10.37	3.93	11.13	7.32	11.63	10.23	6.54	6.72	10.42	14.21	9.81	11.93	13.80	13.44	11.74	12.25	17.49	10.94
14.66	10.79	9.39	10.88	11.06	11.08	16.05	8.48	13.07	10.53	4.00	10.82	8.00	11.53	10.30	6.67	6.49	10.25	13.70	9.41	11.85	13.34	13.81	12.02	12.27	18.57	10.94
14.66	10.79	9.39	10.88	11.06	11.08	16.05	8.48	13.07	10.53	4.00	10.82	8.00	11.53	10.30	6.67	6.49	10.25	13.70	9.41	11.85	13.34	13.81	12.02	12.27	18.57	10.94
12.77	10.10	8.72	12.16	10.43	10.52	17.94	8.54	12.18	9.85	3.60	9.73	7.00	11.03	10.04	6.88	5.93	9.09	13.28	8.48	10.81	12.62	11.58	11.14	11.39	16.28	9.79
13.19	10.59	9.05	12.66	10.68	10.90	18.33	8.77	12.47	10.21	3.89	10.21	7.32	11.42	10.52	7.33	6.32	9.44	13.45	8.84	11.21	13.11	11.98	11.54	11.80	16.90	10.23
15.24	12.34	10.70	13.54	12.46	12.42	17.96	9.79	13.10	12.74	5.43	12.36	9.19	13.33	11.60	7.76	7.62	11.03	14.65	11.56	12.93	14.55	14.26	13.01	12.16	18.50	12.38
16.76	13.56	11.90	14.61	14.12	13.33	19.12	11.04	14.64	14.21	6.73	13.69	10.42	14.27	12.76	8.82	9.21	12.41	16.00	12.82	14.32	16.11	15.69	13.41	15.32	19.48	13.46

raw qPCR data efficiency corrected
