# Supplementary material for: The expression signatures in liver and adipose tissue from obese Göttingen Minipigs reveal a predisposition for healthy fat accumulation
Source: Nutr Diabetes. 2020 Mar 23;10:9. doi: 10.1038/s41387-020-0112-y (PMC7090036; doi:10.1038/s41387-020-0112-y)
Supplement: Supplementary file 4 — S3 Table [file 41387_2020_112_MOESM4_ESM.pdf]

S3. Raw qPCR data from SAT

Samples	ABCA1	ABCG1	ACACA	ADCY5	ADIPOQ	ADM	ADRBK2	AKT2	ANGPTL4	AQP7	CD40	CLU	COL6A1	CXCL14	CXCR4	DGAT2	DICER1	DUSP10	EBF2	ELOVL4	ELOVL6	FABP4	FADS1	FAS	FGF21
221516.1	12.93	17.80	10.69	13.84	5.81	14.15	14.39	10.41	11.17	13.66	18.12	13.69	9.45	15.22	15.51	8.21	11.74	13.11	17.41	14.90	7.77	4.29	17.24	14.57	18.82
221516.2	12.93	17.80	10.69	13.84	5.81	14.15	14.39	10.41	11.17	13.66	18.12	13.69	9.45	15.22	15.51	8.21	11.74	13.11	17.41	14.90	7.77	4.29	17.24	14.57	18.82
319021.1	12.66	17.27	11.22	14.51	6.66	13.22	14.26	10.76	12.03	13.22	16.98	12.54	9.25	14.49	15.66	8.31	12.14	12.19	16.26	14.36	7.55	4.82	15.52	14.51	17.99
319021.2	12.66	17.27	11.22	14.51	6.66	13.22	14.26	10.76	12.03	13.22	16.98	12.54	9.25	14.49	15.66	8.31	12.14	12.19	16.26	14.36	7.55	4.82	15.52	14.51	17.99
319357.1	12.49	18.96	10.42	14.22	6.31	13.67	14.81	11.16	12.02	13.77	17.33	11.52	10.48	15.97	15.47	9.17	12.26	13.08	15.89	13.28	8.56	5.00	16.48	15.51	20.05
319357.2	12.33	18.65	10.15	14.11	6.06	13.71	14.51	11.02	11.75	13.73	17.14	11.32	10.22	15.57	15.32	9.06	11.94	13.05	15.91	12.98	8.18	4.69	16.68	15.15	18.94
319677.1	12.00	16.82	9.42	14.18	5.89	13.51	13.36	10.73	12.39	12.22	16.53	14.57	8.66	14.54	15.73	7.56	11.73	12.90	15.71	14.42	6.61	4.50	16.45	14.70	20.63
319677.2	11.71	16.68	9.04	13.91	5.62	13.35	12.82	10.40	12.19	12.36	16.94	14.47	8.38	14.37	15.73	7.35	11.40	12.81	15.75	14.09	6.32	4.48	16.69	14.09	18.72
319703.1	10.92	16.15	9.10	14.00	5.56	11.44	12.48	10.38	10.82	11.88	16.28	14.53	8.40	14.58	15.52	7.20	11.41	10.98	14.77	13.12	5.27	4.20	17.13	15.40	18.68
319703.2	11.39	16.77	9.51	14.25	5.80	11.82	12.99	10.69	11.17	12.21	16.55	14.93	8.71	14.76	15.84	7.24	11.85	11.23	15.05	13.33	5.60	4.31	17.33	15.64	19.95
319876.1	13.04	17.55	10.76	14.13	5.95	12.23	13.91	11.06	10.67	12.03	16.59	14.09	9.67	15.62	16.51	8.76	12.25	13.08	15.76	14.08	6.71	4.31	17.69	15.06	18.83
319876.2	12.59	16.77	10.42	13.74	5.55	11.73	13.39	10.73	10.22	11.41	15.95	13.19	9.37	15.15	16.14	8.37	11.61	12.47	15.05	13.75	6.34	4.02	16.80	14.53	18.52
320462.1	11.58	16.06	9.78	14.00	5.27	11.70	13.19	10.49	10.19	10.93	15.41	13.61	8.46	13.63	16.02	7.54	11.16	11.95	13.73	13.59	5.88	3.98	15.94	13.66	18.41
320462.2	11.73	16.43	10.07	14.31	5.49	11.92	13.46	10.77	10.44	11.03	15.83	13.92	8.72	13.91	16.41	7.78	11.42	12.18	14.04	13.41	6.11	4.15	16.31	14.21	18.75
221497.1	13.01	16.57	10.01	14.02	5.82	11.86	13.25	10.55	11.58	12.04	16.51	14.69	9.37	14.90	14.72	7.62	12.06	11.31	15.13	14.02	5.64	4.33	17.33	14.62	18.79
221497.2	13.00	16.41	9.88	13.93	5.72	11.74	12.94	10.38	11.44	11.72	16.34	14.46	9.24	14.89	14.52	7.45	12.12	11.36	15.01	13.48	5.56	4.33	16.85	14.46	18.21
221510.1	12.72	17.02	9.75	14.14	5.51	11.55	13.09	10.58	10.44	11.71	16.05	13.45	9.07	13.81	16.54	7.66	11.85	11.86	14.57	14.21	5.85	4.36	16.88	15.49	19.88
221510.2	12.21	16.23	9.20	13.44	4.91	10.97	12.59	9.97	9.67	11.20	15.21	12.86	8.62	12.95	15.48	7.20	11.16	11.25	14.24	13.55	5.24	3.96	16.09	14.83	19.01
221538.1	12.06	16.42	9.82	13.68	5.42	10.94	12.19	9.79	9.16	11.68	15.25	12.51	8.17	13.03	13.09	7.88	11.11	11.57	14.41	12.96	4.81	3.76	15.55	14.49	18.86
221538.2	11.94	16.86	9.14	13.63	5.30	11.47	12.34	9.84	8.92	11.80	15.17	12.86	8.19	13.23	13.25	7.94	11.36	11.71	15.02	13.19	5.20	4.11	15.95	14.96	19.43
319035.1	12.43	17.93	10.91	14.27	6.52	12.33	13.56	11.26	11.29	12.09	16.10	11.98	8.91	13.15	15.39	8.90	12.18	12.60	15.54	14.15	7.18	4.71	15.93	14.27	20.46
319035.2	12.44	17.96	10.81	14.45	6.43	12.62	13.65	11.17	11.11	12.36	16.12	12.29	8.90	13.27	15.37	8.84	12.20	12.68	15.87	13.94	7.17	4.77	16.30	14.41	18.95
319239.1	10.84	15.82	10.14	12.81	5.77	11.07	12.41	9.62	9.93	12.08	15.26	12.89	7.66	13.02	13.33	7.43	11.07	11.38	14.83	11.22	5.70	4.00	15.25	13.28	18.03
319239.2	11.61	16.31	10.72	13.24	6.28	11.47	12.82	10.37	10.62	12.27	15.65	13.32	8.35	13.17	13.91	7.87	11.78	12.08	15.01	11.63	6.14	4.36	15.83	14.23	18.97
319389.1	13.03	18.53	9.81	14.25	6.54	12.76	13.82	10.57	10.14	13.66	17.78	14.31	9.08	15.94	14.19	8.26	12.20	12.99	16.45	14.55	6.48	4.66	17.38	15.79	19.80
319389.2	13.36	19.69	9.73	13.84	6.62	13.73	14.72	9.99	9.53	15.36	18.63	14.87	9.10	15.73	13.47	8.10	11.61	12.94	17.85	14.38	7.15	4.30	18.58	16.37	20.77
319798.1	12.03	18.00	9.88	13.69	5.42	13.85	13.39	9.95	10.10	13.13	17.11	11.89	8.31	13.33	14.92	7.79	11.04	11.67	15.73	13.73	7.65	4.10	16.41	14.12	18.27
319798.2	11.64	17.45	9.48	13.44	5.25	13.33	12.77	9.67	9.74	12.38	16.29	11.29	7.66	13.03	14.30	7.32	10.90	11.54	15.37	13.36	7.20	4.00	15.94	13.84	18.23
319799.1	12.13	17.46	10.70	14.40	6.10	12.89	14.02	10.29	10.68	14.59	18.57	14.99	9.26	14.62	14.93	7.88	12.17	12.48	16.80	14.55	7.68	4.75	17.76	15.86	18.98
319799.2	11.22	16.02	9.95	13.68	5.32	11.15	12.89	10.07	9.99	12.88	17.01	13.61	8.60	13.69	14.29	7.44	11.51	11.45	15.51	13.84	6.20	4.25	16.38	14.73	18.98
319875.1	12.08	17.71	9.72	13.14	5.92	12.29	12.66	10.15	9.06	12.39	16.01	11.91	7.94	14.05	13.63	7.25	11.25	11.83	15.57	13.76	5.76	3.98	16.67	13.53	19.52
319875.2	12.08	17.71	9.72	13.14	5.92	12.29	12.66	10.15	9.06	12.39	16.01	11.91	7.94	14.05	13.63	7.25	11.25	11.83	15.57	13.76	5.76	3.98	16.67	13.53	19.52
320241.1	12.13	18.27	10.11	13.41	6.71	14.04	13.99	10.04	10.50	15.05	17.57	13.46	8.82	19.73	14.66	9.03	11.13	11.62	17.55	13.81	7.32	4.69	16.87	21.71	19.59
320241.2	12.12	17.65	10.27	13.46	6.56	13.54	13.76	10.09	10.51	14.49	17.40	13.17	8.45	18.95	14.72	8.93	11.10	11.68	16.90	13.76	7.24	4.70	16.41	19.81	18.52
320316.1	11.90	17.03	11.04	14.30	6.84	13.81	13.01	10.93	10.38	13.96	16.64	10.66	8.48	12.36	13.99	8.39	11.78	13.09	15.82	13.80	7.22	4.16	16.20	14.15	18.95
320316.2	11.48	16.81	10.65	13.84	6.44	13.33	12.54	10.28	9.80	13.37	16.02	10.12	8.10	11.91	13.56	7.96	11.48	12.53	15.46	13.47	6.90	3.94	15.70	13.52	18.19
320317.1	13.31	17.71	10.85	14.72	6.44	11.50	12.99	11.19	11.05	11.53	15.34	12.18	9.24	15.47	15.97	8.74	11.95	11.94	14.85	13.96	7.52	4.51	16.85	14.12	19.40
320317.2	12.42	16.65	10.15	13.61	5.64	10.47	12.06	10.29	10.13	10.40	13.97	11.25	8.41	14.48	15.02	7.82	11.14	11.04	13.81	13.25	6.72	3.89	15.93	13.02	18.46
320351.1	12.10	15.70	9.73	13.39	5.91	10.03	12.45	10.07	9.88	11.16	15.26	12.28	7.35	12.06	13.80	7.91	11.15	12.16	14.22	13.37	5.52	3.90	14.70	13.58	18.30
320351.2	12.06	15.70	9.52	13.19	5.75	10.00	12.03	9.80	9.75	11.06	15.22	12.14	7.47	11.95	13.47	7.72	11.01	11.95	14.08	13.10	5.38	3.74	14.83	13.18	18.23
320440.1	11.87	16.10	10.08	13.97	5.45	11.19	12.74	10.42	11.08	11.79	16.33	14.17	8.59	13.41	14.19	7.23	11.56	11.80	14.25	13.58	5.48	4.21	16.21	13.85	19.22
320440.2	12.12	16.58	10.32	14.21	5.66	11.57	12.92	10.48	11.27	12.16	16.55	14.47	8.85	13.47	14.23	7.63	11.78	11.75	14.70	13.86	5.95	4.30	16.27	13.80	19.29
319427.1	14.14	18.41	10.52	14.47	8.26	13.49	15.02	11.30	11.90	14.42	15.33	13.18	8.44	12.75	15.63	8.78	11.99	13.95	14.45	11.42	7.83	5.78	13.44	14.30	18.84
319427.2	14.14	18.41	10.52	14.47	8.26	13.49	15.02	11.30	11.90	14.42	15.33	13.18	8.44	12.75	15.63	8.78	11.99	13.95	14.45	11.42	7.83	5.78	13.44	14.30	18.84
319778.1	13.97	19.26	10.59	14.93	6.09	14.06	13.36	10.90	11.97	12.93	18.10	14.29	8.96	13.07	16.46	8.24	11.53	11.27	16.03	12.70	6.84	5.15	16.17	15.28	19.37
319778.2	14.02	19.61	10.57	14.86	6.00	13.75	13.05	10.78	11.95	12.79	17.33	14.48	9.04	12.99	16.67	8.17	11.50	11.34	15.61	12.39	6.72	5.00	16.08	15.51	20.30
319860.1	13.17	17.50	9.55	14.77	5.33	12.22	12.75	9.96	13.65	12.36	17.22	14.04	8.17	12.33	16.25	6.54	11.59	11.07	14.83	12.70	5.70	4.57	15.69	14.88	19.91
319860.2	13.46	18.22	9.94	15.07	5.73	12.79	13.19	10.44	14.01	13.12	17.71	14.56	8.44	12.66	16.86	6.88	12.04	11.55	15.26	12.95	6.01	4.69	16.26	15.64	20.47
319922.1	13.40	20.28	10.28	15.09	4.87	12.15	12.88	9.91	11.50	12.09	17.61	14.92	8.54	14.03	15.29	6.90	11.21	11.14	14.79	13.77	6.51	4.23	15.19	13.97	19.03
319922.2	13.72	20.54	10.43	15.42	4.83	12.67	13.44	10.19	11.58	12.89	18.12	15.18	8.69	14.17	15.41	7.03	11.33	11.37	15.29	13.59	6.93	4.34	15.67	14.53	19.16
320207.1	14.00	18.98	9.40	15.53	5.64	13.62	13.49	10.23	12.28	13.33	18.44	14.18	9.65	14.54	16.78	6.93	11.81	11.86	15.94	14.81	6.99	4.61	16.96	15.18	19.63
320207.2	14.03	18.91	9.28	15.55	5.60	13.61	13.73	9.95	12.14	13.25	18.51	14.01	9.37	14.35	16.84	6.79	11.73	11.66	16.00	14.84	6.76	4.56	17.00	14.68	20.49
320340.1	13.33	19.63	10.37	14.25	5.64	13.17	13.96	9.65	9.65	13.55	17.35	13.95	9.78	15.66	16.66	8.49	11.55	12.77	15.49	14.22	7.81	4.11	17.53	14.42	19.74
320340.2	12.74	19.05	9.89	13.97	5.02	12.52	13.21	9.20	9.05	12.56	17.02	13.32	9.37	15.12	16.14	7.75	11.06	12.30	14.31	13.47	7.30	3.90	17.03	13.93	18.67
320411.1	13.90	20.97	11.08	16.00	6.81	13.81	13.83	10.63	13.37	14.04	18.17	14.00	9.10	18.73	16.28	8.56	10.88	11.15	16.72	14.40	7.19	5.27	17.14	14.88	20.15
320411.2	13.86	20.05	10.82	15.73	6.57	13.59	13.22	10.51	13.16	13.68	18.30	13.77	8.86	18.44	16.02	8.59	10.69	10.99	16.36	14.48	7.06	5.11	16.60	15.32	19.62

raw qPCR data efficiency corrected

ref genes

IL6	GCLM	GLUT4	GNAS	GPC4	GRB10	ICAM-1	IDS	NR3CI	IRX3	ISLR	JAG1	KLB	.CN2 (NGAL	LDLR	LEP	LITAF	LPL	LSS	MGMT	MKL1	MOCOS	MTOR	MYC	NCOR2	NEGR1
15.74	13.06	12.60	9.16	15.04	12.29	16.96	10.20	11.75	14.77	14.11	12.15	10.12	14.23	13.99	9.01	11.68	9.00	15.09	11.26	13.71	15.91	14.54	14.87	11.60	16.83
15.74	13.06	12.60	9.16	15.04	12.29	16.96	10.20	11.75	14.77	14.11	12.15	10.12	14.23	13.99	9.01	11.68	9.00	15.09	11.26	13.71	15.91	14.54	14.87	11.60	16.83
15.42	12.76	13.65	8.91	14.73	12.70	15.46	10.14	11.97	15.82	14.63	12.26	10.66	14.26	13.50	9.18	11.42	8.93	15.21	11.80	13.53	17.03	14.70	14.53	11.24	16.16
15.42	12.76	13.65	8.91	14.73	12.70	15.46	10.14	11.97	15.82	14.63	12.26	10.66	14.26	13.50	9.18	11.42	8.93	15.21	11.80	13.53	17.03	14.70	14.53	11.24	16.16
16.94	12.78	14.33	9.02	15.12	12.17	16.65	10.16	12.19	21.34	14.01	12.36	10.24	11.41	15.91	11.61	11.64	9.09	16.03	12.12	14.28	15.76	14.85	15.05	12.55	17.68
16.30	12.55	14.01	8.83	14.74	11.91	16.59	9.99	11.93	20.87	13.73	12.00	10.02	11.16	15.84	11.39	11.59	8.91	15.90	12.13	14.12	15.47	14.57	14.69	12.40	16.81
14.71	12.25	12.69	8.11	14.06	12.61	15.04	10.13	11.30	15.05	13.76	11.38	10.17	13.32	13.88	8.13	10.89	7.70	14.05	11.30	13.53	16.20	14.28	14.60	11.34	14.60
14.89	12.34	12.41	7.91	13.91	12.36	14.99	9.77	11.29	14.57	13.42	11.02	9.98	12.85	13.41	7.85	10.70	7.80	13.97	10.95	13.26	15.77	14.00	14.03	11.09	14.16
13.74	11.59	11.95	7.57	14.24	12.15	15.06	9.91	10.79	14.70	13.06	11.62	9.41	13.50	14.17	8.90	10.78	7.10	13.21	10.68	13.58	15.53	14.06	12.81	11.04	14.32
14.14	11.86	12.11	7.98	14.23	12.28	15.08	9.95	11.20	14.93	13.07	12.03	9.65	14.04	14.50	9.20	10.92	7.43	13.57	11.01	13.73	15.91	14.23	13.08	11.19	14.41
14.47	11.96	13.76	8.46	14.88	12.72	15.69	9.34	11.71	15.06	14.46	11.84	10.19	13.45	13.95	8.61	11.47	7.61	14.62	11.81	14.04	16.00	14.92	13.95	12.19	15.38
14.16	11.39	13.35	7.90	14.91	12.24	14.79	8.96	11.21	14.67	14.26	11.53	9.67	12.86	13.65	8.15	10.99	6.88	14.04	11.25	13.66	15.94	14.22	13.38	11.78	15.09
13.70	11.40	12.10	7.17	13.59	12.12	15.23	9.97	10.86	14.66	13.10	11.16	8.74	14.21	13.60	8.31	10.81	6.51	12.22	11.00	13.38	15.85	13.78	13.46	10.95	16.90
13.98	11.71	12.28	7.50	13.97	12.32	15.61	10.04	10.91	14.66	13.50	11.45	8.97	14.41	13.88	8.69	11.18	6.74	12.38	11.26	13.63	15.87	14.01	13.66	11.19	16.69
15.07	11.35	12.57	7.94	15.03	12.56	16.08	9.86	11.28	15.34	13.19	11.98	9.55	15.03	13.81	8.29	11.24	6.48	12.12	11.24	13.83	15.91	14.48	13.65	11.09	16.01
14.97	11.31	12.43	7.79	14.86	12.50	15.70	9.47	11.45	15.12	13.01	11.64	9.42	14.92	13.71	8.12	11.16	6.44	11.87	11.19	13.60	15.76	14.22	13.65	11.00	15.74
13.78	11.11	12.12	8.03	15.14	12.66	14.97	10.25	11.06	14.71	13.83	11.34	9.10	14.81	14.51	8.86	11.04	6.78	12.92	11.45	13.69	16.46	14.26	13.04	11.61	15.05
13.34	10.55	11.71	7.42	14.57	11.98	14.52	9.64	10.31	14.13	13.11	10.69	8.53	14.19	13.56	8.30	10.66	6.11	12.38	10.74	13.37	15.19	13.86	12.82	11.06	14.39
12.04	10.19	12.36	7.49	13.60	11.62	14.26	9.97	10.41	14.68	12.73	10.36	9.71	13.71	13.31	7.94	10.61	6.55	11.52	10.43	13.17	14.93	13.48	12.32	10.52	13.68
12.28	10.73	12.30	7.60	13.73	11.86	14.74	9.27	10.52	14.22	12.20	10.58	9.83	13.85	13.00	8.39	10.69	7.10	11.82	10.69	13.14	15.12	13.68	11.66	10.51	13.83
13.92	11.68	13.48	8.49	14.80	12.91	15.59	11.07	11.54	15.74	13.85	11.59	10.99	13.91	14.42	9.27	10.96	7.79	13.65	12.37	14.03	15.91	14.72	13.84	11.50	14.57
14.20	11.90	13.53	8.61	14.78	12.79	16.07	10.92	11.58	15.33	13.89	11.54	11.09	13.76	14.09	9.13	11.01	7.92	13.62	12.18	14.13	15.98	14.85	13.93	11.37	14.54
14.09	10.86	12.38	7.23	13.06	11.73	14.13	9.34	10.31	14.47	13.00	10.23	9.65	13.15	12.91	8.02	9.91	7.19	13.00	10.52	12.66	15.52	13.54	12.64	10.31	14.08
14.26	10.95	13.01	7.76	13.88	12.19	14.56	9.77	10.89	14.97	13.72	11.07	9.81	14.07	13.68	8.51	10.30	7.29	12.75	11.14	13.33	16.28	14.01	13.48	10.68	14.45
14.22	12.57	11.90	8.86	15.16	12.99	15.76	10.21	12.16	16.03	14.24	12.40	10.48	13.80	13.64	9.00	11.22	8.44	13.98	11.41	13.86	17.16	14.45	13.44	11.32	15.23
14.17	13.63	11.42	9.74	14.61	12.81	16.40	9.96	12.31	15.57	14.35	12.46	10.89	13.34	13.15	9.03	12.01	9.39	14.29	11.03	13.69	16.91	14.56	13.82	11.05	14.66
15.00	12.45	12.56	8.15	13.24	11.99	15.01	9.40	11.28	14.23	13.41	11.45	9.77	13.11	13.80	8.89	10.71	8.32	14.67	11.03	12.56	15.46	13.65	13.61	10.90	15.64
14.44	11.75	12.12	7.51	13.01	11.60	14.62	9.11	10.59	14.08	12.80	11.05	9.39	13.19	13.46	8.41	10.23	7.67	14.29	10.71	12.51	15.35	13.44	13.03	10.76	15.09
16.17	14.70	12.71	9.35	14.54	12.52	16.12	10.30	11.99	15.26	13.48	11.92	10.91	14.30	14.14	9.46	11.24	9.90	15.41	11.31	13.93	17.04	14.40	15.05	11.20	17.33
14.89	13.13	12.11	8.08	13.66	11.85	14.98	9.62	10.79	14.55	12.53	11.16	9.66	13.61	13.62	8.43	10.30	8.15	14.44	10.53	13.37	15.92	13.70	14.19	10.71	16.85
14.11	11.46	12.02	7.80	13.75	12.07	14.43	9.23	11.10	14.72	13.58	11.38	9.82	13.53	13.20	8.24	10.01	7.61	13.14	10.68	12.97	16.01	13.67	12.70	10.25	14.07
14.11	11.46	12.02	7.80	13.75	12.07	14.43	9.23	11.10	14.72	13.58	11.38	9.82	13.53	13.20	8.24	10.01	7.61	13.14	10.68	12.97	16.01	13.67	12.70	10.25	14.07
15.16	13.09	11.16	8.76	13.84	10.51	15.25	9.16	11.15	15.09	13.07	11.47	11.13	13.65	13.12	9.71	11.36	11.05	14.74	11.48	12.48	16.27	13.64	13.77	10.73	14.56
15.42	12.39	11.16	8.50	13.65	10.37	15.11	9.01	11.02	14.68	12.97	11.33	10.97	13.68	12.92	9.34	11.01	10.42	14.67	11.55	12.49	16.44	13.42	13.48	10.72	14.69
13.45	13.45	13.63	8.01	13.60	12.47	15.18	11.14	11.51	15.28	14.18	11.71	11.07	13.17	13.62	9.54	10.49	8.55	13.69	11.24	13.04	16.01	14.05	13.24	10.91	15.82
12.99	13.19	13.26	7.70	13.19	12.05	14.64	10.39	10.89	15.07	13.85	11.31	10.73	12.86	13.26	9.46	10.10	8.23	13.19	10.83	12.48	15.43	13.66	12.84	10.54	15.20
13.86	11.56	13.35	8.00	15.03	12.78	12.85	10.58	11.38	15.79	14.69	11.90	9.82	15.24	14.62	8.69	10.80	7.73	13.48	11.74	13.28	15.73	14.62	13.82	11.91	15.45
12.89	10.46	12.31	6.99	14.24	11.81	11.67	9.57	10.31	14.54	13.44	10.68	8.89	14.29	13.74	7.96	9.91	6.79	12.25	10.92	12.71	14.68	13.73	13.01	10.96	14.65
13.81	10.39	12.86	7.19	13.31	11.67	13.90	9.22	10.43	14.67	13.80	10.80	9.70	12.99	13.31	7.42	9.61	6.58	11.78	10.68	12.68	14.89	13.29	12.52	10.51	14.39
13.93	10.26	12.72	7.09	13.34	11.49	13.83	9.07	10.25	14.41	13.50	10.59	9.60	12.85	13.19	7.52	9.61	6.55	11.77	10.46	12.78	14.80	13.34	12.31	10.24	14.36
14.65	11.03	12.68	7.65	14.34	12.22	15.37	9.43	10.78	14.53	13.27	10.89	9.72	13.65	13.90	8.27	11.04	6.67	12.49	11.08	13.77	15.53	14.41	14.20	10.98	16.96
15.18	11.43	12.74	8.02	14.32	12.44	15.53	9.57	10.80	14.84	13.27	11.09	9.95	14.18	14.12	8.55	11.00	7.11	13.07	11.21	13.57	15.74	14.50	14.29	11.21	16.77
14.95	13.32	12.67	8.02	14.50	13.02	15.88	11.21	11.85	18.24	12.92	11.39	10.82	11.91	12.89	12.83	11.30	9.18	14.21	11.96	13.52	17.60	14.01	13.97	11.78	18.48
14.95	13.32	12.67	8.02	14.50	13.02	15.88	11.21	11.85	18.24	12.92	11.39	10.82	11.91	12.89	12.83	11.30	9.18	14.21	11.96	13.52	17.60	14.01	13.97	11.78	18.48
16.95	11.72	12.28	8.11	14.58	11.31	17.39	10.64	11.39	14.67	13.17	11.56	10.69	14.01	13.82	10.42	11.92	8.22	12.93	11.56	14.04	16.37	14.28	14.44	11.39	16.28
16.27	11.69	12.14	8.07	14.40	11.33	16.95	10.28	11.11	14.25	13.03	11.41	10.47	13.81	13.79	10.34	11.96	8.24	12.98	11.39	13.77	16.41	14.43	14.38	11.19	16.13
17.30	12.58	11.38	7.67	14.23	12.42	15.99	8.78	11.00	14.16	12.28	11.29	9.19	13.91	13.65	10.16	11.38	6.47	12.97	10.55	13.33	15.76	14.00	14.87	10.58	13.91
16.80	12.96	11.47	8.04	14.62	12.83	16.39	9.13	11.61	14.65	12.53	11.59	9.61	14.08	14.02	10.49	11.48	7.12	13.63	10.79	13.63	16.24	14.02	15.08	11.02	14.28
17.07	12.42	11.76	7.84	14.80	12.21	16.57	9.10	10.71	14.56	12.79	11.72	9.13	13.53	12.96	9.60	11.32	7.22	13.57	10.88	13.94	15.00	13.79	15.02	10.83	14.12
17.25	13.07	11.81	8.27	14.51	12.28	16.82	9.39	11.13	14.40	12.78	11.82	9.53	13.69	13.14	9.91	11.63	7.88	13.94	11.08	13.95	15.37	14.06	15.51	11.11	14.32
16.54	13.54	11.97	8.57	15.07	12.76	16.64	9.15	11.63	15.89	13.20	11.91	10.33	13.90	13.99	9.48	11.92	7.78	12.76	11.35	14.23	15.70	14.70	15.25	11.54	15.26
16.94	13.55	11.84	8.51	15.11	12.72	16.25	8.85	11.40	15.73	13.31	11.68	10.03	13.84	13.91	9.53	11.62	7.98	12.74	11.25	14.03	15.84	14.55	15.27	11.24	15.02
16.05	13.38	12.52	8.34	14.64	12.13	15.29	9.65	11.51	15.47	14.17	11.44	9.75	13.79	13.23	10.93	11.75	8.98	15.66	11.17	13.36	15.85	14.37	14.26	11.57	17.79
15.67	12.68	12.25	7.97	14.46	11.87	14.96	9.29	11.08	15.21	13.46	11.09	9.21	13.39	12.80	10.33	10.95	8.15	14.94	10.82	13.30	15.43	13.88	13.88	11.31	16.97
17.16	12.04	10.93	8.25	14.06	9.85	16.46	10.80	11.24	15.13	13.51	12.65	11.65	15.00	14.54	10.69	12.54	9.40	15.11	12.14	13.63	16.86	14.13	15.73	11.08	17.85
16.63	11.72	10.62	8.05	14.15	9.59	16.24	10.55	11.00	14.73	13.08	12.45	11.37	14.61	14.50	10.51	12.29	9.10	15.01	11.95	13.41	16.38	13.77	15.71	10.89	17.84

NFKBIA	NPEPL1	OSBPL10	TLR4	PELI2	PN-1	PNPLA2	PON1	PPARD	PRKG1	RDH5	RHOQ	RNF10	RORA	RPS29	SAA	SMAD6	SMPDL3A	SP1	SREBP1	STIM1	SYNE1	TBC1D16	TECPR2	TGFB1	TGFB3
10.74	12.47	14.83	15.25	14.72	12.69	9.38	11.12	13.42	16.53	11.99	10.92	10.60	16.48	12.22	16.64	14.43	9.48	12.62	15.05	11.98	13.86	13.86	12.65	13.73	13.75
10.74	12.47	14.83	15.25	14.72	12.69	9.38	11.12	13.42	16.53	11.99	10.92	10.60	16.48	12.22	16.64	14.43	9.48	12.62	15.05	11.98	13.86	13.86	12.65	13.73	13.75
10.63	12.34	14.92	15.28	13.76	12.17	9.88	12.58	12.78	15.58	12.56	11.10	10.83	15.99	12.12	15.96	14.12	8.75	12.60	15.06	12.54	13.63	14.02	12.92	13.35	13.31
10.63	12.34	14.92	15.28	13.76	12.17	9.88	12.58	12.78	15.58	12.56	11.10	10.83	15.99	12.12	15.96	14.12	8.75	12.60	15.06	12.54	13.63	14.02	12.92	13.35	13.31
10.50	13.32	15.77	14.01	13.67	14.32	9.82	14.13	14.63	15.01	14.14	11.64	11.02	16.24	12.47	14.91	14.20	10.83	12.46	16.69	12.42	13.15	15.13	12.93	13.02	14.68
10.27	13.21	15.84	13.73	13.66	14.38	9.90	13.75	14.45	14.91	13.94	11.27	10.68	16.23	12.02	14.66	14.13	10.50	12.25	16.53	12.15	12.97	15.05	13.00	13.12	14.56
10.55	12.26	14.78	14.66	14.47	11.39	9.30	12.05	12.50	15.21	11.29	10.01	10.70	15.28	11.68	15.83	14.07	8.08	12.09	13.59	11.51	13.16	13.71	12.23	13.04	12.39
10.22	12.04	14.35	14.37	14.25	11.18	9.11	11.89	11.98	14.78	11.09	9.90	10.34	15.46	11.37	16.59	13.78	7.90	11.91	12.85	11.13	12.78	13.27	11.94	12.71	12.22
9.50	11.75	14.45	13.65	13.93	11.15	8.75	12.09	11.89	15.38	11.54	9.09	9.91	14.35	10.65	15.31	12.97	7.36	11.85	13.92	10.84	13.11	13.50	11.87	13.00	13.82
9.78	12.14	14.80	13.90	14.47	11.52	8.97	12.21	12.23	15.96	11.68	9.47	10.20	14.52	11.02	15.67	13.24	7.57	12.21	14.36	11.22	13.39	13.73	12.09	13.22	13.85
10.53	12.72	15.32	15.33	13.29	12.08	9.09	12.08	13.17	15.29	12.16	10.49	10.73	14.74	12.04	15.80	13.52	8.18	12.54	14.64	11.76	13.79	14.48	12.59	14.04	14.38
10.10	12.27	14.60	14.71	12.80	11.76	8.60	11.51	12.69	14.93	11.46	9.86	10.30	14.22	11.27	15.77	13.02	7.56	12.14	14.58	11.51	13.22	13.86	12.34	13.73	14.12
9.76	11.89	14.55	13.47	12.75	10.36	8.37	11.07	12.79	14.66	10.95	8.98	9.93	12.89	8.24	14.80	12.84	7.27	11.60	14.00	11.46	12.80	13.60	11.65	12.77	12.54
10.00	12.18	14.79	14.16	12.90	10.69	8.66	11.29	13.20	15.21	11.28	9.27	10.15	13.12	8.52	15.27	13.05	7.69	11.79	14.25	11.76	13.16	13.82	12.01	13.05	12.78
10.77	12.39	14.90	14.18	13.43	11.57	9.25	11.18	12.55	14.62	11.45	9.15	10.43	13.14	9.29	14.72	13.65	7.67	12.45	14.26	11.79	13.59	14.05	12.24	14.08	14.34
10.57	12.35	14.69	13.90	13.37	11.50	9.00	11.13	12.75	14.98	11.19	9.12	10.46	13.21	9.39	15.07	13.70	7.51	12.18	14.20	11.69	13.67	13.93	12.23	13.51	13.96
10.53	12.27	14.79	14.03	12.63	11.99	9.01	12.08	12.30	16.37	11.63	9.63	10.33	13.73	10.21	14.95	13.53	8.25	12.28	15.23	11.44	13.17	13.91	12.39	13.32	14.04
9.67	11.73	14.08	13.58	12.11	11.63	8.28	11.47	11.42	15.28	11.24	8.89	9.72	13.22	9.78	14.27	13.16	7.46	11.62	14.33	10.68	12.74	13.08	12.01	12.46	13.48
9.68	11.54	13.82	12.60	12.82	11.34	8.16	11.11	11.86	15.22	11.63	8.88	9.69	12.89	9.68	13.79	12.86	6.04	11.88	13.36	10.65	12.45	13.02	11.28	12.77	13.08
9.68	11.51	14.23	12.99	13.22	11.62	8.27	11.34	11.63	15.02	11.83	9.32	9.95	13.13	10.30	14.09	13.13	6.31	11.82	12.56	10.74	12.58	12.99	11.47	12.21	13.10
10.85	12.70	15.65	13.77	13.28	12.01	9.45	14.00	13.60	14.97	12.72	9.79	11.00	14.05	11.25	15.04	14.21	8.23	12.46	14.30	11.69	13.63	14.03	12.61	12.96	12.67
10.92	12.57	15.22	13.91	13.55	12.19	9.45	14.04	13.75	15.27	12.62	10.07	10.95	14.04	11.51	15.13	14.12	8.34	12.52	14.17	11.75	13.37	13.91	12.64	12.77	12.81
10.06	11.39	14.15	12.83	12.72	10.66	8.47	11.48	11.16	14.33	10.95	8.97	9.61	13.56	10.26	14.23	13.14	6.39	11.51	12.98	10.68	11.95	12.71	11.16	12.59	12.90
10.51	12.02	14.70	13.45	13.01	11.16	8.90	11.91	11.89	14.54	11.39	9.17	10.19	13.70	10.15	14.31	13.91	6.71	12.06	13.72	11.44	12.46	13.27	11.59	13.04	13.43
10.92	12.55	15.29	14.66	14.57	12.92	8.70	13.10	12.19	16.21	12.27	10.83	11.08	16.51	12.21	16.48	14.12	8.47	12.67	15.53	11.81	13.59	14.60	12.92	13.66	14.75
10.29	12.37	14.58	15.12	15.39	13.47	9.01	12.83	11.76	17.64	13.15	11.65	10.79	18.20	12.51	16.84	14.47	9.33	13.08	15.61	11.56	13.70	14.71	12.90	13.99	15.10
9.68	11.91	14.10	13.82	13.96	12.64	8.65	12.32	11.96	14.87	13.01	10.75	10.32	16.11	11.77	16.21	13.94	9.16	11.88	14.73	11.07	12.53	13.46	11.98	12.68	12.83
9.62	11.59	14.19	13.53	13.53	11.90	8.24	12.07	11.70	15.02	12.57	10.15	9.93	15.52	11.52	15.73	13.69	8.46	11.34	14.16	10.64	12.31	12.96	11.55	12.26	12.79
10.68	12.59	14.57	15.35	14.71	12.86	9.17	12.89	12.90	15.82	13.27	12.03	11.55	16.47	12.87	16.50	14.09	9.64	12.78	14.29	11.51	13.61	14.21	12.55	13.15	13.40
10.01	11.97	14.05	14.32	13.35	11.56	8.37	11.95	11.95	14.30	12.19	10.31	10.43	15.06	11.40	15.57	13.23	8.05	11.75	13.74	10.90	13.09	13.38	11.79	12.47	12.78
9.49	11.50	13.86	13.10	13.62	11.19	8.45	12.38	11.43	15.15	10.91	9.73	9.85	14.57	10.88	15.66	13.63	7.08	11.76	15.13	10.87	12.39	13.10	12.04	12.40	12.65
9.49	11.50	13.86	13.10	13.62	11.19	8.45	12.38	11.43	15.15	10.91	9.73	9.85	14.57	10.88	15.66	13.63	7.08	11.76	15.13	10.87	12.39	13.10	12.04	12.40	12.65
9.44	11.47	14.15	15.53	14.30	12.96	8.97	14.02	12.71	15.08	13.03	10.59	10.04	14.63	11.60	15.82	13.95	9.04	11.86	12.23	10.90	11.69	13.03	12.06	13.26	12.71
9.40	11.39	14.39	15.09	13.98	12.78	8.84	13.62	12.81	14.81	12.83	10.24	9.85	13.88	11.47	15.89	13.68	8.85	11.73	12.14	10.62	11.44	13.08	12.15	12.94	12.42
10.61	12.05	14.88	12.82	14.43	11.88	9.32	13.10	11.75	15.24	11.92	10.68	10.81	16.09	11.38	15.61	14.06	8.77	12.09	14.36	11.40	12.58	13.75	12.47	12.01	12.88
10.08	11.74	14.55	12.50	13.86	11.51	8.91	12.44	11.41	14.41	11.57	10.24	10.39	15.44	10.97	14.86	13.66	8.29	11.60	13.64	11.14	12.29	13.37	12.07	11.36	12.53
8.67	12.86	15.15	13.87	12.51	11.81	8.72	13.73	13.52	14.33	11.91	9.47	10.71	13.57	10.28	15.27	14.04	8.69	12.20	15.23	11.90	12.51	14.45	12.46	12.69	13.86
7.72	12.03	13.57	12.73	11.53	10.75	7.79	12.86	12.34	13.85	10.97	8.46	9.81	12.87	9.30	14.10	13.03	7.72	11.31	14.56	10.88	11.70	13.47	11.46	11.69	12.97
9.82	11.53	14.43	12.31	12.37	11.26	7.94	12.29	11.00	14.24	11.00	8.70	9.82	12.67	9.65	14.24	12.47	6.70	11.57	13.94	10.99	12.08	12.88	11.50	12.39	12.93
9.59	11.37	14.25	12.22	12.27	10.85	7.89	12.11	10.97	13.94	10.72	8.57	9.56	12.79	9.84	14.36	12.60	6.63	11.29	13.60	10.78	12.07	12.72	11.26	12.34	12.82
10.38	11.94	13.92	13.90	12.83	11.22	8.59	12.24	12.08	14.57	11.57	9.53	10.34	13.58	10.99	15.41	13.51	6.82	11.92	14.03	11.06	13.41	13.21	11.83	13.21	13.52
10.67	12.04	14.25	14.13	13.16	11.53	8.73	12.31	12.22	14.85	11.80	10.04	10.51	14.05	11.36	16.08	13.83	7.06	12.12	14.12	11.19	13.25	13.60	12.12	13.32	13.69
10.14	13.05	14.22	14.39	12.44	12.82	10.49	16.30	12.73	13.19	13.77	11.74	10.47	15.01	10.95	15.88	14.45	12.64	11.86	14.79	11.39	12.72	15.01	12.57	12.69	13.39
10.14	13.05	14.22	14.39	12.44	12.82	10.49	16.30	12.73	13.19	13.77	11.74	10.47	15.01	10.95	15.88	14.45	12.64	11.86	14.79	11.39	12.72	15.01	12.57	12.69	13.39
11.18	12.19	15.40	15.32	14.02	14.14	9.23	14.12	14.51	15.55	11.90	10.46	10.54	13.71	11.25	16.72	14.81	10.20	12.19	15.83	11.54	12.58	13.98	12.13	14.63	14.21
11.03	12.07	15.95	15.03	13.51	14.57	9.12	13.89	14.21	15.58	11.84	10.42	10.48	13.53	11.06	15.51	14.55	9.99	12.17	15.44	11.29	12.73	13.69	11.88	14.22	14.27
10.56	12.04	14.34	13.97	12.95	13.22	8.47	12.10	11.75	15.35	11.19	10.33	10.29	15.10	11.00	15.15	13.71	8.56	11.73	14.21	11.18	13.28	13.70	11.71	14.06	13.41
10.88	12.29	14.77	14.61	13.51	13.35	8.72	12.50	12.12	15.43	11.47	10.68	10.58	15.83	11.44	15.65	14.43	9.17	12.17	14.19	11.41	13.30	13.91	12.13	14.06	13.53
10.81	12.01	14.27	13.69	13.33	13.38	8.61	12.48	13.59	15.57	10.74	10.33	10.51	14.94	11.18	16.00	14.21	9.06	11.77	15.22	11.43	13.17	13.74	11.93	14.22	13.47
10.77	12.17	14.40	14.44	13.81	14.04	8.82	12.70	13.61	16.22	11.12	10.96	10.57	16.07	11.59	17.00	14.72	9.46	12.09	15.47	11.82	13.24	13.83	12.28	14.73	13.77
10.66	12.69	14.84	15.28	14.51	13.92	8.98	13.44	12.78	16.09	11.81	11.21	10.88	17.00	12.36	16.93	14.79	10.34	12.41	15.14	12.18	13.76	14.49	12.62	14.18	14.40
10.68	12.71	14.93	15.49	14.40	14.05	8.75	13.33	12.58	16.67	11.56	11.10	10.87	16.77	12.39	17.36	14.26	10.25	12.48	14.81	12.01	13.83	14.58	12.47	13.84	14.42
9.99	12.44	15.44	15.12	14.18	12.62	8.31	15.80	13.39	16.39	12.87	10.87	10.59	16.74	11.80	14.84	13.22	10.34	12.24	16.05	11.43	13.23	14.59	12.69	13.60	14.52
9.44	12.08	15.43	14.60	13.82	11.99	7.75	15.16	13.05	15.81	12.28	10.26	10.13	15.82	11.24	14.31	12.78	9.56	11.91	16.05	11.19	12.88	14.13	12.23	13.24	14.10
10.93	11.49	15.44	15.46	14.30	14.92	9.42	13.96	13.65	15.47	13.23	10.23	10.54	13.65	11.68	16.86	15.43	10.47	12.39	14.93	10.91	11.83	14.04	12.27	14.28	14.93
10.85	11.40	15.25	15.10	13.76	14.64	9.20	13.60	13.40	15.03	12.83	10.00	10.43	13.57	11.49	16.61	15.38	10.15	12.14	14.55	10.68	11.67	13.71	11.94	14.10	14.35

TNF	TNS3	USF1	ACTB	HPRT1	TBP	YWHAZ
17.36	10.29	17.61	8.42	21.97	15.08	13.44
17.36	10.29	17.61	8.42	21.97	15.08	13.44
16.98	10.73	16.97	7.74	20.23	14.67	11.17
16.98	10.73	16.97	7.74	20.23	14.67	11.17
17.98	11.35	18.16	8.66	20.92	14.76	12.71
17.78	11.11	17.77	8.79	20.33	14.85	12.51
17.96	10.69	16.50	7.74	20.61	13.90	10.80
17.90	10.30	16.47	7.81	21.37	13.88	11.17
17.73	10.54	16.37	6.97	18.98	13.18	8.93
17.38	10.92	16.46	7.12	18.94	13.49	9.55
18.24	11.41	16.83	7.38	18.75	13.79	9.77
17.57	11.07	16.22	6.69	16.94	13.20	8.52
17.10	9.98	15.90	6.84	13.22	13.16	7.59
17.74	10.42	16.21	7.07	13.58	13.38	7.71
18.85	11.05	16.09	6.93	13.71	13.26	7.82
18.60	10.92	16.01	6.76	13.99	13.04	7.89
16.46	10.47	16.58	6.53	14.92	13.51	7.62
16.25	9.91	16.03	6.15	14.12	12.52	7.13
16.39	9.96	15.35	6.24	14.33	12.46	6.96
16.50	9.99	15.40	6.86	14.33	12.79	8.07
16.84	10.76	16.13	7.31	17.16	13.61	8.77
16.83	10.71	16.77	7.43	18.88	13.77	9.30
15.16	9.47	15.29	6.48	17.77	12.44	8.08
15.11	9.96	15.69	6.65	15.63	12.64	7.71
17.43	10.60	18.24	7.46	22.26	14.78	11.92
18.35	10.26	18.88	8.20	22.09	15.29	11.92
17.50	9.85	16.91	7.38	22.26	13.89	12.34
16.66	9.74	16.35	7.03	21.18	13.45	11.44
18.30	10.80	17.81	8.48	21.53	14.99	11.21
17.49	10.35	16.16	7.47	21.53	13.39	11.21
16.19	10.01	16.31	6.57	20.21	13.41	10.50
16.19	10.01	16.31	6.57	20.21	13.41	10.50
16.97	10.79	16.74	8.68	23.60	15.28	14.94
16.29	10.68	16.95	8.29	23.60	14.80	13.95
17.97	10.43	16.09	6.87	19.88	13.63	10.96
16.35	9.94	16.10	6.49	19.31	13.10	10.60
15.72	11.69	16.68	6.96	15.17	13.44	8.08
14.67	10.63	15.60	6.16	14.27	12.54	7.26
15.72	9.71	15.21	5.68	14.14	12.41	6.83
15.32	9.58	15.14	5.64	14.89	12.29	6.98
18.02	10.23	16.02	6.43	16.89	12.64	7.67
17.82	10.38	16.33	6.70	18.46	13.08	8.80
15.28	11.80	17.01	6.73	19.99	13.94	11.17
15.28	11.80	17.01	6.73	19.99	13.94	11.17
17.44	11.84	17.12	8.26	20.34	13.89	11.09
17.18	11.71	17.05	8.16	19.26	13.91	10.75
17.14	10.69	16.24	7.23	19.19	13.36	9.64
18.21	10.87	16.62	7.83	20.35	13.90	10.67
17.63	10.52	16.85	7.78	20.06	13.59	10.91
17.20	10.50	17.16	8.10	21.79	14.17	11.85
18.10	11.15	17.67	8.38	23.93	14.78	12.70
18.57	11.02	17.92	8.77	22.95	14.57	12.86
18.88	11.06	17.80	7.71	23.52	14.29	12.46
17.75	10.60	16.67	7.11	24.36	13.62	11.50
18.79	12.04	17.50	8.93	22.47	14.58	13.11
19.64	11.80	17.62	8.62	23.01	14.35	12.60
