# Supplementary material for: The expression signatures in liver and adipose tissue from obese Göttingen Minipigs reveal a predisposition for healthy fat accumulation
Source: Nutr Diabetes. 2020 Mar 23;10:9. doi: 10.1038/s41387-020-0112-y (PMC7090036; doi:10.1038/s41387-020-0112-y)
Supplement: Supplementary file 5 — S4 Table [file 41387_2020_112_MOESM5_ESM.pdf]

S4. Raw qPCR data from VAT

Samples	ABCA1	ABCG1	ACACA	ADCY5	ADIPOQ	ADM	ADRBK2	AKT2	ANGPTL4	AQP7	CD40	CLU	COL6A1	CXCL14	CXCR4	DGAT2	DICER1	DUSP10	EBF2	ELOVL4	ELOVL6	FADS1	FAS	FABP4
221516.1	12.70	15.56	12.78	15.80	8.67	15.16	15.48	12.31	12.89	14.92	15.35	12.38	10.74	15.85	16.56	12.17	13.28	16.63	15.73	16.26	10.94	16.04	16.23	6.65
221516.2	13.21	16.13	13.56	15.91	9.16	15.45	16.14	12.62	13.56	15.30	15.91	12.61	11.15	15.81	16.81	12.40	13.38	16.68	16.00	16.06	11.12	16.53	16.03	6.73
319021.1	12.66	15.62	12.30	16.50	8.53	14.58	15.55	11.98	13.76	13.93	15.23	11.83	9.98	13.19	16.70	10.33	12.94	15.17	15.30	15.05	10.04	15.36	15.45	6.87
319021.2	13.31	16.17	12.91	16.68	9.03	15.19	16.03	12.35	14.31	14.40	15.68	12.13	10.50	13.53	17.16	10.63	13.33	15.61	15.37	15.34	10.26	15.64	15.92	7.13
319357.1	12.68	15.74	11.69	15.05	6.68	13.29	14.54	11.60	12.63	12.95	16.31	15.15	10.34	14.25	17.58	8.93	12.80	12.90	15.75	18.73	7.72	18.22	14.89	5.22
319357.2	12.49	15.54	11.39	14.96	6.39	12.94	14.36	11.38	12.48	12.78	16.08	14.78	10.11	14.14	17.78	8.58	12.58	12.75	15.65	18.43	7.62	17.46	14.77	5.01
319677.1	15.38	18.08	13.95	18.92	10.34	16.40	17.62	14.44	16.87	15.94	17.34	15.48	13.13	18.19	18.58	12.56	15.32	17.37	18.09	17.89	11.82	19.39	17.22	8.91
319677.2	15.08	17.74	13.49	18.45	10.04	15.77	17.22	14.11	16.28	15.77	16.95	15.25	12.84	17.85	18.32	12.12	14.90	17.19	17.32	18.25	11.48	18.85	17.54	8.62
319703.1	12.49	15.55	10.63	15.62	7.42	13.92	14.42	11.71	13.68	12.82	15.46	12.73	10.36	14.08	16.26	8.70	12.63	13.96	14.70	14.86	7.96	17.17	15.53	5.55
319703.2	12.66	15.71	10.65	15.78	7.52	13.95	14.49	11.79	13.75	12.80	15.63	12.77	10.44	14.16	16.50	8.77	12.82	13.98	14.95	15.12	8.12	17.28	15.69	5.67
319876.1	16.02	18.71	14.17	18.44	9.80	15.98	18.01	14.27	15.20	15.49	18.82	15.27	14.03	18.21	18.67	12.88	15.22	16.93	17.91	19.30	12.08	20.52	17.86	8.14
319876.2	16.09	18.70	14.28	18.17	9.77	16.28	17.96	14.20	14.94	15.64	18.16	14.91	14.11	18.62	19.69	12.88	15.06	17.43	18.04	19.21	11.87	20.87	17.74	8.03
320462.1	12.43	15.42	11.42	15.36	7.39	14.27	13.50	11.66	12.53	13.01	14.72	12.16	11.12	13.41	14.47	9.55	12.66	14.83	15.12	15.06	8.81	16.61	15.07	4.74
320462.2	12.36	15.60	11.32	15.21	7.44	14.06	13.42	11.62	12.54	13.07	14.49	12.21	11.20	13.53	14.43	9.75	12.56	14.95	15.14	15.27	8.87	16.90	15.09	4.81
221497.1	13.14	16.92	12.58	16.67	8.53	14.60	15.49	12.36	12.60	13.89	15.84	12.04	10.43	14.91	14.63	9.98	13.22	15.33	14.83	15.77	9.53	17.79	16.29	6.28
221497.2	13.04	16.75	12.50	17.27	8.63	14.68	15.27	12.46	12.67	14.00	15.93	12.21	10.43	15.51	15.13	10.17	13.46	15.23	15.32	16.20	9.70	17.72	16.50	6.68
221510.1	12.59	15.67	10.67	15.70	7.26	13.51	14.89	11.39	12.03	12.81	14.73	12.60	10.38	14.23	15.50	7.96	12.40	14.03	14.93	15.41	7.82	17.03	15.17	5.27
221510.2	12.77	15.90	10.76	15.51	7.23	13.73	15.07	11.47	12.14	12.70	14.67	12.65	10.45	14.24	15.58	8.01	12.38	13.93	14.70	15.63	7.92	16.86	15.24	5.28
221538.1	12.85	16.24	12.81	16.43	8.09	14.09	14.87	12.41	12.53	13.62	15.03	12.04	10.32	14.29	15.62	11.26	13.19	14.63	15.43	15.25	10.15	16.25	15.90	6.20
221538.2	12.73	16.19	12.62	16.45	8.05	14.14	15.11	12.33	12.51	13.46	14.86	11.95	10.18	14.22	15.74	11.20	13.16	14.62	15.28	14.93	10.04	16.30	16.10	6.07
319035.1	14.04	17.99	12.03	15.98	7.34	14.11	15.39	11.86	13.09	12.58	15.92	13.42	11.80	14.73	16.53	9.81	12.96	14.69	15.72	15.93	9.10	16.80	15.45	5.19
319035.2	14.04	17.99	12.03	15.98	7.34	14.11	15.39	11.86	13.09	12.58	15.92	13.42	11.80	14.73	16.53	9.81	12.96	14.69	15.72	15.93	9.10	16.80	15.45	5.19
319239.1	13.54	17.16	13.46	17.09	9.10	14.50	16.36	13.21	13.72	14.60	15.88	13.18	11.51	15.53	17.22	11.81	13.73	15.23	16.07	16.10	10.93	18.26	16.08	7.07
319239.2	13.08	16.24	13.09	17.21	8.58	13.85	15.58	12.71	13.13	14.29	15.59	12.71	10.89	15.16	16.47	11.28	13.13	14.48	15.31	15.69	10.33	17.55	15.41	6.49
319389.1	10.42	13.14	11.67	13.44	8.57	12.77	12.85	10.38	11.11	14.12	12.17	8.50	8.19	11.15	14.82	10.45	10.86	13.65	13.00	13.10	9.80	12.36	13.44	5.36
319389.2	9.98	12.80	11.42	12.95	8.10	12.42	12.59	9.82	10.83	13.68	11.88	8.29	8.04	10.92	14.44	10.00	10.10	13.21	12.53	12.57	9.31	11.93	13.10	5.27
319798.1	13.28	16.34	13.33	16.99	9.28	14.57	16.20	12.58	13.59	15.19	15.25	12.04	11.05	15.97	15.47	11.30	13.18	15.73	15.80	16.74	9.97	17.29	16.06	6.96
319798.2	13.17	16.24	13.09	17.12	9.11	14.18	15.67	12.49	13.53	15.00	15.14	11.84	10.87	15.84	15.32	11.19	13.15	15.53	15.89	16.15	9.80	17.05	16.09	6.85
319799.1	15.47	18.74	15.86	19.72	11.45	16.30	18.43	14.85	15.82	18.08	17.29	15.51	13.65	17.39	18.55	13.11	15.88	17.88	18.07	17.98	13.06	19.68	18.12	8.88
319799.2	15.78	18.65	15.87	19.36	11.86	16.50	18.42	15.17	16.12	17.78	19.06	16.02	13.84	17.98	18.71	13.47	16.27	17.87	18.02	18.35	13.42	20.64	18.17	9.01
319875.1	15.04	18.59	14.61	19.32	10.30	16.48	18.11	14.77	14.16	16.56	18.81	15.09	13.25	16.94	19.16	13.60	16.11	17.43	18.69	19.37	12.72	19.33	18.94	9.06
319875.2	15.33	19.22	14.94	19.08	10.24	16.41	17.96	14.57	14.43	16.63	18.03	14.68	13.58	17.22	19.10	13.19	15.72	16.79	17.77	18.33	12.23	19.45	18.61	8.73
320241.1	13.06	15.84	12.68	16.37	9.51	14.87	15.17	12.34	13.58	14.65	15.09	10.88	10.32	12.31	16.21	10.67	13.22	15.49	15.15	15.57	9.33	15.60	16.03	6.91
320241.2	12.90	15.66	12.68	16.47	9.27	14.71	15.01	12.34	13.51	14.65	15.31	10.74	10.10	12.25	16.29	10.60	13.06	15.36	15.35	15.35	9.12	15.42	15.82	6.71
320316.1	12.64	15.72	13.30	15.35	8.82	14.00	15.24	12.10	12.83	14.16	14.71	11.61	9.39	14.33	16.23	11.18	12.52	15.13	14.62	14.66	10.48	14.78	15.73	6.52
320316.2	12.56	15.98	13.24	15.25	8.81	13.96	14.88	11.95	12.62	14.12	14.55	11.08	9.40	13.88	16.14	11.09	12.31	14.81	14.16	14.79	10.33	14.76	15.52	6.36
320317.1	13.42	16.29	12.13	15.66	7.82	13.33	14.73	11.57	12.60	12.73	14.50	12.77	10.69	14.87	16.38	9.44	12.54	13.85	15.04	15.96	9.12	16.82	14.26	5.08
320317.2	13.09	16.22	11.86	15.60	7.47	13.23	14.33	11.30	12.30	12.32	14.18	12.27	10.52	14.75	16.00	9.29	12.26	13.62	14.53	16.12	8.87	16.95	13.93	4.87
320351.1	13.55	16.29	10.73	16.96	7.27	13.91	14.70	11.74	14.47	12.40	15.57	12.96	11.19	14.71	16.29	9.24	12.95	14.75	15.19	16.02	8.02	17.26	15.10	5.64
320351.2	13.33	16.60	10.42	16.50	7.21	13.83	14.45	11.58	14.38	12.18	15.88	12.76	11.01	14.64	16.19	9.04	12.76	14.67	15.19	15.95	7.88	16.91	15.09	5.42
320440.1	13.08	16.43	12.26	16.25	7.04	13.51	15.07	11.61	12.19	12.39	15.42	12.58	10.82	14.38	15.71	9.59	12.75	14.23	15.17	15.50	8.98	17.25	14.97	5.48
320440.2	13.17	16.39	12.31	16.02	7.29	13.78	15.23	11.83	12.27	12.50	15.35	12.55	10.81	14.37	15.84	9.68	12.77	14.25	15.22	15.53	8.96	17.20	14.90	5.33
319427.1	15.69	19.08	12.51	16.49	7.97	14.51	15.72	12.27	13.80	14.67	16.79	15.96	11.89	15.98	16.60	11.15	13.87	13.68	16.59	17.01	8.22	17.62	15.22	6.07
319427.2	14.78	17.90	11.82	15.92	7.37	14.02	15.09	11.75	13.18	13.69	16.20	15.21	11.26	15.55	16.34	10.40	13.23	13.09	15.80	16.71	7.71	16.71	14.39	5.69
319778.1	14.44	17.78	12.59	16.92	8.51	15.45	15.52	12.65	13.56	14.72	15.98	12.60	11.29	14.32	15.99	10.63	13.57	15.41	16.35	15.74	10.51	16.67	15.84	6.81
319778.2	14.72	17.71	13.14	17.43	8.92	15.90	15.94	13.02	13.90	15.23	16.11	12.89	11.56	14.93	16.28	10.89	14.02	15.63	16.22	16.01	10.83	16.62	15.75	7.21
319922.1	14.21	17.95	11.72	17.31	6.86	14.06	14.91	11.40	12.71	13.49	16.23	14.11	11.82	15.11	17.50	8.68	12.92	13.63	15.23	16.92	8.71	16.92	15.78	5.72
319922.2	14.43	18.57	11.74	17.43	6.85	14.27	15.27	11.58	12.77	13.32	16.25	14.21	11.88	15.40	18.00	8.49	12.91	13.71	15.38	16.69	8.76	17.52	15.83	5.76
320207.1	14.56	16.48	11.02	16.19	8.03	15.30	15.35	11.86	15.16	13.74	15.79	13.38	10.93	14.56	17.09	9.07	13.06	14.66	15.65	16.71	8.74	16.04	14.88	6.17
320207.2	15.27	17.05	11.57	16.71	8.62	15.62	16.06	12.22	15.79	14.29	15.77	13.80	11.58	14.97	17.59	9.58	13.41	15.26	16.12	17.11	9.04	16.66	15.24	6.59
320411.1	13.38	16.22	13.79	16.85	9.99	14.39	15.99	12.43	13.93	16.08	15.10	11.80	10.29	15.23	16.20	12.02	12.36	14.94	14.86	15.22	10.85	14.44	15.17	7.72
320411.2	13.38	16.22	13.79	16.85	9.99	14.39	15.99	12.43	13.93	16.08	15.10	11.80	10.29	15.23	16.20	12.02	12.36	14.94	14.86	15.22	10.85	14.44	15.17	7.72

raw qPCR data efficiency corrected

ref genes

FGF21	IL6	GCLM	GLUT4	GNAS	GPC4	GRB10	ICAM-1	IDS	NR3CI	IRS1	IRX3	ISLR	JAG1	KLB	.CN2 (NGAL	LDLR	LEP	LEPR	LITAF	LPL	LSS	MGMT	MKL1	MOCOS
17.14	17.14	14.96	16.45	10.17	14.20	14.00	15.76	12.64	12.65	19.90	19.19	13.88	11.72	13.35	13.66	16.17	13.78	19.04	13.27	10.48	17.09	15.15	15.59	17.58
18.01	16.73	14.89	16.79	10.33	14.48	14.08	16.25	13.26	12.90	20.66	19.51	14.46	12.01	13.56	13.87	16.60	13.89	19.04	13.46	10.43	17.13	15.51	16.42	17.38
17.66	18.32	14.99	15.11	9.51	13.61	13.74	15.52	11.65	11.79	19.77	18.02	13.18	12.42	12.62	13.56	16.64	12.65	17.09	12.84	9.51	15.56	14.90	15.66	16.78
17.71	18.82	15.09	15.54	9.92	13.89	14.17	15.86	12.22	12.15	20.41	18.57	13.86	12.80	12.83	13.96	17.29	12.96	15.77	13.14	9.82	16.00	15.36	16.42	17.06
17.81	18.56	13.80	14.26	9.76	14.16	12.76	15.67	10.65	11.78	19.90	15.25	14.56	12.43	11.48	14.53	15.90	10.44	18.78	12.64	8.05	13.58	14.46	16.83	15.51
17.90	18.14	13.67	14.10	9.48	14.24	12.37	15.54	10.47	11.66	19.68	15.09	14.17	12.15	11.21	14.38	15.62	10.24	18.84	12.33	7.91	13.29	14.45	16.54	15.32
19.19	19.07	16.94	18.50	12.13	16.30	15.85	17.45	14.95	14.42	21.94	23.65	16.31	14.81	14.65	14.82	18.84	13.36	20.28	15.16	11.49	17.06	17.29	18.91	18.58
18.96	19.13	16.09	18.04	11.82	15.44	15.73	17.19	14.20	13.73	21.73	22.59	15.94	14.20	14.17	14.45	18.25	12.78	19.85	14.79	11.10	16.66	16.97	18.49	18.63
18.76	17.10	13.67	14.32	9.16	14.05	13.49	15.32	11.41	11.55	19.92	17.59	13.21	12.11	11.21	14.16	16.53	11.06	16.38	12.24	8.40	14.15	14.45	16.18	15.23
17.59	17.40	13.80	14.63	9.37	14.10	13.67	15.31	11.53	11.75	19.77	17.64	13.42	12.28	11.44	14.23	16.64	11.12	16.60	12.31	8.42	14.19	14.55	16.22	15.23
19.82	18.64	15.78	18.68	12.52	16.83	16.39	17.45	13.58	14.61	22.37	22.01	16.92	14.81	14.17	16.34	17.93	13.16	19.64	14.81	10.76	17.89	17.72	19.00	18.73
19.82	18.36	15.59	18.23	12.21	16.74	16.26	17.22	13.83	14.40	21.62	22.01	16.61	15.10	14.14	16.57	17.73	13.03	19.64	14.72	10.71	18.03	17.71	19.74	17.74
18.16	17.88	14.04	15.36	9.14	13.85	13.85	15.44	12.02	11.67	19.58	19.38	14.25	12.50	11.04	13.55	15.01	11.34	19.12	11.45	7.88	14.86	14.16	15.87	15.19
19.37	17.99	14.08	15.28	9.08	13.98	13.93	15.96	12.18	11.72	19.02	19.66	14.23	12.42	11.13	13.45	15.02	11.40	19.05	11.52	7.88	14.49	14.38	15.67	15.43
18.15	18.28	14.11	16.34	10.06	14.58	14.16	15.78	11.99	11.99	19.69	18.31	13.63	12.74	12.53	14.16	17.16	11.73	16.10	12.41	9.49	15.96	15.16	16.42	16.15
18.88	18.68	14.57	16.51	10.28	14.59	14.39	16.31	12.03	12.22	19.83	18.46	13.46	12.89	12.81	14.30	16.96	11.71	16.10	12.72	9.73	16.12	15.35	16.50	16.60
17.97	17.14	12.74	13.88	9.12	14.04	13.79	15.46	11.61	11.43	19.44	19.19	13.43	12.12	10.86	14.59	15.92	10.36	16.42	11.72	7.68	13.85	14.15	16.31	15.37
18.59	17.57	12.80	14.03	9.25	14.24	13.74	15.52	11.54	11.48	20.25	18.67	13.48	12.20	10.83	14.63	15.77	10.44	16.55	11.73	7.51	13.67	14.39	16.08	15.31
17.37	18.80	14.41	16.81	9.99	14.16	13.67	15.65	12.51	11.72	20.79	18.53	13.15	12.34	12.91	12.94	15.47	11.50	16.53	12.49	9.58	15.37	14.88	16.05	15.57
18.46	18.47	14.31	16.40	9.87	14.30	13.68	15.44	12.38	11.66	20.42	17.98	13.17	12.14	12.84	12.90	15.55	11.53	16.42	12.45	9.55	15.27	14.91	15.89	15.48
18.83	17.74	12.77	15.19	10.03	15.01	14.25	16.20	12.07	12.06	21.41	19.30	15.16	12.55	11.39	14.53	16.12	9.51	17.26	12.31	8.56	14.68	14.98	17.52	14.90
18.83	17.74	12.77	15.19	10.03	15.01	14.25	16.20	12.07	12.06	21.41	19.30	15.16	12.55	11.39	14.53	16.12	9.51	17.26	12.31	8.56	14.68	14.98	17.52	14.90
19.19	19.35	15.08	17.25	10.53	14.71	14.85	16.16	13.17	12.57	21.51	20.45	15.30	13.48	13.18	16.20	17.26	12.81	17.31	13.27	10.05	16.59	15.79	17.35	16.74
18.64	18.70	14.42	16.71	9.94	14.30	14.15	15.71	12.75	12.07	20.93	19.24	14.73	12.97	12.84	15.33	16.89	12.33	16.87	12.69	9.38	15.94	15.20	16.86	16.20
14.39	16.20	13.99	14.01	7.50	12.18	11.08	12.97	10.92	10.07	17.28	16.01	10.78	9.01	12.96	11.28	14.57	12.41	16.10	9.99	8.75	14.44	12.55	12.80	14.91
14.09	16.07	13.43	13.56	7.05	11.50	10.68	12.51	10.64	9.59	16.80	15.63	10.64	8.57	12.44	11.12	14.18	11.82	14.02	9.67	8.24	13.84	12.37	12.33	14.23
17.66	18.27	14.75	15.50	10.24	14.02	14.16	15.50	13.01	12.26	20.67	20.92	14.16	12.70	13.10	10.87	16.27	12.94	16.24	12.84	9.97	16.44	15.33	17.21	16.89
17.89	17.86	14.50	15.43	10.09	13.77	13.70	15.24	12.87	11.95	20.01	20.10	13.90	12.53	13.03	10.68	16.02	12.74	16.38	12.63	9.79	16.13	15.17	16.59	17.00
20.43	20.11	16.83	18.31	12.73	17.02	16.61	19.09	14.83	14.32	24.78	20.27	16.45	15.77	15.51	18.88	18.97	14.78	19.44	15.33	11.59	18.63	17.85	19.86	19.23
18.86	19.70	17.07	18.25	12.98	16.77	17.22	18.56	15.19	14.70	25.66	20.27	16.56	15.62	15.84	19.11	18.52	15.18	19.94	15.72	11.86	18.63	18.10	19.79	18.35
19.89	20.69	17.99	18.99	12.87	16.77	16.50	18.60	14.48	14.83	23.60	19.90	15.81	15.23	15.69	17.90	18.22	14.14	18.40	15.72	11.88	18.29	17.75	18.50	19.72
18.98	20.38	17.52	18.72	12.38	16.85	16.34	18.44	15.15	14.56	22.99	19.90	16.04	15.08	14.79	17.59	19.60	14.16	18.40	15.01	11.65	18.19	17.73	18.63	18.26
16.78	19.58	14.85	14.61	9.60	14.41	13.85	16.19	12.28	12.15	20.22	19.09	13.49	12.80	13.37	13.95	16.92	12.63	16.13	12.58	9.55	15.23	14.94	15.51	16.41
17.08	19.50	14.75	14.53	9.57	14.28	13.86	16.27	12.12	12.09	20.02	18.78	13.56	12.78	13.32	13.84	16.57	12.44	16.06	12.45	9.36	14.78	14.83	15.24	16.06
16.77	18.02	14.59	15.08	9.35	13.63	13.04	15.12	12.22	11.50	19.76	17.88	12.72	11.20	13.60	13.46	15.45	11.67	15.07	12.28	10.15	15.61	14.80	15.33	15.92
16.54	17.82	14.27	15.20	9.16	13.51	12.79	14.97	12.14	11.36	19.44	17.77	12.84	11.24	13.37	13.07	15.56	11.75	15.04	11.85	10.03	15.37	14.57	14.99	15.92
17.57	16.72	12.68	15.03	9.31	13.48	13.74	13.21	11.73	11.56	20.33	17.96	14.58	12.09	11.01	12.37	15.15	10.07	17.84	11.63	8.66	15.10	14.55	16.54	14.82
18.19	16.15	12.44	14.60	8.98	13.33	13.35	13.06	11.61	11.22	20.04	17.84	14.80	11.90	10.63	12.23	15.04	9.66	16.84	11.40	8.38	14.87	14.27	16.01	14.67
18.33	20.73	13.11	14.94	9.52	14.61	14.17	16.52	10.96	11.75	20.11	18.58	14.23	12.54	11.33	14.11	17.02	9.64	15.48	12.16	7.84	13.91	14.65	16.62	14.31
18.33	19.33	12.92	14.57	9.37	14.49	14.07	16.66	10.68	11.55	19.50	18.30	14.01	12.28	11.18	13.95	16.74	9.39	15.50	11.93	7.84	13.75	14.89	16.39	14.31
17.87	17.36	13.47	16.33	9.49	14.14	13.85	16.02	11.27	11.83	20.38	18.19	14.15	12.34	11.64	13.16	16.38	10.74	15.29	12.10	8.54	14.65	15.06	16.88	15.13
18.09	17.67	13.36	16.53	9.57	13.84	13.80	15.69	11.26	11.79	20.28	18.09	14.34	12.22	11.56	13.36	16.52	10.67	15.00	12.08	8.52	14.64	14.98	16.71	15.19
18.53	17.94	14.87	14.98	10.70	15.85	14.96	16.44	12.60	12.68	21.78	16.32	15.63	12.90	12.25	16.18	15.30	11.33	18.33	13.81	8.95	14.26	16.02	17.45	16.13
18.53	17.32	14.37	14.51	9.93	14.94	14.35	15.70	11.85	12.15	20.61	15.81	15.06	12.34	11.54	15.97	14.74	10.75	17.60	12.97	8.24	13.72	14.86	17.13	15.52
18.59	19.32	15.44	16.23	10.28	14.63	14.25	16.12	13.04	12.35	21.26	18.14	14.16	12.69	12.94	14.65	16.08	13.12	15.53	12.97	9.17	15.53	15.56	16.92	16.14
19.02	20.50	15.38	16.70	10.65	15.07	14.60	16.44	13.48	12.71	21.95	18.60	14.49	13.16	13.59	15.17	16.71	13.51	15.97	13.60	9.66	16.01	16.06	17.34	16.69
17.32	21.77	14.51	14.53	9.47	14.59	14.02	15.60	11.55	12.11	19.58	18.95	14.35	12.21	10.82	12.37	15.19	12.97	16.15	13.05	7.57	14.11	14.83	17.08	15.63
17.26	21.77	14.65	14.56	9.60	14.66	14.02	15.68	11.70	12.27	20.03	19.42	14.40	12.34	10.86	12.39	15.19	12.99	16.04	12.92	7.52	14.38	14.91	16.98	15.40
18.18	19.48	15.04	14.69	9.71	15.12	14.02	15.81	11.62	12.34	21.09	19.59	14.55	12.24	12.05	13.69	15.41	12.25	17.40	12.52	7.84	14.61	15.31	16.33	16.25
18.41	19.84	15.48	15.33	10.12	15.49	14.23	16.33	12.14	12.86	21.11	20.11	15.17	12.76	12.61	14.23	15.98	12.82	17.66	13.22	8.20	14.92	15.82	16.88	16.99
17.38	17.79	14.92	16.31	9.43	13.11	13.11	14.92	12.88	11.54	20.38	18.82	13.17	12.12	14.87	11.90	15.28	14.71	13.60	12.70	10.49	15.40	15.10	16.06	16.91
17.38	17.79	14.92	16.31	9.43	13.11	13.11	14.92	12.88	11.54	20.38	18.82	13.17	12.12	14.87	11.90	15.28	14.71	13.60	12.70	10.49	15.40	15.10	16.06	16.91

MTOR	MYC	NCOR2	NEGR1	NFKBIA	NPEPL1	OSBPL10	TLR4	PEG10	PELI2	PN-1	PNPLA2	PON1	PPARD	PRKG1	RDH5	RHOQ	RNF10	RORA	RPS29	SAA	40057.00	SMAD6	SMPDL3A	SP1
15.71	15.28	15.01	23.13	11.85	15.88	15.86	15.03	17.29	14.37	14.84	11.85	16.53	14.90	15.49	18.51	12.83	12.11	16.08	12.92	20.20	20.36	16.03	11.41	13.64
15.65	16.05	14.85	25.00	11.99	16.49	15.77	14.92	17.08	15.17	15.00	12.04	16.60	14.82	15.97	19.47	13.17	12.26	15.48	11.70	19.97	21.31	16.09	11.53	13.83
15.23	15.10	14.46	17.13	11.69	15.35	15.55	14.88	16.34	14.41	14.96	12.07	16.03	14.57	14.23	16.40	12.19	11.57	14.85	11.57	20.43	18.88	16.03	11.43	13.00
15.71	15.77	14.75	17.18	12.01	15.82	15.76	15.12	16.34	14.91	15.40	12.55	16.30	14.94	14.36	16.68	12.45	11.88	15.01	11.57	20.19	20.10	16.26	11.70	13.44
15.46	16.33	14.42	18.02	12.33	15.03	16.33	15.20	16.27	16.34	14.90	10.85	13.96	14.15	17.13	13.82	11.35	11.75	14.64	11.33	20.14	15.68	15.49	8.87	13.59
14.97	15.93	14.21	17.26	11.98	15.05	16.43	15.22	15.70	16.03	14.56	10.86	13.65	14.18	16.54	13.58	11.07	11.54	14.46	11.05	19.47	15.83	15.14	8.73	13.51
18.34	18.18	17.45	20.59	14.40	17.68	18.34	17.84	19.35	17.11	16.54	14.59	16.86	17.08	17.77	17.54	14.32	14.66	17.51	14.23	22.28	19.61	17.79	13.07	15.63
17.92	17.68	17.11	19.70	14.01	17.69	17.50	17.17	18.29	16.95	16.14	14.43	16.56	16.89	17.60	17.17	14.07	13.98	16.55	13.56	22.62	19.69	17.53	12.75	15.24
15.09	15.15	14.57	21.27	11.26	15.33	15.98	14.23	17.15	15.68	13.73	10.83	14.58	13.93	15.64	15.20	10.93	11.42	14.29	10.83	19.96	17.56	15.64	9.93	13.16
15.27	15.27	14.62	21.34	11.43	15.63	16.07	14.56	16.63	15.70	14.11	11.00	14.78	14.37	16.10	15.06	11.07	11.45	14.33	10.83	19.30	17.41	15.74	10.07	13.13
18.11	18.15	16.70	18.96	14.39	18.19	18.43	16.67	18.41	17.81	16.75	13.99	16.78	16.46	18.07	18.68	14.38	14.21	16.91	14.25	23.21	20.89	16.95	12.98	16.07
18.08	18.14	17.57	18.27	14.44	18.80	18.59	16.81	19.11	17.32	16.35	14.08	17.01	16.60	17.90	18.32	14.08	14.12	17.46	14.06	23.95	20.74	16.76	12.80	15.70
14.90	14.98	13.92	19.72	11.60	15.18	16.84	12.85	19.43	15.76	12.51	10.74	14.25	13.01	15.42	15.16	11.27	11.33	14.97	10.57	19.06	17.45	15.43	9.25	13.08
14.91	14.67	13.96	19.19	11.75	15.29	16.41	12.93	17.82	15.77	12.79	10.95	14.46	12.78	15.76	15.10	11.17	11.30	14.80	10.25	19.27	17.68	15.34	9.30	13.04
15.67	15.05	15.06	23.78	11.86	16.54	16.81	14.11	17.12	15.41	15.11	11.91	16.33	15.57	15.67	16.67	11.83	12.15	15.00	11.13	20.86	18.56	16.20	10.62	13.45
15.84	15.16	15.24	23.78	12.08	16.44	17.07	14.32	18.44	15.53	15.08	11.75	17.06	15.90	16.08	16.82	12.00	12.24	15.12	12.10	20.51	18.24	16.49	10.87	13.74
15.02	14.43	14.36	21.22	11.37	14.88	16.05	13.70	18.35	15.37	13.09	10.47	14.49	13.34	14.87	13.45	10.92	10.94	14.34	10.11	20.28	16.69	16.03	9.64	13.12
15.10	14.61	14.46	21.18	11.55	14.82	16.20	13.86	18.61	15.58	13.20	10.59	14.44	13.40	15.37	13.50	10.96	11.03	14.46	10.28	20.04	16.55	15.98	9.73	13.07
15.56	15.57	14.43	22.99	11.62	15.80	16.07	14.22	17.30	15.19	15.46	11.61	16.57	14.27	15.53	16.65	12.09	12.12	14.83	11.32	20.19	18.95	16.41	10.51	13.24
15.64	15.52	14.39	22.38	11.64	15.77	16.01	14.18	17.08	15.19	15.05	11.51	16.62	14.13	15.52	16.14	12.01	12.09	14.93	11.15	20.53	18.49	16.10	10.45	13.35
15.96	16.04	15.06	20.92	11.94	15.55	16.80	14.89	19.90	15.93	13.28	11.29	15.23	14.05	16.99	14.93	11.00	11.42	14.66	10.87	19.75	17.87	15.96	9.56	14.14
15.96	16.04	15.06	20.92	11.94	15.55	16.80	14.89	19.90	15.93	13.28	11.29	15.23	14.05	16.99	14.93	11.00	11.42	14.66	10.87	19.75	17.87	15.96	9.56	14.14
16.53	16.81	15.52	21.94	12.76	16.78	17.30	15.33	17.24	16.10	16.05	12.64	16.91	15.95	16.38	17.04	12.91	10.94	15.25	11.29	20.62	19.74	16.29	11.54	14.30
16.01	15.73	15.05	21.25	12.24	16.02	16.64	14.63	17.24	15.61	15.55	12.11	16.34	15.06	15.54	16.45	12.24	10.46	14.81	10.78	19.84	19.01	16.28	11.03	13.73
12.41	12.87	11.86	18.34	8.78	13.77	13.33	12.62	14.26	12.33	11.65	10.31	15.63	12.47	12.34	16.82	10.23	9.49	12.16	9.11	17.50	18.10	12.67	9.33	11.00
11.97	12.66	11.09	17.44	8.44	13.01	12.58	12.19	13.99	12.03	11.07	9.89	14.85	12.14	11.85	16.21	9.77	9.22	11.66	8.29	16.94	17.90	11.81	8.93	10.51
16.09	15.53	14.85	20.04	11.84	15.91	15.84	14.85	16.80	15.24	15.79	12.04	16.15	14.47	15.18	18.22	12.82	12.34	15.20	10.70	20.13	19.89	16.05	11.26	13.62
15.96	15.48	14.74	20.20	11.76	15.65	15.65	14.88	16.80	14.88	15.37	11.95	16.01	14.16	15.04	17.75	12.57	12.29	15.19	10.57	20.38	19.67	15.81	11.11	13.43
19.00	18.64	17.13	23.80	14.27	18.05	19.10	17.20	19.81	18.01	18.94	14.79	20.11	17.09	18.50	19.99	15.17	15.03	17.18	13.55	23.03	22.79	18.62	12.98	16.59
19.49	18.70	17.45	23.80	14.53	18.58	18.76	17.49	19.81	18.65	19.11	15.13	19.87	17.64	18.51	19.92	15.51	15.37	17.95	13.81	21.06	21.02	19.25	13.31	16.79
18.30	18.19	17.20	22.44	14.27	18.59	18.33	17.38	19.44	17.98	16.78	14.22	18.89	17.29	17.81	20.69	14.83	14.86	17.03	14.05	22.89	22.66	18.85	13.65	16.32
18.02	18.42	17.05	22.44	14.07	18.19	18.03	17.05	17.90	17.72	16.75	14.18	18.32	17.36	17.92	19.81	14.76	14.54	17.15	13.34	22.89	21.71	18.22	13.30	16.17
15.33	15.69	14.49	16.26	12.00	15.34	15.36	14.63	15.85	15.39	15.10	12.35	16.65	14.35	14.00	15.18	12.03	11.87	14.65	10.70	20.23	19.28	16.74	10.82	13.34
15.05	15.61	14.40	16.17	11.93	15.34	15.29	14.41	15.73	15.45	14.75	12.40	16.41	14.29	13.94	15.34	12.01	11.69	14.80	10.71	19.49	19.02	16.21	10.72	13.42
15.14	15.32	13.92	19.61	10.87	15.33	15.60	13.96	17.13	14.37	13.35	11.88	15.74	14.62	14.42	15.84	11.82	11.49	14.24	10.18	19.29	19.22	15.32	10.90	12.96
14.82	14.95	13.78	19.77	10.58	15.36	15.37	13.82	16.69	14.46	13.41	12.02	15.69	14.54	14.32	15.40	11.62	11.37	14.33	9.97	19.47	18.99	15.34	10.75	12.88
15.22	15.53	14.64	16.49	9.39	14.94	16.21	14.44	17.94	15.16	13.57	10.86	14.13	13.76	15.42	14.93	11.22	11.18	14.44	10.13	19.49	17.52	15.25	10.20	13.26
14.98	15.04	14.21	16.23	9.14	14.90	16.07	14.13	16.72	14.97	13.25	10.49	13.75	13.45	14.94	14.74	10.91	10.95	14.26	9.64	19.11	16.94	15.18	9.90	13.00
15.56	16.33	14.73	22.31	12.03	15.73	16.58	14.63	17.39	15.76	13.90	11.20	14.34	14.11	15.90	13.83	10.89	11.57	14.39	10.74	20.96	17.78	16.31	9.19	13.64
15.18	16.20	14.54	22.21	11.87	15.17	16.76	14.61	17.39	15.50	14.06	10.96	14.33	13.92	15.50	13.64	10.69	11.53	14.13	10.54	21.20	17.67	16.08	8.97	13.42
15.45	15.94	14.81	23.68	11.74	15.61	16.00	14.26	16.74	15.40	14.59	11.20	15.23	14.38	15.84	14.64	11.20	11.58	14.46	10.53	20.44	17.87	15.86	9.94	13.79
15.56	15.84	14.68	21.90	11.72	15.49	16.35	14.29	17.23	15.26	14.34	11.25	15.13	14.64	15.79	14.27	11.22	11.50	14.40	10.44	20.06	18.01	15.86	9.74	13.56
16.22	16.51	15.13	16.80	12.79	15.81	17.24	16.19	19.44	16.85	15.15	12.25	15.78	14.35	17.66	16.96	11.83	12.59	15.62	11.21	18.95	17.42	16.94	10.03	14.57
15.63	16.05	14.31	16.28	12.11	15.41	16.45	15.56	18.03	16.49	14.70	11.27	15.19	13.66	16.87	16.83	11.17	11.99	15.00	10.37	19.29	16.57	15.77	9.42	13.94
16.30	16.03	15.21	22.39	12.21	16.65	16.67	15.06	17.70	15.41	16.59	12.23	15.79	15.31	15.80	16.86	12.73	12.61	15.29	10.85	20.63	21.67	16.85	11.64	13.91
16.68	16.96	15.67	22.39	12.48	17.07	17.81	15.46	18.60	16.01	16.78	12.58	16.57	16.03	16.81	16.81	13.11	12.92	15.24	11.10	21.49	21.55	17.24	11.88	14.27
15.45	16.73	14.90	18.34	12.06	15.52	16.59	14.87	16.21	15.41	16.17	10.64	14.79	15.03	15.57	13.27	12.35	11.91	14.89	10.30	20.52	18.46	15.41	11.36	13.67
15.55	16.85	14.91	18.00	12.04	15.78	16.63	15.17	17.19	15.57	16.40	10.69	14.76	15.13	15.73	13.28	12.28	12.01	15.28	10.21	21.56	18.31	15.52	11.30	13.64
15.37	16.98	14.77	19.58	11.86	15.70	16.57	16.30	18.68	15.63	14.75	11.59	15.93	14.24	15.47	14.79	12.35	12.04	15.33	10.90	20.14	19.95	16.13	11.78	13.76
16.15	17.41	15.21	19.53	12.33	16.60	17.03	16.49	18.01	16.02	15.50	12.12	16.34	14.72	15.33	15.13	12.78	12.51	15.45	10.99	20.40	20.08	16.81	12.08	14.23
15.41	15.36	14.15	19.32	11.19	15.38	15.10	15.18	16.54	14.77	14.78	12.63	17.53	14.98	14.68	17.28	12.72	11.81	14.00	10.16	20.06	21.67	14.94	11.95	13.01
15.41	15.36	14.15	19.32	11.19	15.38	15.10	15.18	16.54	14.77	14.78	12.63	17.53	14.98	14.68	17.28	12.72	11.81	14.00	10.16	20.06	21.67	14.94	11.95	13.01

SREBP1	STIM1	SYNE1	TBC1D16	TECPR2	TGFB1	TGFB3	TNF	TNS3	USF1	ACTB	HPRT1	TBP	YWHAZ
14.98	14.16	13.96	17.10	15.90	13.92	16.55	18.21	13.36	17.48	9.06	17.22	16.03	10.95
16.40	14.20	14.11	17.57	16.26	14.41	16.99	18.26	13.52	17.43	9.16	15.22	16.54	10.86
15.31	13.38	13.27	16.91	16.18	13.60	16.04	18.39	13.38	16.68	8.45	15.92	15.53	10.03
16.21	13.57	13.68	17.45	16.14	14.14	16.59	18.70	13.67	17.68	8.95	15.68	15.69	10.32
16.24	13.37	15.25	16.36	16.27	14.92	17.12	18.49	12.87	16.55	9.76	14.85	15.36	9.37
16.05	13.35	15.19	16.28	15.84	14.68	16.97	17.92	12.58	16.70	9.67	14.84	15.35	9.09
18.06	16.26	16.23	19.17	18.63	15.47	19.25	19.34	15.63	19.01	11.98	18.32	18.34	12.52
18.12	15.73	16.16	18.74	18.34	15.36	18.51	19.43	15.06	19.01	11.51	17.73	17.80	12.09
15.88	13.34	13.86	16.68	15.62	13.56	17.25	18.23	12.72	17.03	9.05	14.79	15.35	9.57
16.04	13.42	14.05	16.72	15.82	13.55	17.33	18.32	12.80	16.95	9.11	15.07	15.65	9.77
19.00	16.27	16.94	19.47	18.30	15.73	20.18	20.60	15.66	19.03	12.10	17.77	18.21	12.63
19.09	16.42	16.58	19.67	18.45	16.27	20.27	19.48	15.62	19.88	12.07	17.27	18.67	12.32
15.60	12.88	14.45	16.37	15.69	12.03	18.06	18.23	11.97	16.55	8.29	12.97	15.00	8.87
15.50	13.00	14.20	16.35	15.69	12.15	18.24	17.70	12.03	16.72	8.26	12.99	15.20	8.90
16.43	13.53	13.94	17.85	16.53	14.06	17.18	22.00	13.71	17.38	9.44	15.33	15.90	10.25
15.77	13.47	14.20	17.69	16.71	13.99	16.71	20.82	13.87	17.75	9.49	15.86	16.10	10.52
16.34	13.22	14.00	15.96	15.70	13.52	17.10	16.91	12.10	16.72	8.38	13.70	15.31	8.90
16.46	13.20	13.88	16.16	15.85	13.17	16.72	16.85	12.19	17.11	8.50	13.88	15.55	9.06
15.72	13.16	13.66	16.99	15.67	13.00	16.42	17.62	13.06	17.04	9.45	14.58	15.48	10.26
15.63	13.19	13.77	16.83	15.68	12.82	16.56	17.36	13.05	17.45	9.19	14.61	15.44	10.23
16.87	13.73	14.35	16.99	16.71	13.92	18.78	18.35	12.78	17.70	9.32	14.11	15.88	9.80
16.87	13.73	14.35	16.99	16.71	13.92	18.78	18.35	12.78	17.70	9.32	14.11	15.88	9.80
17.01	14.27	15.18	18.02	16.54	14.37	18.23	17.69	13.77	17.56	10.60	15.42	16.69	11.26
16.81	13.69	14.48	17.35	16.26	13.86	17.44	17.59	13.23	17.29	9.91	14.84	16.30	10.56
12.88	11.09	11.16	14.43	14.11	10.87	12.48	14.43	11.73	14.30	5.94	13.05	13.34	8.14
13.21	10.43	10.51	13.68	13.48	10.80	11.94	14.01	11.05	13.95	5.30	12.18	12.91	7.28
16.76	13.92	14.25	17.21	16.30	14.04	17.27	17.80	12.87	17.40	9.64	14.81	15.79	10.24
16.41	13.63	14.07	17.14	16.19	13.81	17.21	18.05	12.77	17.42	9.46	14.44	15.74	9.99
18.76	15.70	17.23	19.07	19.03	16.06	20.17	19.26	15.67	19.06	12.17	16.27	18.66	13.26
19.07	16.44	17.04	20.81	18.34	16.52	19.46	20.93	15.90	20.36	12.41	16.92	18.98	13.48
18.08	16.27	17.21	19.39	18.13	15.59	20.26	20.37	16.20	20.55	12.22	19.07	19.46	12.89
19.14	15.87	16.19	19.54	18.43	16.06	19.96	21.06	16.13	18.60	12.27	17.52	19.33	12.46
16.87	13.09	13.63	16.49	16.39	13.27	16.97	17.95	13.82	16.52	8.45	14.65	15.88	9.81
16.53	13.16	13.49	16.71	16.16	13.09	17.04	17.89	13.56	16.43	8.29	14.67	15.42	9.70
15.13	12.81	13.27	16.38	15.68	13.23	15.66	15.48	13.27	17.12	8.30	14.39	15.20	9.46
15.63	12.89	12.87	16.44	15.51	13.08	15.18	15.33	13.04	16.31	8.10	14.24	14.89	9.29
16.12	13.23	13.89	16.90	15.72	13.45	16.93	16.26	12.40	16.88	8.66	13.80	15.31	9.28
16.32	13.00	13.42	16.18	15.45	13.16	16.77	15.96	12.02	16.60	8.43	13.43	14.83	8.88
16.84	13.60	13.88	16.86	15.81	14.18	17.50	18.37	12.62	17.02	9.22	14.61	15.40	9.53
16.64	13.40	13.73	16.68	15.56	13.90	17.42	18.28	12.36	16.98	9.00	14.67	15.52	9.41
16.66	13.52	13.89	16.79	16.00	13.20	16.93	18.75	12.63	17.06	9.43	14.10	15.58	9.59
16.59	13.47	14.00	16.64	16.14	13.40	16.91	18.59	12.61	17.13	9.26	13.95	15.53	9.60
16.73	14.20	15.64	17.29	16.87	15.59	20.00	18.14	13.14	17.21	10.35	15.11	16.49	10.47
16.59	13.66	15.23	17.01	16.06	14.43	18.67	17.82	12.50	17.40	9.41	14.49	15.89	9.85
17.49	14.23	14.09	17.69	16.50	14.24	17.90	18.08	13.88	17.48	10.49	15.06	16.29	10.64
17.96	14.28	14.50	17.65	17.06	14.60	18.10	18.75	14.28	17.92	10.73	15.33	16.29	11.13
17.09	13.72	14.70	16.96	16.13	14.60	18.00	19.62	12.98	17.90	10.46	15.16	15.95	9.75
17.48	13.89	14.53	16.97	15.99	14.64	18.27	19.37	13.01	17.83	10.53	15.08	15.90	9.87
16.56	14.13	14.15	17.31	16.45	13.98	18.12	17.73	13.08	17.38	9.81	15.13	16.28	10.35
17.28	14.66	14.53	17.81	16.29	14.42	18.47	17.83	13.59	17.90	10.26	15.52	16.65	10.77
15.90	13.23	13.52	16.74	15.81	14.17	15.78	18.39	12.59	16.56	9.41	14.74	15.48	10.16
15.90	13.23	13.52	16.74	15.81	14.17	15.78	18.39	12.59	16.56	9.41	14.74	15.48	10.16
