# Supplementary material for: The expression signatures in liver and adipose tissue from obese Göttingen Minipigs reveal a predisposition for healthy fat accumulation
Source: Nutr Diabetes. 2020 Mar 23;10:9. doi: 10.1038/s41387-020-0112-y (PMC7090036; doi:10.1038/s41387-020-0112-y)
Supplement: Supplementary file 6 — S5 Table [file 41387_2020_112_MOESM6_ESM.pdf]

S5. Raw qPCR data from the 4 relevant genes

Liver

Samples	TBP	CD36	FABP4	LPL	PPARG 1-2	PPARG1	PPARG2
320207.1	29.36	35.54	35.48	36.98	29.15	32.33	33.67
320207.2	30.13	36.29	36.86	36.98	30.3	33.34	34.87
319798.1	29.46	32.16	32.8	27.23	26.67	29.35	31
319798.2	29.91	33.29	33.67	27.83	27.54	29.03	30.93
320351.1	29.91	32.66	35.49	28.75	26.9	29.7	31.84
320351.2	30.27	33.09	36.12	29.09	27.07	29.98	32.52
319876.1	28.78	30.59	30.01	27.12	25.44	28.18	30.05
319876.2	29.13	30.62	30.39	27.47	26.02	29	29.87
320340.1	29.1	33.95	34.21	33.78	29.27	32.02	32.22
320340.2	29.84	33.95	34.9	33.22	30.29	32.51	34.79
221497.1	29.61	31.5	33.68	26.73	25.83	28.1	30.46
221497.2	29.23	31.79	33.78	26.94	25.74	27.96	30.5
319799.1	28.83	30.85	33.07	25.21	25.56	27.75	29.65
319799.2	29.55	31.73	34.02	26.06	26.18	28.71	30.49
319021.1	28.79	30.06	28.74	25.84	25.41	28.11	30.1
319021.2	29.23	30.23	29.09	26.31	25.85	28.79	30.74
320411.1	29.69	35.52	35.86	36.9	29.85	33.46	No Ct
320411.2	30.17	35.36	35.98	36.9	31.23	34.91	No Ct
319875.1	29.51	33.46	33.29	29.08	27.48	29.95	32.76
319875.2	29.3	33.37	33.25	29.3	28.08	30.23	32.38
319239.1	29.31	30.41	33.35	25.81	25.62	28.03	29.51
319239.2	29.27	30.35	33.15	26.02	25.69	28.13	30.3
221516.1	29.1	30.31	29.39	25.97	25.2	27.84	29.56
221516.2	29.73	30.88	30.25	26.71	25.89	28.58	30.28
319860.1	29.53	35.29	36	37.33	30.46	34.32	No Ct
319860.2	29.84	35.62	36.3	37.33	31.91	33.06	36.22
221538.1	28.97	31.85	33.27	27.77	26.11	28.92	30.74
221538.2	29.2	32.35	33.35	28.47	26.85	28.93	30.93
221510.1	29.48	31.62	33.24	26.38	25.16	27.47	29.2
221510.2	29.32	31.2	33.54	26.68	25.26	27.57	29.54
319703.1	28.81	31.5	30.85	26.85	25.06	27.87	29.59
319703.2	29.31	31.82	30.86	27.06	25.48	28.29	30.71
319778.1	29.43	35.64	33.21	37.03	30.45	32.22	33.18
319778.2	30.35	34.72	34.34	37.03	29.84	33.51	No Ct
319035.1	29.34	30.75	34.07	26.32	25.35	28.09	29.96
319035.2	29.06	31.13	33.64	26.15	25.49	28.06	30.79
320241.1	30.03	32.89	33.27	28.55	27.25	29.28	30.83
320241.2	30.37	32.79	33.38	29.3	27.52	30.86	30.78
319537.1	29.5	30.65	32.66	26.68	25.38	27.71	29.52
319537.2	29.24	30.82	33.16	26.94	25.27	28.31	29.67
319427.1	29.35	34.71	36.29	36.09	31	34.07	No Ct
319427.2	30.69	36.19	38.19	36.09	32.6	33.58	No Ct
319389.1	29.76	32.51	33.22	28.9	26.71	29.39	30.91
319389.2	29.66	33.18	33.65	28.8	26.62	29.34	31.34
320335.1	28.37	29.29	27.99	25.57	25.04	28.26	29.18
320335.2	28.8	29.82	28.3	26.16	25.47	28.66	30.48
319922.1	29.35	36.4	35.56	35.65	32.65	34.95	No Ct
319922.2	29.55	36.07	35.48	35.65	31.72	34.85	No Ct
320316.1	29.31	31.91	32	28.19	27.01	29.77	32.09
320316.2	29.5	32.68	32.18	28.61	27.88	29.97	32.16
320462.1	29.14	29.68	32.16	25.02	24.66	27.1	29.39
320462.2	29	29.93	32.25	25.7	25.15	27.6	29.2
320440.1	29.3	30.48	33.21	25.88	25.63	27.71	30.01
320440.2	29.24	30.64	33.95	25.83	25.66	27.77	29.83
320317.1	29.75	32.65	35.06	30.79	26.78	29.6	30.21

SAT

Samples	TBP	CD36	FABP4	LPL	PPARG 1-2	PPARG1	PPARG2
221516.1	30.29	22.45	18.10	19.46	27.37	26.35	24.80
221516.2	30.37	21.81	17.71	19.47	27.12	26.55	24.59
319021.1	30.12	22.47	17.97	20.20	27.68	27.00	25.39
319021.2	30.12	22.60	18.07	20.21	27.74	27.00	25.18
319357.1	30.67	22.97	18.26	20.17	27.27	27.01	25.03
319357.2	30.71	23.15	18.18	19.80	27.34	27.21	25.29
319677.1	30.85	23.07	18.31	19.46	27.19	26.66	24.36
319677.2	30.08	22.67	18.39	19.21	27.06	26.31	24.81
319703.1	29.92	21.81	17.58	19.36	26.88	26.42	25.24
319703.2	30.22	22.10	17.85	20.07	26.94	26.36	25.37
319876.1	30.51	22.27	17.94	20.14	27.68	26.46	26.31
319876.2	30.17	22.23	17.81	19.79	27.63	25.98	25.55
320462.1	30.34	22.03	19.34	19.78	27.15	26.32	24.92
320462.2	30.54	22.34	18.13	19.80	27.35	26.36	24.99
221497.1	30.54	22.51	17.93	19.27	27.50	26.46	24.48
221497.2	30.24	22.15	17.62	19.32	26.95	26.31	24.58
221510.1	30.18	22.24	18.11	19.45	26.66	26.27	24.47
221510.2	29.32	22.05	17.38	19.06	26.40	26.02	24.38
221538.1	29.84	21.97	17.66	19.97	26.73	26.10	24.34
221538.2	29.63	21.93	17.55	19.89	26.78	25.85	25.28
319035.1	30.59	22.77	18.41	20.54	27.85	26.76	25.49
319035.2	30.57	22.78	18.68	20.62	28.33	26.98	26.14
319239.1	29.45	21.61	17.96	19.83	26.60	25.91	24.27
319239.2	29.61	22.14	17.93	20.20	26.62	26.11	24.28
319389.1	30.06	22.06	18.57	19.14	26.75	25.84	24.53
319389.2	29.93	21.78	17.95	19.37	26.36	26.65	24.29
319798.1	29.14	21.33	17.63	19.24	26.80	26.08	24.49
319798.2	29.74	21.82	18.22	19.85	27.08	26.25	24.55
319799.1	30.14	22.89	18.82	19.89	27.31	26.94	25.04
319799.2	30.15	22.89	18.48	20.22	27.27	26.77	24.43
319875.1	29.91	21.88	17.58	19.40	27.04	26.26	24.46
319875.2	29.90	21.67	17.33	19.27	27.07	26.19	24.60
320241.1	30.16	22.45	18.55	20.55	27.49	26.85	25.44
320241.2	30.09	22.98	18.54	20.79	27.81	26.72	25.75
320316.1	29.70	21.47	17.78	20.33	27.54	26.85	25.40
320316.2	29.26	21.04	17.41	19.61	27.23	26.32	25.09
320317.1	30.53	22.38	18.56	20.77	27.58	26.86	25.52
320317.2	29.77	21.90	18.31	20.55	26.61	27.40	25.18
320351.1	29.65	22.08	18.24	20.02	26.69	26.36	24.02
320351.2	29.07	22.13	18.96	19.64	26.70	26.31	24.51
320440.1	30.05	21.94	17.92	19.64	27.71	27.29	25.65
320440.2	30.15	22.20	18.23	19.56	27.88	27.23	25.24
319427.1	30.85	24.22	20.07	22.05	28.49	27.97	26.55
319427.2	32.08	25.48	21.49	23.23	29.56	28.86	27.79
319778.1	30.27	23.45	18.77	20.03	27.46	26.96	25.46
319778.2	30.57	23.38	18.71	20.50	27.45	26.77	25.67
319860.1	29.94	22.62	18.22	19.24	26.52	26.45	24.61
319860.2	30.19	22.60	18.60	19.38	26.90	26.65	24.81
319922.1	29.98	22.13	18.03	19.37	26.46	26.33	24.61
319922.2	29.74	22.33	18.08	19.18	26.16	25.77	24.10
320207.1	29.74	22.50	18.03	18.29	26.27	25.94	24.29
320207.2	30.04	22.72	17.93	18.71	26.20	26.13	24.13
320340.1	30.12	21.69	17.22	19.28	26.57	25.95	24.87
320340.2	30.00	21.45	17.13	19.35	26.22	25.97	24.74
320411.1	30.29	23.32	19.42	20.49	28.29	28.00	26.17

VAT

Samples	TBP	CD36	FABP4	LPL	PPARG 1-2	PPARG1	PPARG2
221516.1	30.43	25.50	18.29	23.03	27.95	28.62	28.14
221516.2	30.22	25.22	18.71	22.94	27.91	28.46	27.78
319021.1	29.70	25.74	19.16	21.95	27.72	28.74	No Ct
319021.2	29.92	26.38	19.08	22.89	28.02	29.24	27.88
319357.1	29.11	22.89	16.88	20.73	26.19	27.26	26.01
319357.2	29.21	22.60	16.74	20.67	26.19	27.11	26.56
319677.1	32.46	28.26	20.79	24.21	29.14	30.23	29.12
319677.2	32.03	28.19	20.66	23.95	28.96	30.10	29.44
319703.1	29.18	23.69	17.94	20.60	25.83	27.72	26.36
319703.2	29.28	24.41	17.62	20.92	26.03	27.24	26.69
319876.1	32.45	26.92	20.02	23.25	28.85	30.03	29.35
319876.2	32.64	26.86	19.74	23.27	28.88	29.95	29.20
320462.1	29.02	23.40	16.45	20.43	26.14	27.21	26.83
320462.2	29.55	23.26	16.92	20.55	26.21	27.21	27.03
221497.1	29.43	24.88	17.92	21.93	27.18	27.96	No Ct
221497.2	29.91	24.58	18.05	22.02	27.33	28.06	28.05
221510.1	29.47	23.76	16.95	20.51	25.54	26.82	25.89
221510.2	29.55	23.79	17.24	20.29	25.52	27.03	26.23
221538.1	29.77	25.08	18.34	22.09	27.12	27.94	27.72
221538.2	30.01	24.65	18.50	22.20	27.24	27.75	28.20
319035.1	31.07	25.03	18.11	22.08	27.59	29.14	28.72
319035.2	29.68	23.83	16.87	20.78	26.10	27.50	27.19
319239.1	30.94	25.15	19.18	22.62	27.34	28.54	28.15
319239.2	29.87	24.69	18.53	21.91	26.81	28.11	27.49
319389.1	27.25	23.35	17.23	21.26	26.82	26.74	27.25
319389.2	27.37	23.03	17.43	21.30	26.41	26.73	27.69
319798.1	30.11	25.62	18.64	22.45	27.25	28.04	28.26
319798.2	29.83	25.29	19.07	22.93	27.29	28.56	28.44
319799.1	32.70	27.68	20.90	24.53	29.95	30.91	30.55
319799.2	32.84	27.87	20.87	24.52	30.64	31.10	30.31
319875.1	33.70	28.94	21.45	24.74	30.07	30.97	30.10
319875.2	33.00	27.75	21.10	24.62	29.16	30.42	29.57
320241.1	30.03	25.48	19.38	21.93	28.72	29.07	29.12
320241.2	29.69	25.29	19.14	21.71	27.91	28.40	28.32
320316.1	29.58	25.23	18.62	23.14	27.10	No Ct	28.10
320316.2	29.10	25.11	18.47	22.86	27.13	No Ct	27.60
320317.1	29.91	24.26	17.61	21.51	26.59	27.27	27.73
320317.2	29.25	23.32	17.05	21.27	26.12	27.10	27.15
320351.1	29.84	23.89	17.54	21.18	26.13	27.46	27.14
320351.2	29.84	24.39	17.70	20.38	25.95	27.28	26.62
320440.1	30.03	23.55	17.48	21.25	26.11	27.36	26.90
320440.2	29.50	23.34	17.59	20.93	26.29	27.13	27.37
319427.1	31.08	24.72	18.43	21.73	26.18	27.66	26.52
319427.2	30.34	24.12	18.19	21.11	25.70	27.42	26.43
319778.1	30.48	25.53	19.38	21.96	26.93	28.15	27.40
319778.2	30.93	25.41	19.48	21.94	26.81	27.87	27.88
319922.1	29.55	23.31	17.92	19.76	25.12	27.01	26.45
319922.2	29.73	23.68	17.87	19.84	25.15	26.69	26.19
320207.1	30.39	24.75	18.32	20.61	26.50	27.53	27.00
320207.2	30.56	24.75	18.68	20.85	26.91	28.06	27.33
320411.1	29.95	25.47	20.37	23.42	27.72	30.45	31.19
320411.2	29.95	25.47	20.11	23.42	27.72	28.80	28.74

320317.2	30.15	32.7	35.75	31.36	27.22	30.04	30.52	320411.2	30.08	23.16	18.93	20.40	28.01	27.47	25.96
319677.1	29.77	30.89	32.87	26.5	25.49	27.85	29.27								
319677.2	30.44	32.09	33.26	28.18	26.59	28.95	31.22								

raw qPCR data efficiency corrected

ref gene
